# Insertion sequences and other mobile elements associated with antibiotic resistance genes in *Enterococcus* isolates from an inpatient with prolonged bacteraemia

**DOI:** 10.1099/mgen.0.000855

**Published:** 2022-08-03

**Authors:** Zulema Udaondo, Kaleb Z. Abram, Atul Kothari, Se-Ran Jun

**Affiliations:** ^1^​ Department of Biomedical Informatics, University of Arkansas for Medical Sciences, Little Rock, Arkansas, USA; ^2^​ Arkansas Dept of Health, Healthcare Associated Infections and Outbreak Response Sections, Little Rock, AR 72205, USA

**Keywords:** antibiotic resistance gene, composite transposon, daptomycin, enterococcus, insertion sequences, Oxford nanopore sequencing, WGS

## Abstract

Insertion sequences (ISs) and other transposable elements are associated with the mobilization of antibiotic resistance determinants and the modulation of pathogenic characteristics. In this work, we aimed to investigate the association between ISs and antibiotic resistance genes, and their role in the dissemination and modification of the antibiotic-resistant phenotype. To that end, we leveraged fully resolved *Enterococcus faecium* and *Enterococcus faecalis* genomes of isolates collected over 5 days from an inpatient with prolonged bacteraemia. Isolates from both species harboured similar IS family content but showed significant species-dependent differences in copy number and arrangements of ISs throughout their replicons. Here, we describe two inter-specific IS-mediated recombination events and IS-mediated excision events in plasmids of *E. faecium* isolates. We also characterize a novel arrangement of the ISs in a Tn1546-like transposon in *E. faecalis* isolates likely implicated in a vancomycin genotype–phenotype discrepancy. Furthermore, an extended analysis revealed a novel association between daptomycin resistance mutations in *liaSR* genes and a putative composite transposon in *E. faecium *, offering a new paradigm for the study of daptomycin resistance and novel insights into its dissemination. In conclusion, our study highlights the role ISs and other transposable elements play in the rapid adaptation and response to clinically relevant stresses such as aggressive antibiotic treatment in enterococci.

## Data Summary

All supplementary tables are available on Zenodo: https://zenodo.org/record/6402290#.YkXJzSSZM-w under DOI:10.5281/zenodo.6402290. Figs S6, S7 and S9 are available on Zenodo: https://doi.org/10.5281/zenodo.6513079 under DOI: 10.5281/zenodo.6513079

Raw sequencing data, genome assemblies and functional annotations for the isolates described in this study are available under the BioProject accession number PRJNA735268 in the National Center for Biotechnology Information (NCBI) database.

**Impact Statement**

Insertion sequences (ISs) and transposable elements are associated with the mobilization of antibiotic resistance determinants (ARDs) and the modulation of pathogenic characteristics; however, there is a lack of information on the association between ISs and ARDs and other aspects related to the nature, maintenance and transmission mechanisms of ISs in bacterial genomes. In this work, we present six isolates from two bacterial species, sharing the same niche (a patient with bacteraemia) and collected over a 4-day period. The isolates from both species showed a very similar IS family content, but a drastically different copy number and arrangement of ISs along their replicons. In this work we describe several IS-mediated rearrangements, deletions and two putative IS-mediated recombination events between *Enterococcus faecalis* and *Enterococcus faecium* species. We also describe a previously undescribed association between daptomycin resistance mutations in *liaSR* genes and a putative compound transposon in *E. faecium* genomes. Our results highlight the important role ISs and other transposable elements play in the rapid genomic adaptation and response of enterococci to clinically relevant stresses.

## Introduction

*Enterococcus* spp. are ubiquitous bacteria that live as commensals in the gastrointestinal tract of humans and other mammals [1, 2]. Their genetic plasticity enables them to acquire genetic determinants [3] to thrive in modern hospital environments, where they often cause severe infectious diseases, especially in immunocompromised patients [2, 4]. Multidrug-resistant enterococci not only play an important role as nosocomial pathogens, but also are a reservoir of genes encoding antibiotic resistance that can be transferred to other commensal micro-organisms, increasing the risk of colonization and infection [4, 5].

In recent years, vancomycin-resistant *Enterococcus* (VRE) infection cases have increased rapidly around the world. Consequently, a number of studies have examined the epidemiology [6, 7], population dynamics [8–10] and evolutionary landscape [11–13] of *Enterococcus *. However, there is still a lack of knowledge about the level of influence that plasmids and mobile genetic elements have on the persistence and spread of VRE strains [14–16]. This *is partly* due to the presence of repetitive regions in genomes of *Enterococcus* that hinder full genome reconstruction [17, 18]. Some of these repetitive sequences, such as insertion sequences (ISs), have been considered to be pathogenicity enhancers [5, 19, 20]. Therefore, analysing these elements is of vital importance to determine the key characteristics of VRE strains, such as the level of antimicrobial resistance and virulence potential, or to identify pathogenic variants.

**Fig. 1.**
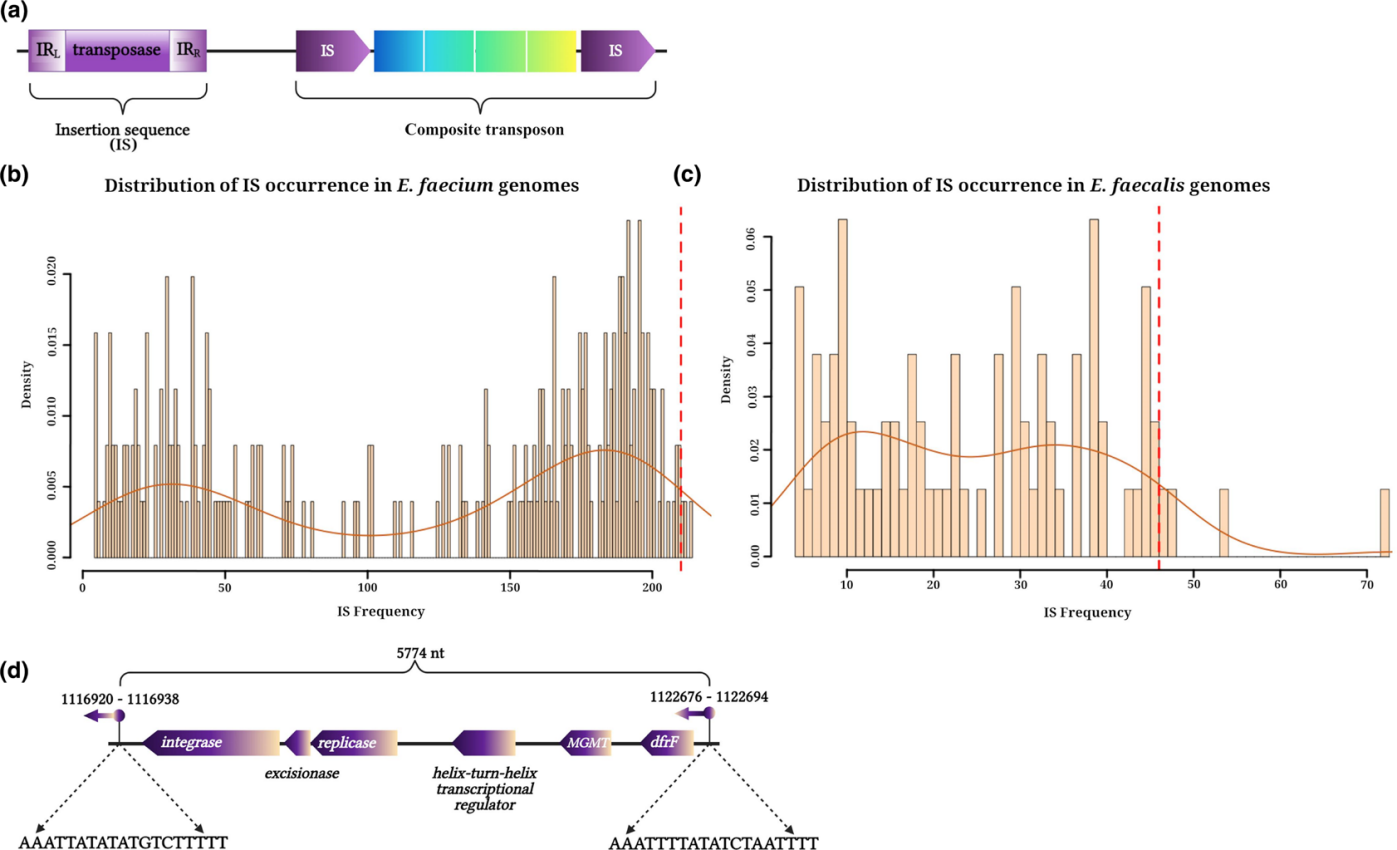
(a) Schematic representations of an insertion sequence and a composite transposon. The IS elements in the composite transposon are represented according to the orientation of their encoded transposase. Both IS elements can also be oriented inversely. (b, c) Distribution of IS occurrences in (**b**) 252 *E. faecium* complete genome sequences and (**c**) 79 *E. faecalis* using ISEScan. The *y*-axis indicates the density of each IS frequency, where density is approximately the number of occurrences for a given IS divided by the range of IS frequencies (210 for *E. faecium,* 69 for *E. faecalis *). The sum of the density values of the plot is equal to 1. The *x*-axis represents the count of ISs present in all genomes of the corresponding species. The means of the number of ISs annotated in the three *E. faecalis* (**b**) and in the three *E. faecium* isolates (**c**) from this study are marked with a red dashed line. (**d)** Schematic representation of a novel transposable element found in *E. faecalis* isolates from this study. This figure was generated using BioRender.

ISs are small autonomous transposable elements, generally flanked by short terminal inverted repeats, that usually carry at least one transposase (Tn) gene whose product mediates the transposition process (Fig. 1a) [21, 22]. ISs are not only able to move themselves to different locations within genomes through various mechanisms [23], but are also able to travel horizontally between genomes as part of other mobile genetic elements such as bacteriophages and plasmids [21, 24]. Transposases are mainly classified according to their transposition chemistry related to their catalytic domain topology and also based on their mode of transposition (replicative or non-replicative) [22–24]. Furthermore, DNA segments carrying several genes can move together as a single unit when flanked at both ends by two ISs in the same or a properly oriented direction [21, 22]. These larger transposable units are called compound or composite transposons (Fig. 1a). Well-known examples of composite transposons that carry antibiotic resistance determinants (ARDs) are Tn5 (kanamycin resistance) [25], Tn9 (chloramphenicol resistance) [26], Tn10 (tetracycline resistance) [27] and Tn4001 (gentamicin resistance) [28]. The important roles of ISs in the transmission, inactivation and modification of the resistant genotype and virulence of prokaryotic strains have been reviewed in previous studies [21, 24]. However, most of these studies were performed using contigs or scaffolds from genome assemblies that might be missing a number of repetitive ISs.

It has been established that environmental stresses can induce elevated rates of IS transposition activities, also known as ‘transposition bursts’ [29]. However, a comprehensive analysis of the transposase activity among isolates that persist in the same stressful environment, such as high levels of antibiotic exposure, is lacking. In the same vein, it is not clear what role transposable elements play in large-scale genome variations due to IS-mediated genetic rearrangements and their association with ARDs in bacterial cells. This study was conducted using full genome assemblies from six isolates, collected from the blood of an inpatient with bloodstream co-infection of *Enterococcus* and diagnosed with acute lymphocytic leukaemia. We performed genome-wide computational analysis in order to provide insights into the landscape of transposable elements that could be involved in disseminating multidrug resistance. Our analyses were focused on the ISs found in the proximity of ARDs and on the genetic changes observed as a consequence of putative transposition activity at the species and inter-species level.

## Methods

### Sample collection and vancomycin susceptibility testing

The six blood samples were collected at different time points from an inpatient at the University of Arkansas for Medical Sciences (UAMS). This study involved blood samples which were sent to the Clinical Microbiology Laboratory for clinical use, not for this study. Therefore, there was no direct contact with the study participants. Cultures were grown on blood agar plates and processed on the BacT/ALERT 3D system (bioMérieux, Durham, NC, USA). The Accelerate Pheno system (Accelerate Diagnostics, Tucson, AZ, USA) was used for the identification and susceptibility testing of *Enterococcus *-positive blood cultures. For repeated positives, identification and susceptibility testing was confirmed using Vitek 2 MS and Vitek 2 systems. The vancomycin-resistant phenotype of the six isolates was obtained using E-tests (bioMérieux) and antimicrobial susceptibility tests. Results were interpreted using the M100 Clinical and Laboratory Standards Institute (CLSI) standards [30].

### Whole-genome sequencing

Genomic DNA was extracted and sequenced as described elsewhere [31, 32]. Briefly, genomic DNA was extracted from VRE colonies using the Quick-DNA Fungal/Bacterial kit (Zymo Research, Irvine, CA, USA). A NanoDrop Spectrophotometer (Thermo Fisher, Waltham, MA, USA) was used to determine the purity of the extracted DNA by measuring the A260/280 and A260/230 ratios. The integrity and quantity of DNA were determined using an Agilent 2200 TapeStation (Agilent, Santa Clara, CA, USA) and Qubit 3.0 assay (Thermo Fisher), respectively. Purified DNA was aliquoted into two tubes for MinION (Oxford Nanopore Technologies, Oxford, UK) and Illumina (San Diego, CA, USA) sequencing. Oxford Nanopore Technologies (ONT) sequencing libraries were prepared using a PCR-free method of multiplexing samples with the Rapid Barcoding kit (SQK-RBK004, ONT), and sequencing of the barcoded DNA was performed on a single R9.4/FLO-MIN106 ONT flow cell on the MinION (version Mk1B, ONT) for 48 h. Illumina libraries for the six isolates were also sequenced using the NextSeq 550 platform at the UAMS Myeloma Center. Adapters were trimmed using fastp (version 0.19.5) [33], with default settings. Trimmomatic (version 0.38) [34] was used to remove poor-quality reads. The quality of pre- and post-processed reads was assessed with the FastQC tool (version 0.11.8) [35].

### Base calling and assembling

Base calling of Nanopore reads was performed using Guppy v4.4.2 [36], with --min_qscore 8. Libraries were demultiplexed using guppy_barcoder from Guppy v4.4.2. Porechop v0.2.3 (https://github.com/rrwick/Porechop) was used to remove adapters, and NanoFilt from NanoPack [37] was used for further quality filtering. Read quality was examined with NanoStat v1.2.0 from NanoPack [37]. Two different strategies were established to obtain fully resolved assemblies depending on the species. For *E. faecium* isolates, we used Unicycler v0.4.8 [38] hybrid assembler, with scaffolds generated by Canu v2.1.1 [39] and Illumina data as inputs. Fully resolved assemblies for *E. faecalis* isolates were obtained using Flye v2.8.2 assembler [40] with Oxford Nanopore reads, with two rounds of polishing using the Oxford Nanopore long-reads with Racon v1.4.20 [41] and Medaka v1.2.1 (https://github.com/nanoporetech/medaka). For both *E. faecium* and *E. faecalis *, final polishing with Illumina data was performed through two rounds of Pilon v1.23 [42].

### Functional antimicrobial resistance and mobilome annotation

Functional annotation of the six isolates was performed using Prokka v1.14.6 [43] with the --rnammer option. Functional annotation of plasmid replicons and other chromosomal regions of interest was manually reannotated using PsiBlast and blastp from the National Center for Biotechnology Information (NCBI) and *Enterococcus* nr/nt databases. ARDs were annotated with Resistance Gene Identifier (RGI) software against the Comprehensive Antibiotic Resistance Database (CARD) [44]. ISEScan v1.7.2.1 [45] and the ISfinder database (https://isfinder.biotoul.fr/) [46] were used to identify transposable elements and their inverted repeat sequences. Plasmids were classified using PlasmidFinder2.0 [47]. CGview server (version beta) [48] was used to plot the functional and structural characteristics of the plasmids in this study.

### Multilocus sequence typing (MLST)

MLST analysis was performed using mlst tool (T. Seemann, mlst Github https://github.com/tseemann/mlst) with public PubMLST typing schemes [49].

### Comparative genome analysis

All replicons were divided into fragments of 5000 bp with an overlap of 3000 bp (2 000 step size) and compared via blastn v2.9.0 [50]. Bidirectional best hits between replicons from different isolates were identified using best bit score, e-value and percentage of sequence similarity. Mash v2.3 [51] with k=21 was used to determine genetic distances between isolates where sketch option with -s 10 000 was used to increase the sensitivity as described elsewhere [52]. Snippy (https://github.com/tseemann/snippy) was used to analyse the coreSNP of the six isolates. Figures were prepared using Clinker v0.0.20 [53], pyGenomeTracks [54] and BioRender (https://biorender.com/).

The genome sequences of the six isolates were aligned using minimap2 [55] with parameter settings of -x asm5 and then visualized using dotPlotly using the parameters -m 1000 -q 1000 s -t -l (https://github.com/tpoorten/dotPlotly). Artemis Comparison Tool (release 18.0.1) [56] was used to visualize the blastn alignments of the replicon sequences of the six isolates by species. Multiple genome alignments of the entire replicons was performed using Mugsy software v1.2.3 [57]. Multiple genome alignments of selected replicons were visualized using Gmaj software (https://globin.bx.psu.edu/dist/gmaj/).

All complete genomes available in GenBank for *E. faecalis* and *E. faecium* species were downloaded in December 2020.

## Results

### Fully resolved replicons in *E. faecalis* and *E. faecium* isolates obtained from hybrid *de novo* assembly

The six *Enterococcus* isolates from one patient with prolonged bacteraemia were collected from blood samples over a period of 4 days and identified as *E. faecalis* (isolates UAMS_EL53, UAMS_EL54 and UAMS_EL56) or *E. faecium* (UAMS_EF55, UAMS_EF57 and UAMS_EF58). Vancomycin susceptibility testing showed a susceptible phenotype for all *E. faecalis* isolates [minimum inhibitory concentration (MIC) ≤2 µg ml^−1^]. However, all *E. faecium* isolates were phenotypically resistant to vancomycin, with an MIC >16 µg ml^−1^ for the first two isolates and an MIC ≥64 µg ml^−1^ for the last collected isolate (UAMS_EF58) (Table S1). Two different *de novo* hybrid assembly strategies, depending on species (described in the Methods section) were used to generate fully resolved assemblies for all six isolates. Thus, complete circular replicons were obtained for all the chromosomes and for almost all of the plasmids for the six isolates in this study (Table 1).

**Table 1.** Summary of *de novo* assembly results for *E. faecalis* and *E. faecium* isolates

Isolate	UAMS_EL53	UAMS_EL54	UAMS_EL56
**Collection order**	1	2	4
**Species**	* E. faecalis *	* E. faecalis *	* E. faecalis *
**Chromosome**	3 030005 c	3 030422 c	3 031832 c
**pUAMSEL1**	119720 c	119714 c	119713 c
**pUAMSEL2**	55301 c	55304 c	55288 c
**pUAMSEL3**	53420 c	53047 c	53193 c
**PUAMSEL4 (*vanA***)	33652c×3	33656c×3	33659c×3
**Isolate**	**UAMS_EF55**	**UAMS_EF57**	**UAMS_EF58**
**Collection order**	3	5	6
**Species**	* E. faecium *	* E. faecium *	* E. faecium *
**Chromosome**	2 841245 c	2 841244 c	2 841249 c
**pUAMSEF1(a,b**)	a.264192 c	b.205436 c	b.205407 c
**pUAMSEF2**	79 782	79 769	79 679
**pUAMSEF3**	*	57509 c	57509 c
**pUAMSEF4 (*vanA***)	41819c×3	41819c×3	34693c×3
**pUAMSEF5**	4374 c	4374 c	4374 c

c, circularized replicon according to assembler; ×3, inferred multiplicity based on normalized read depth per contig.

*, plasmid not detected.

Chromosome size for *E. faecalis* isolates [classified as ST6 by multilocus sequence typing (MLST)] were slightly larger (~3 Mb) than those in *E. faecium* isolates (~2.8 Mb) (classified as ST736 by MLST). Four to five complete plasmids were identified in all isolates (Table 1, Fig. S1). All six isolates harboured plasmids (pUAMSEF4 for *E. faecium* isolates and pUAMSEL4 for *E. faecalis* isolates) with a complete cluster of genes conferring vancomycin resistance (*vanR*, *vanS*, *vanH*, *vanA*, *vanX*, *vanY* and *vanZ*; phenotype vanA), revealing a genotype–phenotype discrepancy in *E. faecalis* isolates. It is worth noting that plasmids carrying vancomycin resistance genes presented greater differences in read depth compared to the other replicons in this study. The multiplicity of *vanA* replicons was inferred to be triplicated in all assemblies based on the normalized read depth (Table 1). Plasmid sequence comparison analyses showed that the *vanA* plasmid from *E. faecalis* is most similar to the *vanA* plasmid from *E. faecium* (Fig. S2A). Whole-genome distances obtained using Mash [51] on the six isolates of the study showed that the last two collected isolates for each species (UAMS_EL56, UAMS_EF58) had the lowest genetic similarity to the other isolates of their respective species (Fig. S2b). Mash distances between isolates from different species were ~0.18.

### Arrangement, copy number and association of insertion sequences with antibiotic resistance genes differ in *E. faecalis* and *E. faecium* isolates

Detailed analysis of the ISs annotated by ISEScan [45] and ISfinder [46] in our 6 isolates identified 11 different IS families in *E. faecalis* isolates, including 1 putative novel IS family, and 12 IS families in *E. faecium* isolates (Tables 2, S2 and S3). The copy number of the IS families identified in both species varied considerably, with these elements being 5 times more abundant in *E. faecium* isolates (~210 ISs per isolate) than in *E. faecalis* isolates (~46 ISs per isolate). In *E. faecalis* isolates, the IS*256* family (*n*=12 in UAMS_EL53 and UAMS_EL54 and *n*=13 in UAMS_EL56) was the most abundant IS, followed by ISs from the IS*6* (*n=*9 in each isolate) and IS*3* families (*n*=9 in each isolate). In *E. faecium* isolates, the IS*L3* family (*n*=61) was the most abundant, with elements mainly distributed throughout the chromosome, followed by elements from families IS*256* (*n*=35) and IS*30* (*n*=27). Sequences from the IS*66* and IS*1380* families were found in all *E. faecium* isolates but not in *E. faecalis *. The IS elements identified in isolates from both species were distributed evenly along the chromosome (Table S2), but they were also found distributed among three of the four identified plasmids in *E. faecalis* isolates and in four of the five plasmids in *E. faecium *. The copy number of the annotated ISs was stable in all three *E. faecalis* isolates with the exception of one additional element from the IS*256* family identified in the chromosome of UAMS_EL56. This IS*256* element was located in an area surrounded by other ISs from IS*256* and IS*6* families, in which the only characterized gene was choloylglycine hydrolase family protein (Table S3)

**Table 2.** Summary of the number of annotated insertion sequences per isolate using ISEScan

IS family	UAMS_EL53	UAMS_EL54	UAMS_EL56	UAMS_EF55	UAMS_EF57	UAMS_EF58
**IS3**	9	9	9	26	26	26
**IS6**	9	9	9	20	19	19
**IS21**	4	4	4	2	2	2
**IS30**	3	3	3	27	27	27
**IS66**	0	0	0	7	7	7
**IS110**	1	1	1	12	12	12
**IS200/IS605**	1	1	1	5	5	5
**IS256**	12	12	13	35	35	35
**IS982**	3	3	3	8	8	8
**IS1182**	1	1	1	6	6	5
**IS1380**	0	0	0	2	2	2
**ISL3**	1	1	1	61	61	61
**New**	2	2	2	0	0	0
**Total no.**	46	46	47	211	210	209

Genetic variability can be useful to withstand stressful conditions [24, 58]. Rates of IS transposition can be increased by environmental factors, such as changes in the host environment and the presence of subinhibitory concentrations of antibiotics that serve as a signal to promote genetic variability [59]. To place the number of ISs found in our isolates in a global context, we compared the distribution of the number of annotated ISs in a set of complete *E. faecalis* and *E. faecium* genomes downloaded from the GenBank and our six isolates. Fig. 1b indicates the distribution of the number of ISs annotated in 252 complete *E. faecium* genome assemblies where the mean of the number of ISs annotated in our three *E. faecium* isolates is represented with a red line at the extreme right of the distribution (Fig. 1b). The same pattern was observed in *E. faecalis *, where only 3 out of 79 complete genomes had more ISs than our clinical isolates (Fig. 1c). Both species showed a bimodal distribution of annotated ISs. This bimodal distribution was accentuated in *E. faecium* strains, where most of the annotated genomes had a relatively small number of annotated ISs (*n*≤50) or over 150 annotated ISs (*n*≥190). However, we could not recover metadata related to the environmental source of most of the strains in this analysis, which prevented us from establishing correlations between the number of annotated ISs and the environmental source.

Considering a genetic neighbourhood of 10 upstream and downstream annotated open reading frames (ORFs), 14 of 21 annotated ARDs were found in the proximity of at least 1 IS in our 3 *E. faecalis* isolates (Table S3). Further analysis of the possible association between ISs and ARDs identified a previously unknown TE of ~5781 bp; this TE occurred twice at two different locations (with >99 % sequence similarity, 100 % coverage) in the chromosome of all our *E. faecalis* isolates (Table S2). The novel transposable element harboured genes involved in its integration and excision, including genes for integrase, excisionase, replicase, a helix–turn–helix transcriptional regulator, methylated-DNA–protein-cysteine methyltransferase (MGMT) and trimethoprim-resistant dihydrofolate reductase (*dfrF*), which is involved in the resistance to diaminopyrimidine antibiotics (Fig. 1d). The number of cargo genes and the length of this novel element suggest that it is a unit transposon rather than an IS element. Unit transposons are larger than IS elements, as they may carry several passenger genes, usually ARDs, bound by inverted repeats [22, 60]. Sequence similarity analysis of the entire region of 5781 bp against the NCBI database showed that this novel transposable element is 100 % conserved and exists in multiple copies in the genomes of other *E. faecalis *, including 28157_4, 27725_1 and 27688_1 strains. These *E. faecalis* genomes were sequenced using long and short reads and assembled following a hybrid *de novo* strategy (NCBI BioProject: PRJEB40976 from the University Medical Center of Utrecht). This transposable element could therefore be more widespread in *E. faecalis *, but its existence might have been concealed due to the lack of high-quality and complete assemblies for this species. According to CARD Prevalence v3.0.8 data, which were based on sequence data acquired from NCBI on 13 August 2020, the prevalence of the *dfrF* ARD in *E. faecalis* is low and found in only 4.03 % of all whole-genome sequences in this database (818 *E. faecalis* genomes). However, the prevalence of this gene in *E. faecium* species was 27.59 % (1468 *E. faecium* genomes). Indeed, *E. faecium* isolates from our study harboured several copies of *dfrF*. Searches of the novel transposable element family found in our *E. faecalis* isolates against the *E. faecium* isolates of our study resulted in *dfrF–dfrF* gene alignments with >99 % sequence similarity and 100 % coverage. But this was not the case for the other genes described in the transposable element from *E. faecalis* isolates. Sequence-based searches using the web resource for prokaryotic transposable elements, TnCentral [61] and tn_dna.seq database, yielded one significant alignment with a region of the Tn559 family (subclass Tn554) from *Staphylococcus aureus* ST398 [62]. This novel element was the only IS found in the genetic neighbourhood (defined as 10 upstream and downstream annotated ORFs) of ARDs in the chromosome of *E. faecalis* isolates.

We observed a different scenario when analysing the genetic neighbourhood of ARDs in our *E. faecium* isolates, where 20 out of 21 annotated ARDs had at least 1 IS in their proximity (±10 annotated ORFs). We also observed that IS elements in our *E. faecium* isolates were generally arranged as arrays. IS arrays consist of agglomerations of ISs in which several copies from the same IS family are close to each other, suggesting a high level of replicative transposition [63]. Regarding their relationship with ARDs, we observed that the MFS transporter permease gene *efmA* was the only ARD in *E. faecium* that did not have IS elements in its proximity.

**Fig. 2.**
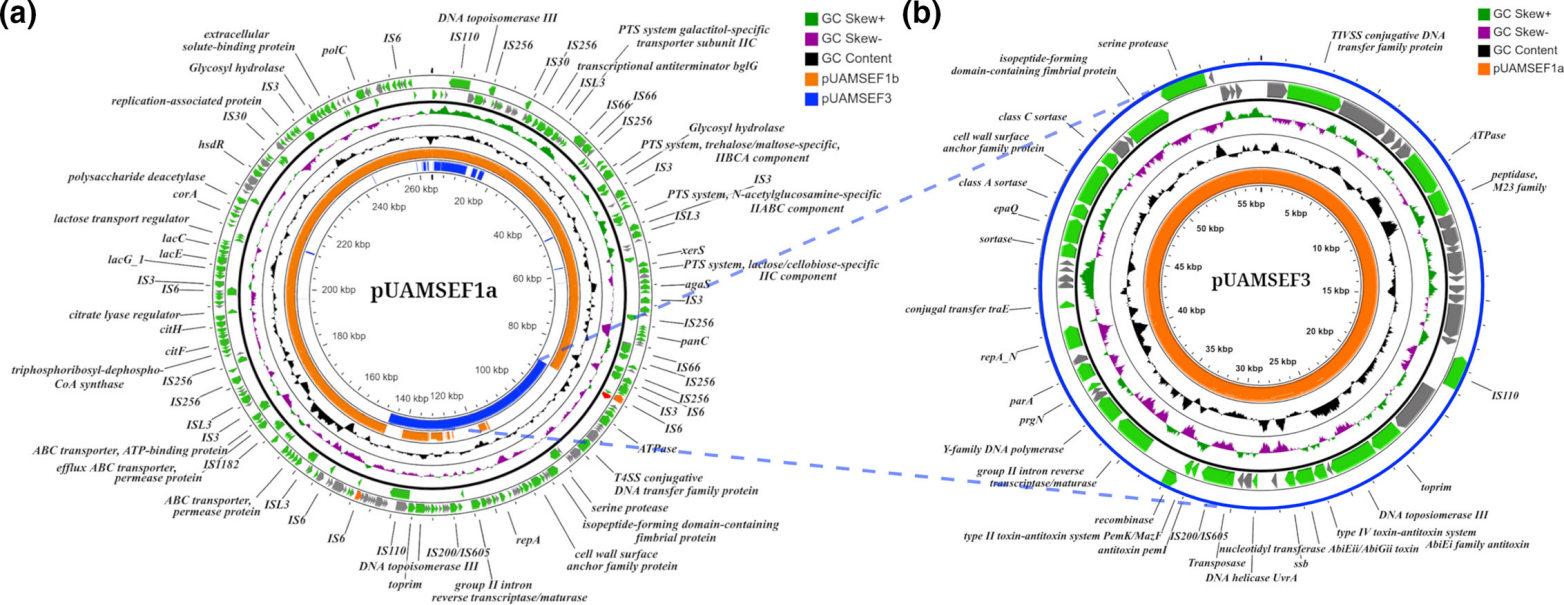
(a) Plasmid pUAMSEF1a from the first collected isolate of *E. faecium *, UAMS_EF55.** (b)** Plasmid pUAMSEF3, product of IS-mediated excision from pUAMSEF1a, present in UAMS_EF57 and UAMS_EF58 isolates.

Differences in IS copy number between our three *E. faecium* isolates (Table 2, Table S2, Table S3) were directly related to reorganizations in modular plasmids caused by IS-mediated deletions in the form of composite transposons (Fig. 1a, Fig. 2). The conjugative plasmid pUAMSEF1a is a RepA_N megaplasmid (>260 kb) with a strikingly high number of IS sequences (*n*=59) (Table 1, Table S2, Fig. 2a) that was only present in the *E. faecium* isolate UAMS_EF55. Despite the high number of IS families identified in this plasmid, only one ARD (aminoglycoside acetyltransferase, *aacA-aphD*) was found in pUAMSEF1a. However, there was a high number of efflux pumps, transporters and phosphotransferase systems, along with several genes related to plasmid maintenance, replication and transfer, such as several toprim genes; DNA primases, topoisomerases and resolvases; conjugal proteins; *tra* genes; and type IV secretion system components. Although pUAMSEF1a was only identified in the UAMS_EF55 isolate, UAMS_EF57 and UAMS_EF58 harboured two smaller plasmids (pUAMSEF1b and pUAMSEF3), which resulted from the excision of pUAMSEF1a (Figs 2a and S3, Table S3). The smaller plasmid product of pUAMSEF1a excision consisted of the segment bounded by two ISs in the same orientation, from the IS6 family in pUAMEF1a (specifically, two IS*1297* elements, first described in *Leuconostoc mesenteroides* subsp *. dextranicum* NZDRI2218) [64]. This circularized section constituted in its entirety the replicon pUAMSEF3 present in the two other *E. faecium* isolates (UAMS_EF57 and UAMS_EF58) that were collected just 1 and 2 days later, respectively, showing the high genetic activity of *E. faecium* isolates in this background. One of the two flanking IS6 elements was preserved in pUAMSEF1b (Fig. S4) after excision. However, both flanking IS6 elements were lost in the smaller plasmid pUAMSEF3 after the IS-mediated excision event. The plasmid pUAMSEF1b, found in UAMS_EF57 and UAMS_EF58 isolates, consisted of a RepA_N replicon of ~205 400 bp, which is the largest circularized segment remaining after the IS-mediated excision of pUAMSEF3 (orange inner ring in Fig. 2a). It is worth noting that another putative IS-mediated excision event was identified in the vancomycin resistance plasmid pUAMSEF4 in our *E. faecium* isolates; this is described in the section dedicated to the vancomycin plasmids.

### Daptomycin resistance mutations in *Liasr* genes are associated with a putative composite transposon in *E. faecium* isolates

Among the ISs inferred in the previous section, some ISs in the vicinity of ARDs in *E. faecium* were identified as potential composite transposons. Note that experimental evidence for transposition of these IS-bounded structures has not been provided in this work.

**Fig. 3.**
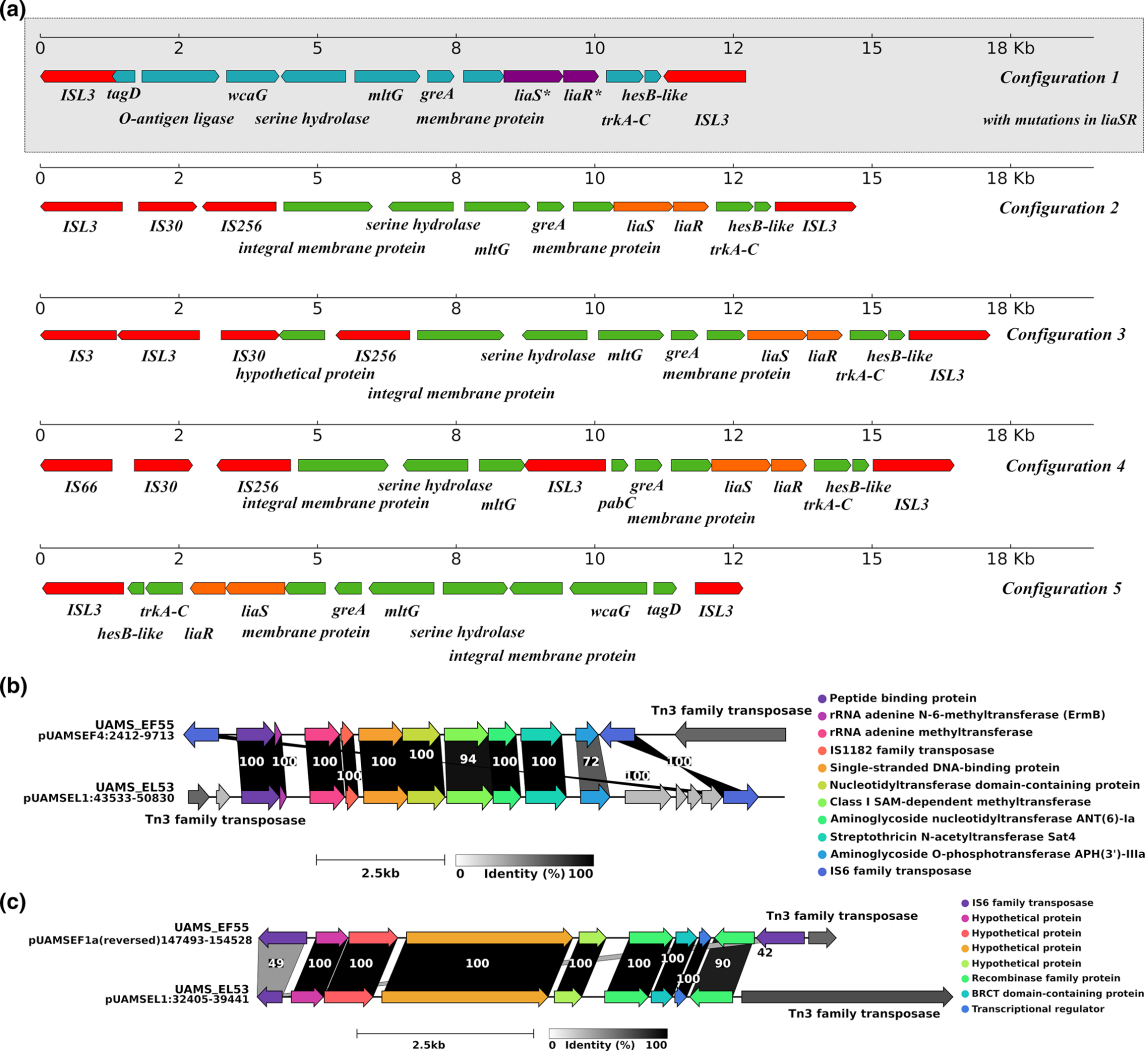
(a) IS configurations surrounding *liaSR* genes in 252 complete *E. faecium* genomes. Configuration 1, highlighted in grey, was only found in *E. faecium* with mutations in *liaSR* genes (LiaS^T120A^ and LiaR^W73C^). Mutated *liaSR* genes are marked with (*) in the figure.** (b)** A putative IS-mediated recombination event between *E. faecalis* and *E. faecium* isolates with *emrB* genes and linkage genes *aad [6]-sat4-aph(3’)-IIIa*. (**c)** A putative IS-mediated recombination event between pUAMSEF1a and pUAMSEL1 from *E. faecalis* and *E. faecium* isolates.

A noteworthy example of a putative composite transposon is the one found in the three *E. faecium* isolates formed by two IS*L3* elements identically oriented surrounding *liaSR* genes. LiaFSR is a three-component regulatory system involved in cell envelope homeostasis. As previously reported in multiple studies [65, 66], amino acid substitutions in LiaR (W73C) and LiaS (T120A) are associated with daptomycin (DAP)-resistant phenotype in *E. faecium *. Notably, all of our *E. faecium* isolates carried the LiaR^W73C^ and LiaS^T120A^ mutations. To test the hypothesis that there is an association between this IS*L3*-bounded structure and the presence of mutations associated with DAP resistance in *liaSR*, we extended our investigation to all complete, publicly accessible *E. faecium* genomes. Among 249 complete *E. faecium* genomes, we observed that 33 genomes carried both LiaR^W73C^ and LiaS^T120A^ mutations. Consistently, all 33 genomes also contained the same IS*L3*-bounded structure found in our *E. faecium* isolates (Fig. 3a). In detail, this putative composite transposon is composed of 2 IS*L3* sequences identically oriented surrounding 11 other genes in almost all cases (Figure S5, Table S4). The genes flanked by the IS*L3* elements were identified as *tagD*, encoding a glycerol-3-phosphate cytidylyltransferase, which is central to the synthesis of teichoic acids in the cell wall of Gram-positive bacteria [67]; an O-antigen ligase family protein gene involved in lipopolysaccharide biosynthesis [68]; *wcaG*, encoding an epimerase; a serine hydrolase gene; *mltG*, encoding an inner membrane endolytic transglycosylase capable of cleaving at internal positions within a glycan polymer [66]; *greA*, encoding a transcription elongation factor involved in bacterial invasion in other genera [69]; a cell wall-active antibiotic-response protein gene; *liaS* and *liaR*; *trkA-C*, encoding a potassium-uptake protein; and *hesB*, encoding an iron–sulfur cluster biosynthesis protein.

As observed previously [32, 70], the presence of LiaR^W73C^ and LiaS^T120A^ mutations does not always result in DAP-resistant phenotype, but it has been directly related to poor bactericidal activity and further development of DAP-resistant phenotype when DAP therapy is continued [32, 71, 72]. To confirm that the arrangement of the putative composite transposition could be associated with the presence of LiaR^W73C^ and LiaS^T120A^ substitutions in *E. faecium* genomes, we surveyed a region of ~15000 bp that included *liaSR* genes in the other 216 complete *E. faecium* genomes that did not carry *liaSR* mutations. Most of these genomes (*n*=116) did not harbour any IS*L3* in the proximity of *liaSR* genes. Among the strains with at least two ISs in the area surrounding *liaSR* genes (*n*=39), we identified four main IS-associated gene arrangements (Fig. 3a). The most prevalent arrangement was configuration 2, which was present in 26 out of 249 surveyed genomes, followed by configuration 3, found in 5 of the genomes. Most of the configurations contained variations in the copy number of some of the annotated ISs (mainly of IS*256* in configuration 2, which was duplicated in some strains). However, none of the configurations exhibited the pseudo-compound transposon-like structure found in our isolates with mutations in *liaSR* genes, flanked by identically oriented IS elements from the IS*L3* family.

### *E. faecalis* and *E. faecium* isolates harbour two regions with putative IS-mediated recombination events

Sequence similarity analysis of all ISs found in the six isolates showed that the DNA sequence of some ISs (*n*=21) was highly conserved between the six isolates of this study, and those conserved ISs were found in several copies in different genomic regions [>99 % sequence similarity, 100 % coverage including IRs, transposase(s), and cargo gene(s) when applicable]. These highly conserved interspecific ISs (IS*3*, IS*6*, IS*256*, IS*982* and IS*1182* families – elements IS*1485*, IS*1216*, IS*256*, IS*Efa13*, ISE*fm1* and IS*1182*, respectively) belong to 5 families out of the 11 and 12 families found in *E. faecalis* and *E. faecium *, respectively. To investigate whether any of these highly conserved ISs had been transferred between the two different species through IS-mediated homologous recombination events, we extended the sequence similarity analysis to the regions flanking these highly preserved ISs by up to 4000 bp upstream and downstream from matched ISs between species. Our analysis identified two ~7000 bp regions sharing >99.9 % sequence similarity with 100 % coverage between replicons from different species and bounded by IS elements (Fig. 3b, c). However, these two putative recombination events could have occurred between the two species at any time in their recent history and not necessarily during the specific co-infection described here, as these two species are regular inhabitants of the gastrointestinal tract.

The putative IS-mediated recombination event depicted in Fig. 3b was localized in pUMASEL1 and pUAMSEF4 plasmids from *E. faecalis* and *E. faecium* isolates, respectively. Further investigation of this putative recombination event showed a high resemblance, in terms of gene content, to a Tn5405-like element, mainly in *E. faecium* isolates. The Tn5405 element is a ~12 kb composite transposon that was previously identified in methicillin-resistant staphylococci [73] and later described in *E. faecium* species [74]. The gene clusters identified in our isolates contained the inducible by erythromycin *ermB* gene, two aminoglycoside ARDs (*aad*(6) and *aph*(3′)-*III*a), and the streptothricin resistance determinant gene *sat-4* (previously described linkage *aad(6)-sat4-aph(3′)-IIIa*) [72]. The genes in the Tn5405 element are usually flanked by inverted repeats of two IS*1182* elements. In our isolates, only one IS*1182* was preserved inside the region, while the entire area was bounded by IS*6* family elements (specifically IS*1216*) on one side and by Tn3 family transposases (TnBth1 in case of *E. faecalis* and Tn4430 for *E. faecium* isolates) on the other side, which did not share sequence similarity (Fig. 3b).

Immediately downstream of the region described above in pUAMSEL1 we found a second region of 7032 bp that was shared by all six isolates and was located in the pUAMSEF1a plasmid in *E. faecium* strains (Fig. 3c). No ARDs were identified in this region, which mainly harboured a set of genes encoding hypothetical proteins along with a recombinase, a BRCT domain-containing protein (usually belonging to proteins involved in cell cycle checkpoint functions that respond to DNA damage), and a transcriptional regulator. These regions were bounded in both species by exactly the same elements as those found in the previously described event: two identically oriented IS*6* family elements (IS*1216*) and a Tn3 family transposase in one of the flanks in *E. faecium* isolates and one IS*6* family (IS*1216*) and a Tn3 family transposase in the case of *E. faecalis* isolates. Analyses of these 2 regions using blastn with the NCBI nr/nt database (keeping only those genomes that harboured the entire region with identical flanking sequences and up to 70 internal mismatches) showed that these 2 regions are more prevalent among strains from *E. faecium* species, although they were found in a small number of *E. faecalis* strains (*n*=12 in the case of the first described region and *n*=16 in case of the second described region, without considering the isolates from this study) (Table S5). Multiple genome alignments of all the replicons in which these two regions were found indicate that only the regions described in our isolates (shown in Fig. 3b, c and bounded by ISs) were preserved in all replicons analysed (Figs S6 and S7).

### Novel arrangement of IS elements in Tn1546-like transposon in *E. faecalis* isolates likely implicated in a genotype–phenotype discrepancy

**Fig. 4.**
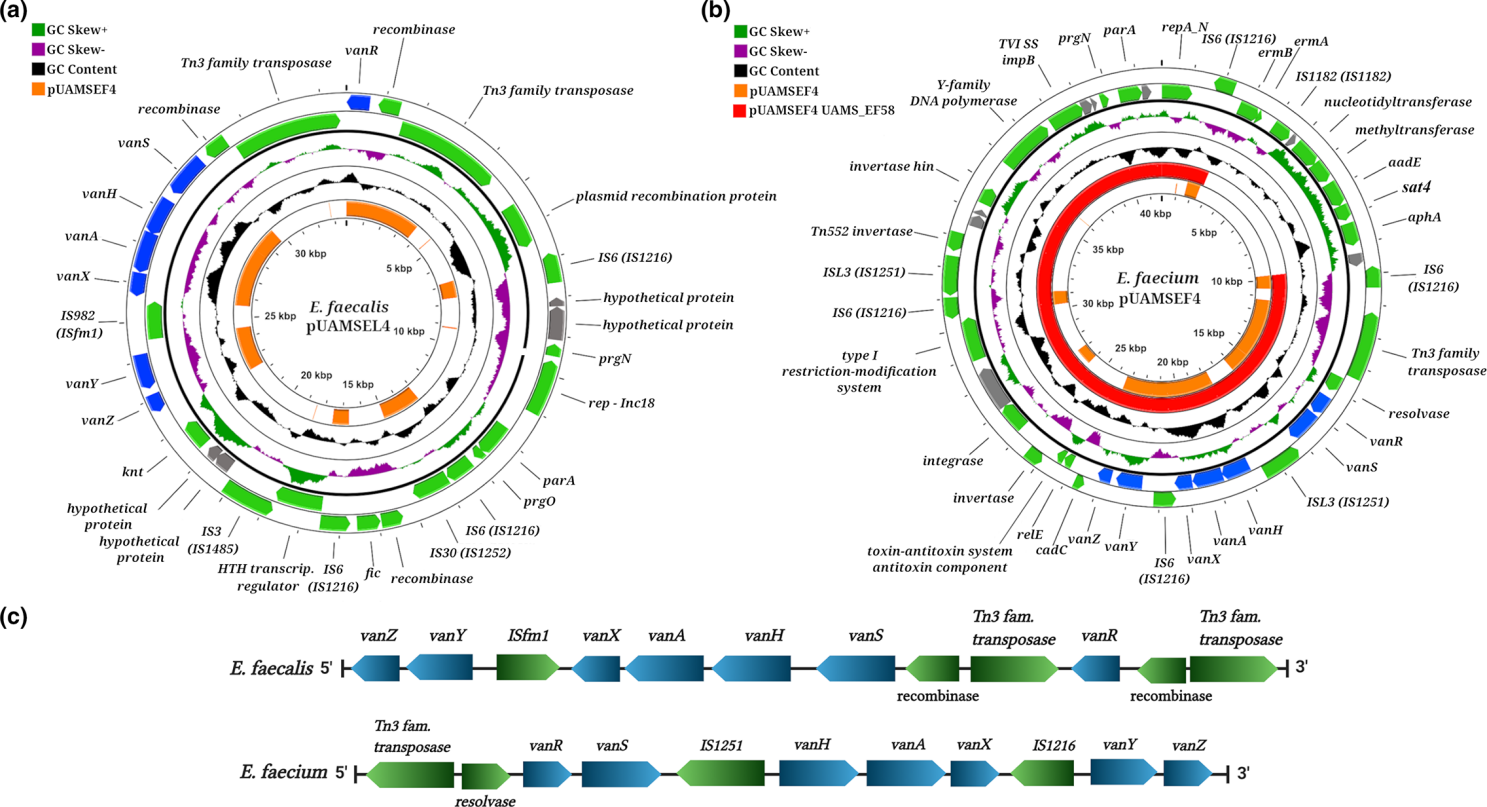
(a) Representation of the circularized vancomycin plasmid pUAMSEL4 from *E. faecalis *. The orange inner ring represents blastn alignment against pUAMSEF4 from *E. faecium* UAMS_EF55. The width of the orange bar represents the percentage of sequence similarity (full width implies values closer to 100 % sequence similarity). Genes annotated as hypothetical proteins are coloured in grey. (**b)** Representation of the circularized vancomycin plasmid pUAMSEF4 from *E. faecium *. The orange inner ring represents blastn alignment against pUAMSEL4 from *E. faecalis *. The red inner ring represents blastn alignment against pUAMSEF4 from UAMS_EF58 isolate. (**c)** Representation of *vanA* clusters and arrangements of ISs in *E. faecalis* and *E. faecium* isolates. Panels (a) and (b) were created using CGview server (version beta) and (c) was created using BioRender.

All of our *E. faecalis* isolates presented vancomycin MIC values of approximately 2 µg ml^−1^, indicating that they were vancomycin-sensitive (Table S1). However, all of these isolates also contained plasmids with complete *vanA* gene clusters known to confer vancomycin resistance, indicating a genotype–phenotype discrepancy (Fig. 4). Restoration of the vancomycin-resistant phenotype in enterococcal strains harbouring a ‘silent’ copy of vancomycin resistance genes through different molecular mechanisms has been described before [75].

A comparison of the vancomycin plasmids (pUAMSEL4 and pUAMSEF4) between the isolates from the two species from this study showed that *vanA*-containing plasmids differed in size, genetic content and type of plasmid replication protein (*rep*) gene. However, the genetic sequences of all seven genes in the *vanA* cluster (*vanR*, *vanS*, *vanH*, *vanA*, *vanX*, *vanY*, and *vanZ*) were highly conserved when aligned between isolates from both species (inner orange ring, Fig. 4a, b).

Plasmid pUAMSEL4 (carrying *vanA* gene cluster) from *E. faecalis* belongs to the incompatibility (Inc) 18-like family [76], which is a broad host range of conjugative plasmids that, under laboratory conditions, have been transferred successfully to a wide variety of microbes, such as lactococci [77], streptococci [78] and staphylococci [79, 80]. On the other hand, the *E. faecium vanA* plasmid pUAMSEF4 was classified as belonging to the RepA_N family, which has a narrow host range [81]. All *E. faecalis vanA* replicons were nearly identical, but we observed a significant difference, in terms of content and size, in the *vanA* plasmid from the last collected *E. faecium* isolate (UAMS_EF58) (Figs 4 and S2A). The decrease in size of the UAMS_EF58 *vanA* plasmid was due to an IS-mediated deletion of a region located between the two identically oriented IS*6* elements (specifically, IS*1216*) located in the first quarter of pUAMSEF4 (red inner ring in Figs 4b and S8). Thus, pUAMSEF4 from UAMS_EF58 consisted of a replicon ~7000 bp shorter than the *vanA* plasmids found in previously collected *E. faecium* isolates UAMS_EF55 and UAMS_EF57. In this excision event, both identically oriented IS*6* elements were retained in the *van* plasmid from UAMS_EF58, but none of the genes bounded by them were retained (Figs 4b and S8). The cluster of genes absent in pUAMSEF4 from UAMS_EF58 contained several ARDs that code for resistance to aminoglycosides (*aadE, aphA*), nucleosides (*sat-4*), and macrolides, lincosamides and streptogramins (*ermAB*) (Fig. 4b, Table S3).

Although the sequence of genes in the *vanA* cluster was highly preserved among our six isolates, *vanA* gene clusters from both species presented different organizations of the Tn1546-like element. The Tn1546-like transposon in pUAMSEF4 from *E. faecium* isolates (Fig. 4c) showed a genetic organization that is highly preserved among hospital-acquired vancomycin-resistant *E. faecium* isolates [82]. However, we observed an unusual genetic organization of the Tn1546-like transposon in our *E. faecalis* isolates; this peculiar organization of ISs in the *vanA* cluster was not found in any other strains when the entire region was queried in the NCBI public database (Fig. S9).

## Discussion

Enterococci are widely distributed across many different niches, but their primary habitat is the gastrointestinal tract [83]. Among enterococci, *E. faecalis* is the most common cause of opportunistic infection, but *E. faecium* is intrinsically more resistant to antibiotics. More than half of the nosocomial *E. faecium* isolates in the USA have expressed resistance to ampicillin and vancomycin and high-level resistance to aminoglycosides [84].

While the important role that ISs and other mobile genetic elements have in the persistence and dissemination of ARDs has been already established in previous works on enterococci [85, 86], there is a lack of information on the association between ISs and ARDs and other aspects regarding the nature, maintenance and mechanisms of transmission of ISs in bacterial genomes. In this work, we present two bacterial species, sharing the same environment (patient with bacteraemia), with a very similar content of IS families but drastically different copy numbers and arrangements of ISs throughout their replicons. *E. faecium* genomes harboured a strikingly high number of ISs (Fig. 1b) and showed a higher level of IS-mediated activity (4 days passed between the first and last sample being collected). Considering the first isolate collected for each species as the reference (UAMS_EL53 for *E. faecalis* and UAMS_EF55 for *E. faecium *), the only significant intraspecific genomic differences found between the isolates of the study were the IS-mediated events described in this paper, highlighting the significant role ISs and other transposable elements play as elements that generate rapid genomic adaptations (Fig. S1) [24].

We identified 11 different IS families in the *E. faecalis* isolates that were also present in the *E. faecium* isolates. An additional IS family was identified in the *E. faecium* isolates. The presence of a high number of shared IS families with highly conserved DNA sequences between strains from both species suggests that these elements could have been spread through horizontal gene transfer. Most importantly, IS propagation generates multiple homologous sequences scattered throughout the bacterial genomes, which are substrates for homologous recombination [63], paving the way for a successful transmission of the antibiotic resistance determinants and virulence factors that frequently accompany these elements in *E. faecalis* and *E. faecium* genomes. In agreement with previous reports, our six isolates showed a strong correlation between the IS elements identified and the annotated ARDs, in the sense that the majority of ARDs had at least one IS element in their surrounding regions, suggesting that IS elements might have a role in the transfer of these ARDs and other virulence factors [22, 60].

Although mutations produced by ISs are generally deleterious [29, 58] and bacterial genomes possess mechanisms that suppress transposition [29, 87, 88], ISs can promote adaptive evolution by gene inactivation, triggering genomic deletions and modulating gene expression, which can be beneficial in the transition between free-living to parasitic lifestyles [24, 89]. Thus, a high level of transposition activity has been understood as the mechanism of adaptation to stressful and dynamic environmental conditions that can generate sufficient genetic variation to increase fitness [89]. Therefore, the strikingly high number of ISs found in the *E. faecium* isolates from this study in comparison with those from the set of complete *E. faecium* obtained from public repositories (Fig. 1) could be a consequence of the high rates of replicative transposition in response to the stressful and competitive conditions of the bloodstream environment, which is not the natural habitat of enterococci. However, it is worth mentioning that the high number of ISs found in our isolates compared to previous reports [90] could be partially due to our use of high-quality, complete genomes, considering that draft assemblies are typically highly fragmented and their contigs are usually broken at IS locations [91] due to the repetitive nature of IS elements [22, 60, 63].

It has been documented that elements from the IS*6* family with flanking ISs in direct repeat are able to form transposons that resemble compound transposons through a mechanism that involves the formation of cointegrates, also known as pseudo-compound transposons [92–94], and also via circular translocatable units [94, 95]. Potential mechanisms of circular molecular transposition (translocatable units) mediated by elements from IS*6* and IS*26* families have been described [94, 96]. Transposon-derived circular forms have been documented in Gram-negative bacteria such as *Acinetobacter baumannii* [97] and *Escherichia coli* [98]. In this work, we provide computational evidence of the formation of a complete conjugative plasmid (pUAMSEF3) with all of the necessary machinery and functional elements for its own replication and transference (Fig. 2b); this plasmid was the product of IS-mediated excision from a larger plasmid (pUAMSEF1a) (Fig. 2a), supporting the key role of translocatable units in disseminating genetic material as autonomous entities.

We also observed multiple putative pseudo-compound transposons when analysing the genomes of our three *E. faecium* isolates (Figs 1a and 3a). However, this type of structure had never been described before, to the best of our knowledge, for members of the IS*L3* family. The structure observed in our *E. faecium* isolates is composed of two directly oriented IS*L3* elements surrounding *liaSR* with DAP-resistant associated mutations and surrounding genes. Further analysis including all complete, publicly available *E. faecium* genomes confirmed that the novel structure is specific to *E. faecium* genomes with DAP resistance mutations in *liaSR* genes. Our findings suggest a new paradigm for the investigation of the mechanisms of DAP resistance and their dissemination in *E. faecium* species, in the sense that the development of a DAP-resistant phenotype might not be produced by sequential co-mutations in *liaSR* genes but through IS-mediated transmission of the entire set of genes already carrying these mutations. However, we found no duplication of this cluster in our *E. faecium* isolates or in the other 33 complete *E. faecium* genomes harbouring *liaSR* mutations. In the same vein, the upstream and downstream genetic neighbourhood of the IS*L3* bounded structure was shared between all complete isolates queried, therefore homologous recombination might be a more plausible mechanism of propagation for these mutations in *liaSR* genes than IS-mediated transposition. Homologous recombination mediated by ISs between related strains has been previously documented in other works [99–101]. Further detailed examination of the IS duplications at target sites and experimental assays should be carried out to determine whether this IS*L3* bounded structure, observed in all *E. faecium* genomes carrying amino acid substitutions in LiaR (W73C) and LiaS (T120A), is crucial in their dissemination.

Our *E. faecalis* isolates harboured a complete *vanA* cluster but presented MIC values ≤2 for vancomycin, indicating a genotype–phenotype discrepancy. It has previously been reported that the *vanA* cluster (*vanRSHAXYZ*) is prone to IS-mediated alterations that can affect the resistance phenotype of a strain, even in the case of a *vanA*-positive genotype [102, 103]. Among the most widely documented effects of IS transposition is gene inactivation [24, 104], and the novel arrangement of ISs found in the *vanA* cluster of our three *E. faecalis* isolates might explain the observed genotype–phenotype discrepancy. Similar events have been documented in other species [102, 103]. A particularly interesting case was described by Siversten and colleagues [103] in which initially vancomycin-susceptible *E. faecium* isolates were able to survive for several days during subclinical breakpoint exposure to glycopeptides until they developed the resistant phenotype after the excision of one IS element present in the *vanA* gene cluster. We speculate that this novel arrangement of IS elements in the Tn1546-like transposon in *E. faecalis* isolates with a *vanA*-positive genotype from our study might be the cause of the ‘silenced’ phenotype observed when these isolates were collected.

In summary, while our computational results are all biological observations that need to be explored experimentally, our results substantiate that IS-mediated events differ between the two species, *E. faecium* and *E. faecalis *, from the same environment under complex selective forces occasioned by intensive antibiotic treatment. In addition, some of these IS-mediated reorganizations are noticeable even between isolates collected from the same subject in a short period of time. Our results highlight the major role of ISs and other transposable elements in the rapid genomic adaptation and response of enterococci to clinically relevant stresses.

## Supplementary Data

Supplementary material 1

## References

[1] Lewis K, Caboni M (2017). The making of a pathogen. Cell Host Microbe.

[2] Ch’ng J-H, Chong KKL, Lam LN, Wong JJ, Kline KA (2019). Biofilm-associated infection by enterococci. Nat Rev Microbiol.

[3] Price VJ, McBride SW, Hullahalli K, Chatterjee A, Duerkop BA (2019). *Enterococcus faecalis* CRISPR-Cas is a robust barrier to conjugative antibiotic resistance dissemination in the murine intestine. mSphere.

[4] Fiore E, Van Tyne D, Gilmore MS, Fischetti VA, Novick RP (2019). Pathogenicity of *Enterococci*. Microbiol Spectr.

[5] Molina L, Udaondo Z, Duque E, Fernández M, Bernal P (2016). Specific gene loci of clinical *Pseudomonas putida* isolates. PLOS ONE.

[6] Faron ML, Ledeboer NA, Buchan BW (2016). Resistance mechanisms, epidemiology, and approaches to screening for vancomycin-resistant *Enterococcus* in the health care setting. J Clin Microbiol.

[7] Rios R, Reyes J, Carvajal LP, Rincon S, Panesso D (2020). Genomic epidemiology of vancomycin-resistant *Enterococcus faecium* (VREfm) in Latin America: revisiting the global VRE population structure. Sci Rep.

[8] van Hal SJ, Ip CLC, Ansari MA, Wilson DJ, Espedido BA (2016). Evolutionary dynamics of *Enterococcus faecium* reveals complex genomic relationships between isolates with independent emergence of vancomycin resistance. Microb Genom.

[9] van Hal SJ, Willems RJL, Gouliouris T, Ballard SA, Coque TM (2021). The global dissemination of hospital clones of *Enterococcus faecium*. Genome Med.

[10] van Hal SJ, Willems RJL, Gouliouris T, Ballard SA, Coque TM (2022). The interplay between community and hospital *Enterococcus faecium* clones within health-care settings: a genomic analysis. Lancet Microbe.

[11] Lebreton F, Manson AL, Saavedra JT, Straub TJ, Earl AM (2017). Tracing the *Enterococci* from paleozoic origins to the Hospital. Cell.

[12] Pöntinen AK, Top J, Arredondo-Alonso S, Tonkin-Hill G, Freitas AR (2021). Apparent nosocomial adaptation of *Enterococcus faecalis* predates the modern hospital era. Nat Commun.

[13] Top J, Arredondo-Alonso S, Schürch AC, Puranen S, Pesonen M (2020). Genomic rearrangements uncovered by genome-wide co-evolution analysis of a major nosocomial pathogen, *Enterococcus faecium*. Microb Genom.

[14] Igbinosa EO, Beshiru A (2019). Antimicrobial resistance, virulence determinants, and biofilm formation of *Enterococcus* species from ready-to-eat seafood. Front Microbiol.

[15] Arredondo-Alonso S, Top J, McNally A, Puranen S, Pesonen M (2020). Plasmids shaped the recent emergence of the major nosocomial pathogen *Enterococcus faecium*. mBio.

[16] Bayjanov JR, Baan J, Rogers MRC, Troelstra A, Willems RJL (2019). *Enterococcus faecium* genome dynamics during long-term asymptomatic patient gut colonization. Microb Genom.

[17] Freitas AR, Tedim AP, Francia MV, Jensen LB, Novais C (2016). Multilevel population genetic analysis of vanA and vanB *Enterococcus faecium* causing nosocomial outbreaks in 27 countries (1986-2012). J Antimicrob Chemother.

[18] Orlek A, Stoesser N, Anjum MF, Doumith M, Ellington MJ (2017). Plasmid classification in an Era of whole-genome sequencing: application in studies of antibiotic resistance epidemiology. Front Microbiol.

[19] Cao MD, Nguyen SH, Ganesamoorthy D, Elliott AG, Cooper MA (2017). Scaffolding and completing genome assemblies in real-time with nanopore sequencing. Nat Commun.

[20] Rezaei Javan R, Ramos-Sevillano E, Akter A, Brown J, Brueggemann AB (2019). Prophages and satellite prophages are widespread in *Streptococcus* and may play a role in *Pneumococcal pathogenesis*. Nat Commun.

[21] Siguier P, Gourbeyre E, Chandler M (2014). Bacterial insertion sequences: their genomic impact and diversity. FEMS Microbiol Rev.

[22] Partridge SR, Kwong SM, Firth N, Jensen SO (2018). Mobile genetic elements associated with antimicrobial resistance. Clin Microbiol Rev.

[23] Hickman AB, Dyda F (2015). Mechanisms of DNA Transposition. In: Mobile DNA III. https://onlinelibrary.wiley.com/doi/abs/10.1128/9781555819217.ch25.

[24] Vandecraen J, Chandler M, Aertsen A, Van Houdt R (2017). The impact of insertion sequences on bacterial genome plasticity and adaptability. Crit Rev Microbiol.

[25] Berg DE, Berg CM, Sasakawa C (1984). Bacterial transposon Tn5: evolutionary inferences. Mol Biol Evol.

[26] Alton NK, Vapnek D (1979). Nucleotide sequence analysis of the chloramphenicol resistance transposon Tn9. Nature.

[27] Foster TJ, Davis MA, Roberts DE, Takeshita K, Kleckner N (1981). Genetic organization of transposon Tn10. Cell.

[28] Prudhomme M, Turlan C, Claverys J-P, Chandler M (2002). Diversity of Tn4001 transposition products: the flanking IS256 elements can form tandem dimers and IS circles. J Bacteriol.

[29] Wu Y, Aandahl RZ, Tanaka MM (2015). Dynamics of bacterial insertion sequences: can transposition bursts help the elements persist?. BMC Evol Biol.

[30] Clinical and Laboratory Standards Institute (2019). Performance Standards for Antimicrobial Susceptibility Testing.

[31] Udaondo Z, Wongsurawat T, Jenjaroenpun P, Anderson C, Lopez J (2019). Draft genome sequences of 48 vancomycin-Resistant *Enterococcus faecium* strains isolated from inpatients with bacteremia and urinary tract infection. Microbiol Resour Announc.

[32] Udaondo Z, Jenjaroenpun P, Wongsurawat T, Meyers E, Anderson C (2020). Two cases of vancomycin-resistant *Enterococcus faecium* bacteremia with development of daptomycin-resistant phenotype and its detection using Oxford nanopore equencing. Open Forum Infect Dis.

[33] Chen S, Zhou Y, Chen Y, Gu J (2018). fastp: an ultra-fast all-in-one FASTQ preprocessor. Bioinformatics.

[34] Bolger AM, Lohse M, Usadel B (2014). Trimmomatic: a flexible trimmer for Illumina sequence data. Bioinformatics.

[35] Andrews S. FASTQC (2010). A quality control tool for high throughput sequence data. Internet.

[36] Wick RR, Judd LM, Holt KE (2019). Performance of neural network basecalling tools for Oxford Nanopore sequencing. Genome Biol.

[37] De Coster W, D’Hert S, Schultz DT, Cruts M, Van Broeckhoven C (2018). NanoPack: visualizing and processing long-read sequencing data. Bioinformatics.

[38] Wick RR, Judd LM, Gorrie CL, Holt KE (2017). Unicycler: resolving bacterial genome assemblies from short and long sequencing reads. PLOS Comput Biol.

[39] Koren S, Walenz BP, Berlin K, Miller JR, Bergman NH (2017). Canu: scalable and accurate long-read assembly via adaptive *k*-mer weighting and repeat separation. Genome Res.

[40] Kolmogorov M, Yuan J, Lin Y, Pevzner PA (2019). Assembly of long, error-prone reads using repeat graphs. Nat Biotechnol.

[41] Vaser R, Sović I, Nagarajan N, Šikić M (2017). Fast and accurate de novo genome assembly from long uncorrected reads. Genome Res.

[42] Walker BJ, Abeel T, Shea T, Priest M, Abouelliel A (2014). Pilon: an integrated tool for comprehensive microbial variant detection and genome assembly improvement. PLOS ONE.

[43] Seemann T (2014). Prokka: rapid prokaryotic genome annotation. Bioinformatics.

[44] Alcock BP, Raphenya AR, Lau TTY, Tsang KK, Bouchard M (2020). CARD 2020: antibiotic resistome surveillance with the comprehensive antibiotic resistance database. Nucleic Acids Res.

[45] Xie Z, Tang H (2017). ISEScan: automated identification of insertion sequence elements in prokaryotic genomes. Bioinformatics.

[46] Siguier P, Perochon J, Lestrade L, Mahillon J, Chandler M (2006). ISfinder: the reference centre for bacterial insertion sequences. Nucleic Acids Res.

[47] Carattoli A, Hasman H (2020). Plasmidfinder and in silico pMLST: identification and typing of plasmid replicons in whole-genome sequencing (WGS). Methods Mol Biol.

[48] Grant JR, Stothard P (2008). The CGView Server: a comparative genomics tool for circular genomes. Nucleic Acids Res.

[49] Jolley KA, Maiden MCJ (2010). BIGSdb: Scalable analysis of bacterial genome variation at the population level. BMC Bioinformatics.

[50] Altschul SF, Gish W, Miller W, Myers EW, Lipman DJ (1990). Basic local alignment search tool. J Mol Biol.

[51] Ondov BD, Treangen TJ, Melsted P, Mallonee AB, Bergman NH (2016). Mash: fast genome and metagenome distance estimation using MinHash. Genome Biol.

[52] Abram K, Udaondo Z, Bleker C, Wanchai V, Wassenaar TM (2021). Mash-based analyses of *Escherichia coli* genomes reveal 14 distinct phylogroups. Commun Biol.

[53] Gilchrist CLM, Chooi Y-H (2021). Clinker & clustermap.js: automatic generation of gene cluster comparison figures. Bioinformatics.

[54] Lopez-Delisle L, Rabbani L, Wolff J, Bhardwaj V, Backofen R (2021). pyGenomeTracks: reproducible plots for multivariate genomic datasets. Bioinformatics.

[55] Li H (2018). Minimap2: pairwise alignment for nucleotide sequences. Bioinformatics.

[56] Carver TJ, Rutherford KM, Berriman M, Rajandream M-A, Barrell BG (2005). ACT: the artemis comparison tool. Bioinformatics.

[57] Angiuoli SV, Salzberg SL (2011). Mugsy: fast multiple alignment of closely related whole genomes. Bioinformatics.

[58] Nzabarushimana E, Tang H (2018). Insertion sequence elements-mediated structural variations in bacterial genomes. Mob DNA.

[59] Blázquez J, Couce A, Rodríguez-Beltrán J, Rodríguez-Rojas A (2012). Antimicrobials as promoters of genetic variation. Curr Opin Microbiol.

[60] Razavi M, Kristiansson E, Flach C-F, Larsson DGJ (2020). The association between insertion sequences and antibiotic resistance genes. mSphere.

[61] Ross K, Varani AM, Snesrud E, Huang H, Alvarenga DO (2021). TnCentral: a prokaryotic transposable element database and web portal for transposon analysis. mBio.

[62] Kadlec K, Schwarz S (2010). Identification of the novel dfrK-carrying transposon Tn559 in a porcine methicillin-susceptible *Staphylococcus aureus* ST398 strain. Antimicrob Agents Chemother.

[63] Lee H, Doak TG, Popodi E, Foster PL, Tang H (2016). Insertion sequence-caused large-scale rearrangements in the genome of *Escherichia coli*. Nucleic Acids Res.

[64] Ward LJH, Brown JCS, Davey GP (1996). Identification and sequence analysis of IS1297, an ISS1-like insertion sequence in a *Leuconostoc strain*. Gene.

[65] Arias CA, Panesso D, McGrath DM, Qin X, Mojica MF (2011). Genetic basis for in vivo daptomycin resistance in enterococci. N Engl J Med.

[66] Campeau SA, Schuetz AN, Kohner P, Arias CA, Hemarajata P (2018). Variability of daptomycin MIC values for *Enterococcus faecium* when measured by reference broth microdilution and gradient diffusion tests. Antimicrob Agents Chemother.

[67] Mericl AN, Friesen JA (2012). Comparative kinetic analysis of glycerol 3-phosphate cytidylyltransferase from *Enterococcus faecalis* and *Listeria monocytogenes*. Med Sci Monit.

[68] Han W, Wu B, Li L, Zhao G, Woodward R (2012). Defining function of lipopolysaccharide O-antigen ligase WaaL using chemoenzymatically synthesized substrates. J Biol Chem.

[69] Cui G, Wang J, Qi X, Su J (2018). Transcription elongation factor GreA plays a key role in cellular nvasion and virulence of *Francisella tularensis* subsp. novicida. Sci Rep.

[70] Munita JM, Mishra NN, Alvarez D, Tran TT, Diaz L (2014). Failure of high-dose daptomycin for bacteremia caused by daptomycin-susceptible *Enterococcus faecium* harboring LiaSR substitutions. Clin Infect Dis.

[71] Arias CA, Murray BE (2012). The rise of the *Enterococcus*: beyond vancomycin resistance. Nat Rev Microbiol.

[72] Kebriaei R, Rice SA, Singh KV, Stamper KC, Dinh AQ (2018). Influence of inoculum effect on the efficacy of daptomycin monotherapy and in combination with β-Lactams against daptomycin-susceptible *Enterococcus faecium* harboring LiaSR Substitutions. Antimicrob Agents Chemother.

[73] Derbise A, de Cespedes G, el Solh N (1997). Nucleotide sequence of the *Staphylococcus aureus* transposon, Tn5405, carrying aminoglycosides resistance genes. J Basic Microbiol.

[74] Werner G, Hildebrandt B, Witte W (2003). Linkage of erm(B) and aadE-sat4-aphA-3 in multiple-resistant *Enterococcus faecium* isolates of different ecological origins. Microb Drug Resist.

[75] Thaker MN, Kalan L, Waglechner N, Eshaghi A, Patel SN (2015). Vancomycin-variable enterococci can give rise to constitutive resistance during antibiotic therapy. Antimicrob Agents Chemother.

[76] Kohler V, Vaishampayan A, Grohmann E (2018). Broad-host-range Inc18 plasmids: Occurrence, spread and transfer mechanisms. Plasmid.

[77] Langella P, Chopin A (1989). Conjugal transfer of plasmid pIP501 from *Lactococcus lactis* to *Lactobacillus delbrückii* subsp. *bulgaricus* and *Lactobacillus helveticus*. FEMS Microbiol Lett.

[78] Thompson JK, Collins MA (1988). Evidence for the conjugal transfer of the broad host range plasmid pIP501 into strains of *Lactobacillus helveticus*. J Appl Bacteriol.

[79] Schaberg DR, Clewell DB, Glatzer L (1982). Conjugative transfer of R-plasmids from *Streptococcus faecalis* to *Staphylococcus aureus*. Antimicrob Agents Chemother.

[80] Zhu W, Clark N, Patel JB (2013). pSK41-like plasmid is necessary for Inc18-like vanA plasmid transfer from *Enterococcus faecalis* to *Staphylococcus aureus in vitro*. Antimicrob Agents Chemother.

[81] Mikalsen T, Pedersen T, Willems R, Coque TM, Werner G (2015). Investigating the mobilome in clinically important lineages of *Enterococcus faecium* and *Enterococcus faecalis*. BMC Genomics.

[82] Wang G, Yu F, Lin H, Murugesan K, Huang W (2018). Evolution and mutations predisposing to daptomycin resistance in vancomycin-resistant *Enterococcus faecium* ST736 strains. PLoS ONE.

[83] He Q, Hou Q, Wang Y, Li J, Li W (2018). Comparative genomic analysis of *Enterococcus faecalis*: insights into their environmental adaptations. BMC Genomics.

[84] O’Driscoll T, Crank CW (2015). Vancomycin-resistant *Enterococcal* infections: epidemiology, clinical manifestations, and optimal management. Infect Drug Resist.

[85] Hegstad K, Mikalsen T, Coque TM, Werner G, Sundsfjord A (2010). Mobile genetic elements and their contribution to the emergence of antimicrobial resistant *Enterococcus faecalis* and *Enterococcus faecium*. Clin Microbiol Infect.

[86] Shankar N, Baghdayan AS, Gilmore MS (2002). Modulation of virulence within a pathogenicity island in vancomycin-resistant *Enterococcus faecalis*. Nature.

[87] Foster PL (2007). Stress-induced mutagenesis in bacteria. Crit Rev Biochem Mol Biol.

[88] Hua-Van A, Le Rouzic A, Boutin TS, Filée J, Capy P (2011). The struggle for life of the genome’s selfish architects. Biol Direct.

[89] Consuegra J, Gaffé J, Lenski RE, Hindré T, Barrick JE (2021). Insertion-sequence-mediated mutations both promote and constrain evolvability during a long-term experiment with bacteria. Nat Commun.

[90] Clewell DB, Weaver KE, Dunny GM, Coque TM, Francia MV (2014). Enterococci: From Commensals to Leading Causes of Drug Resistant Infection.

[91] Sohn J, Nam J-W (2018). The present and future of *de novo* whole-genome assembly. Brief Bioinformatics.

[92] Harmer CJ, Pong CH, Hall RM (2020). Structures bounded by directly-oriented members of the IS26 family are pseudo-compound transposons. Plasmid.

[93] Galas DJ, Chandler M, Berg DE, Howe MM (1989). Mob DNA.

[94] Varani A, He S, Siguier P, Ross K, Chandler M (2021). The IS6 family, a clinically important group of insertion sequences including IS26. Mob DNA.

[95] Harmer CJ, Moran RA, Hall RM (2014). Movement of IS26-associated antibiotic resistance genes occurs via a translocatable unit that includes a single IS26 and preferentially inserts adjacent to another IS26. mBio.

[96] Harmer CJ, Hall RM (2016). IS26-Mediated formation of transposons carrying antibiotic resistance genes. mSphere.

[97] Karah N, Dwibedi CK, Sjöström K, Edquist P, Johansson A (2016). Novel aminoglycoside resistance transposons and transposon-derived circular forms detected in carbapenem-resistant acinetobacter baumannii clinical solates. Antimicrob Agents Chemother.

[98] Ghosh H, Doijad S, Bunk B, Falgenhauer L, Yao Y (2016). Detection of translocatable units in a blaCTX-M-15 extended-spectrum β-lactamase-producing ST131 *Escherichia coli* isolate using a hybrid sequencing approach. Int J Antimicrob Agents.

[99] Daveran-Mingot M-L, Campo N, Ritzenthaler P, Le Bourgeois P (1998). A natural large chromosomal inversion in *Lactococcus lactis* is mediated by homologous recombination between two insertion sequences. J Bacteriol.

[100] Zong Z, Partridge SR, Iredell JR (2010). ISEcp1-mediated transposition and homologous recombination can explain the context of bla(CTX-M-62) linked to qnrB2. Antimicrob Agents Chemother.

[101] Wang Z, Fu Y, Du X-D, Jiang H, Wang Y (2018). Potential transferability of mcr-3 via IS26-mediated homologous recombination in *Escherichia coli*. Emerg Microbes Infect.

[102] Gagnon S, Lévesque S, Lefebvre B, Bourgault A-M, Labbé A-C (2011). vanA-containing *Enterococcus faecium* susceptible to vancomycin and teicoplanin because of major nucleotide deletions in Tn1546. J Antimicrob Chemother.

[103] Sivertsen A, Pedersen T, Larssen KW, Bergh K, Rønning TG (2016). A silenced *vanA* gene cluster on a transferable plasmid caused an outbreak of vancomycin-variable *Enterococci*. Antimicrob Agents Chemother.

[104] Rojo-Bezares B, Estepa V, Cebollada R, de Toro M, Somalo S (2014). Carbapenem-resistant *Pseudomonas aeruginosa* strains from a Spanish hospital: characterization of metallo-beta-lactamases, porin OprD and integrons. Int J Med Microbiol.

