# Supplementary material for: Insertion sequences and other mobile elements associated with antibiotic resistance genes in Enterococcus isolates from an inpatient with prolonged bacteraemia
Source: Microb Genom. 2022 Aug 3;8(8):mgen000855. doi: 10.1099/mgen.0.000855 (PMC9484755; doi:10.1099/mgen.0.000855)
Supplement: Supplementary material 1 [file mgen-8-855-s001.pdf]

Insertion sequences and other mobile elements associated with antibiotic resistance genes in *Enterococcus* isolates from an inpatient with prolonged bacteremia.
SUPPLEMENTARY MATERIAL.

Supplementary Figure 1.A. Representation of the comparison of the chromosomic sequences of *E. faecalis* isolates UAMS_EL53, UAMS_EL54 and UAMS_EL56 using Artemis Comparison Tool (ACT) release 18.1.0 (1). Comparison files for ACT input were created using blastn pairwise alignments of the three chromosomes on the NCBI Blast website. Red and blue bars indicate regions of similarity with red bars indicating corresponding regions that are oriented similarly and blue bars indicating regions oriented in opposite directions.

A.

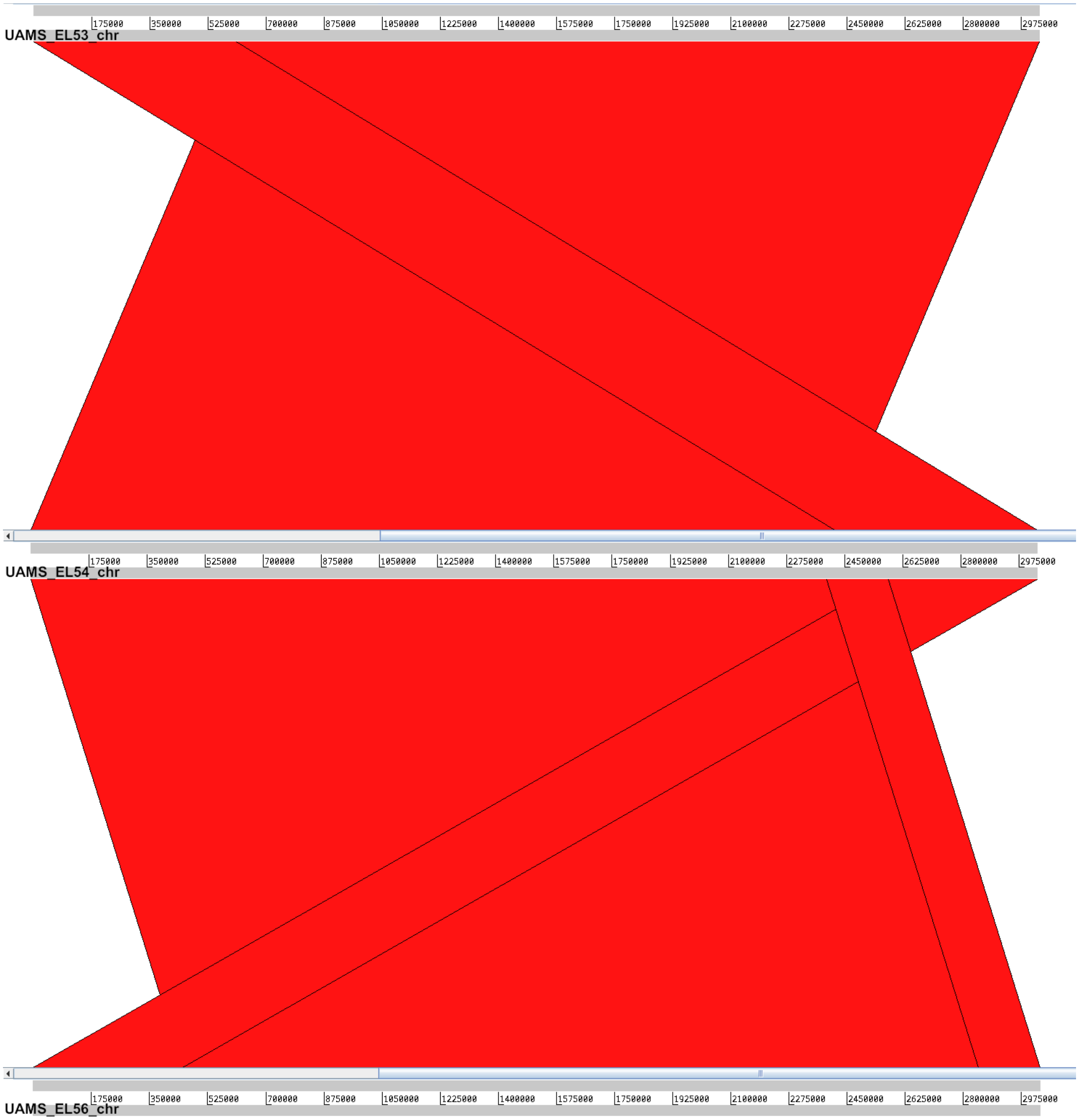

Supplementary Figure 1.B. Representation of the comparison of the chromosomic sequences of *E. faecium* isolates UAMS_EF55, UAMS_EF57 and UAMS_EF58 using Artemis Comparison Tool (ACT) release 18.1.0 (1). Comparison files for ACT input were created using blastn pairwise alignments of the three chromosomes on the NCBI Blast website. Red and blue bars indicate regions of similarity with red bars indicating corresponding regions that are oriented similarly and blue bars indicating regions oriented in opposite directions.

B.

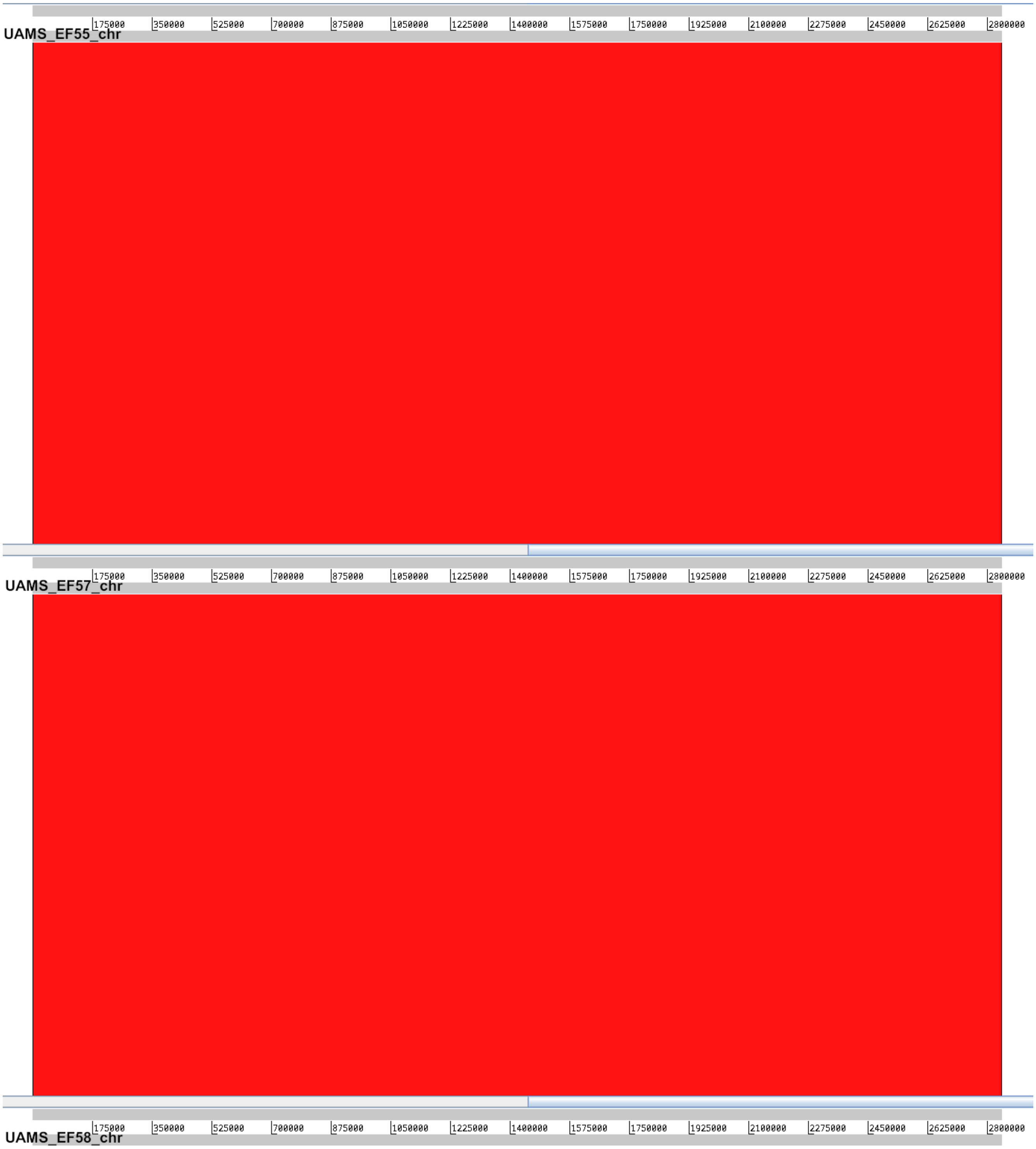

Supplementary Figure 1.C. Representation of the comparison of the plasmid sequence from plasmid pUAMSEL1 of *E. faecalis* isolates UAMS_EL53, UAMS_EL54 and UAMS_EL56 using Artemis Comparison Tool (ACT) release 18.1.0 (1). Comparison files for ACT input were created using blastn pairwise alignments of the three plasmids on the NCBI Blast website. Red and blue bars indicate regions of similarity with red bars indicating corresponding regions that are oriented similarly and blue bars indicating regions oriented in opposite directions.

C.

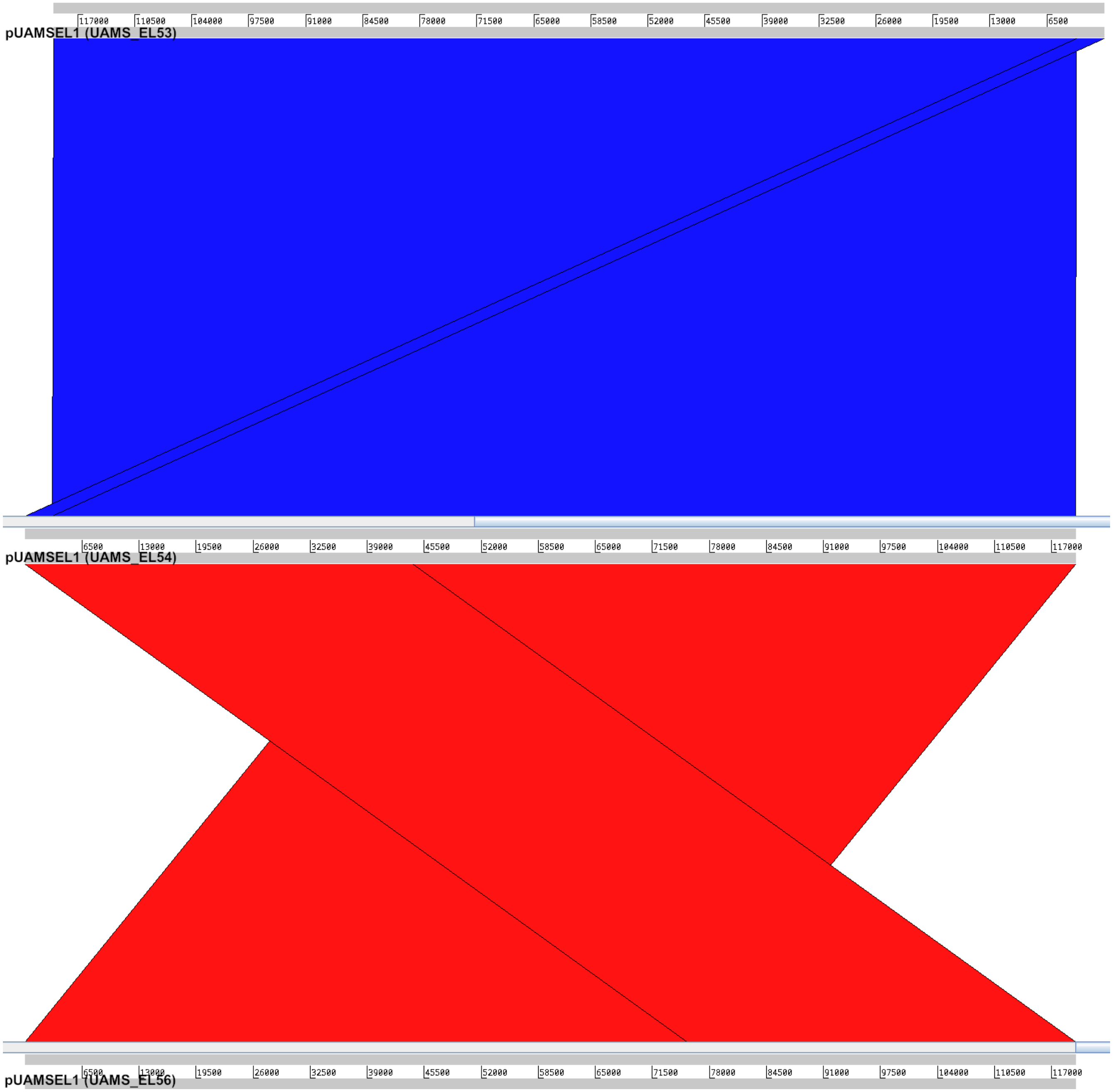

Supplementary Figure 1.D. Representation of the comparison of the plasmid sequence from plasmid pUAMSEL2 of *E. faecalis* isolates UAMS_EL53, UAMS_EL54 and UAMS_EL56 using Artemis Comparison Tool (ACT) release 18.1.0 (1). Comparison files for ACT input were created using blastn pairwise alignments of the three plasmids on the NCBI Blast website. Red and blue bars indicate regions of similarity with red bars indicating corresponding regions that are oriented similarly and blue bars indicating regions oriented in opposite directions.

D.

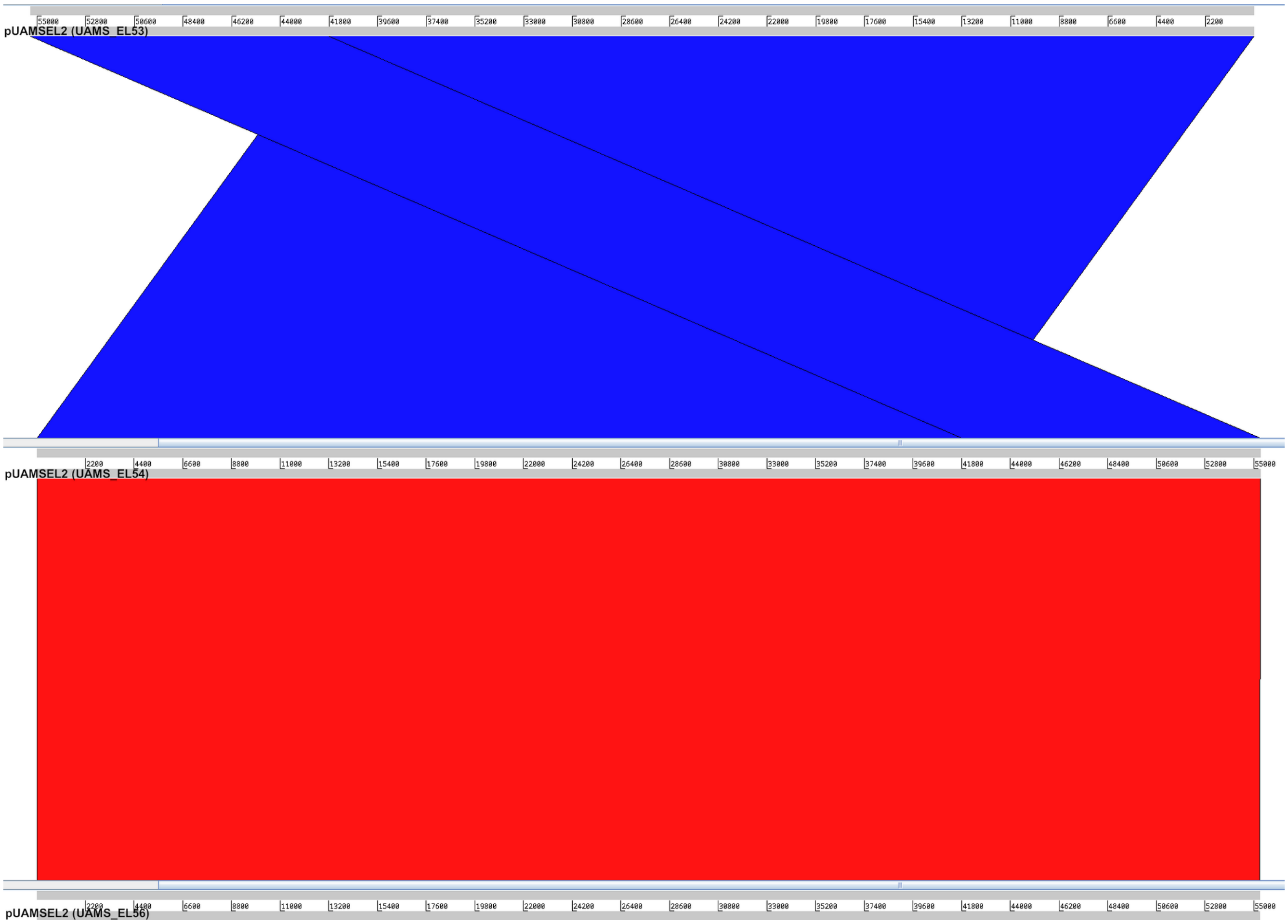

Supplementary Figure 1.E. Representation of the comparison of the plasmid sequence from plasmid pUAMSEL3 of *E. faecalis* isolates UAMS_EL53, UAMS_EL54 and UAMS_EL56 using Artemis Comparison Tool (ACT) release 18.1.0 (1). Comparison files for ACT input were created using blastn pairwise alignments of the three plasmids on the NCBI Blast website. Red and blue bars indicate regions of similarity with red bars indicating corresponding regions that are oriented similarly and blue bars indicating regions oriented in opposite directions.

E.

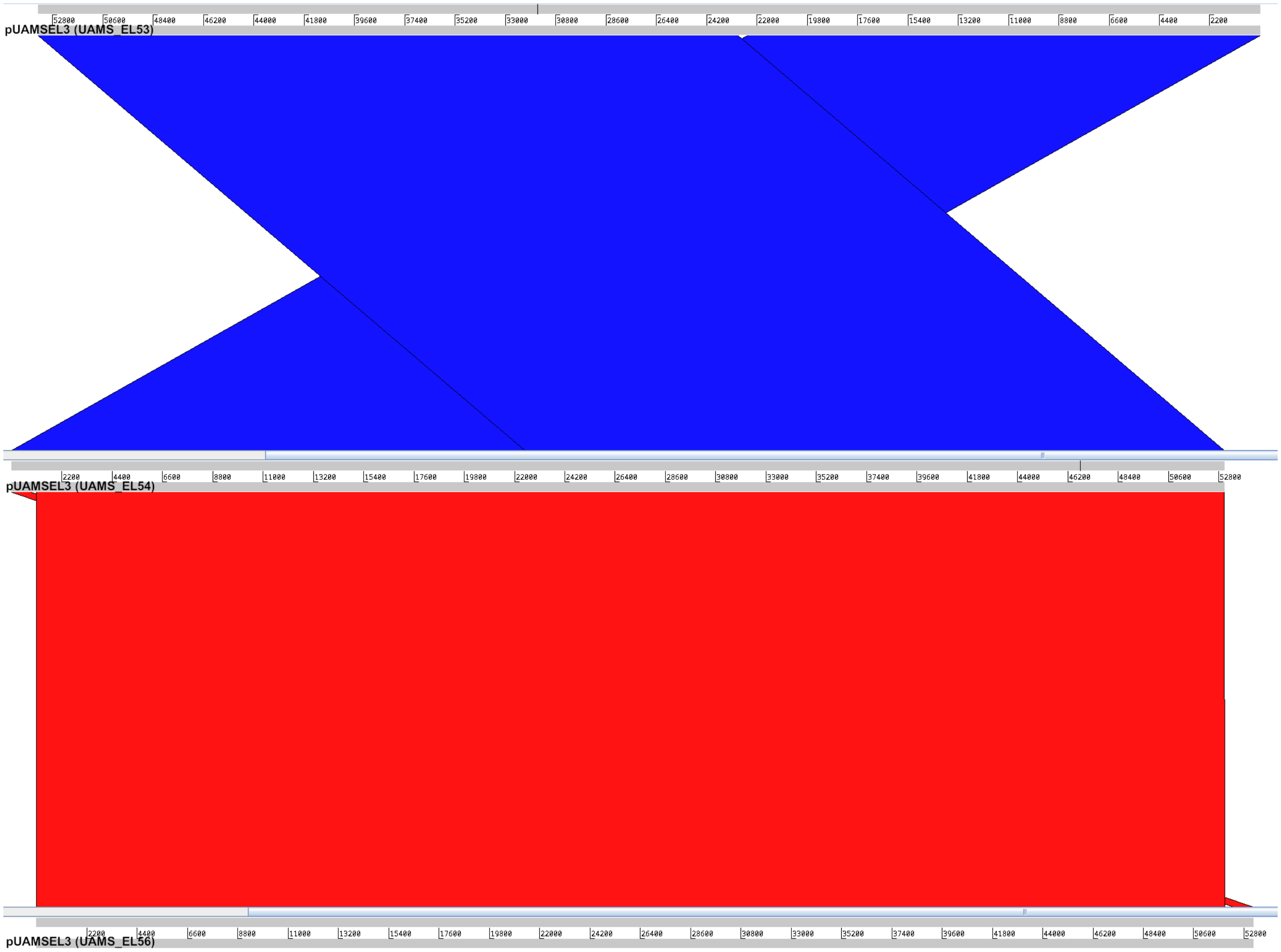

Supplementary Figure 1.F. Representation of the comparison of the plasmid sequence from plasmid pUAMSEL4 of *E. faecalis* isolates UAMS_EL53, UAMS_EL54 and UAMS_EL56 using Artemis Comparison Tool (ACT) release 18.1.0 (1). Comparison files for ACT input were created using blastn pairwise alignments of the three plasmids on the NCBI Blast website. Red and blue bars indicate regions of similarity with red bars indicating corresponding regions that are oriented similarly and blue bars indicating regions oriented in opposite directions.

F.

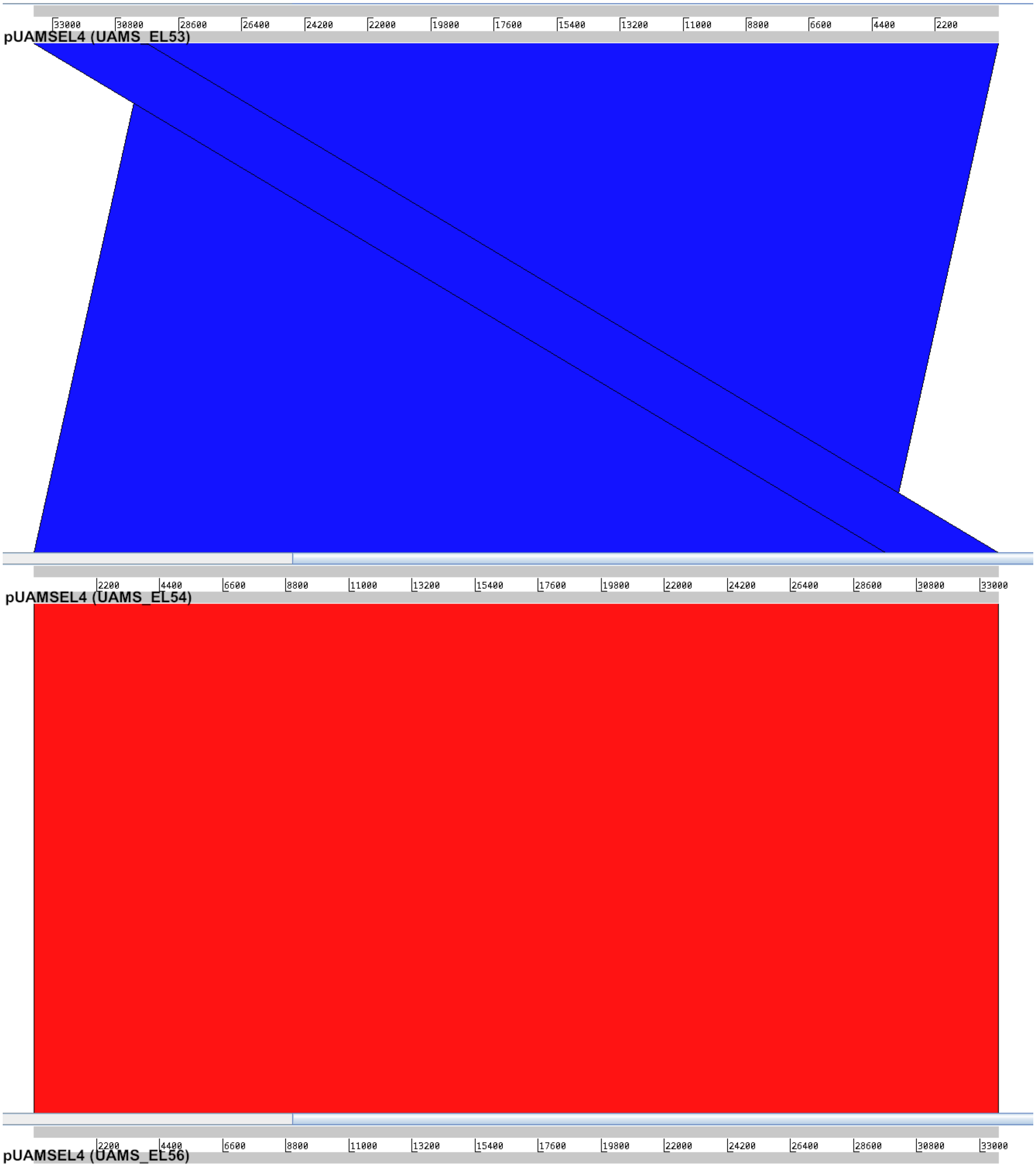

Supplementary Figure 1.G. Representation of the comparison of the plasmid sequence from plasmid pUAMSEF1a and pUAMSEF1b of *E. faecium* isolates UAMS_EL55 and UAMS_EF57 and UAMS_EF58, respectively, using Artemis Comparison Tool (ACT) release 18.1.0 (1). Comparison files for ACT input were created using blastn pairwise alignments of the three plasmids on the NCBI Blast website. Red and blue bars indicate regions of similarity with red bars indicating corresponding regions that are oriented similarly and blue bars indicating regions oriented in opposite directions.

G.

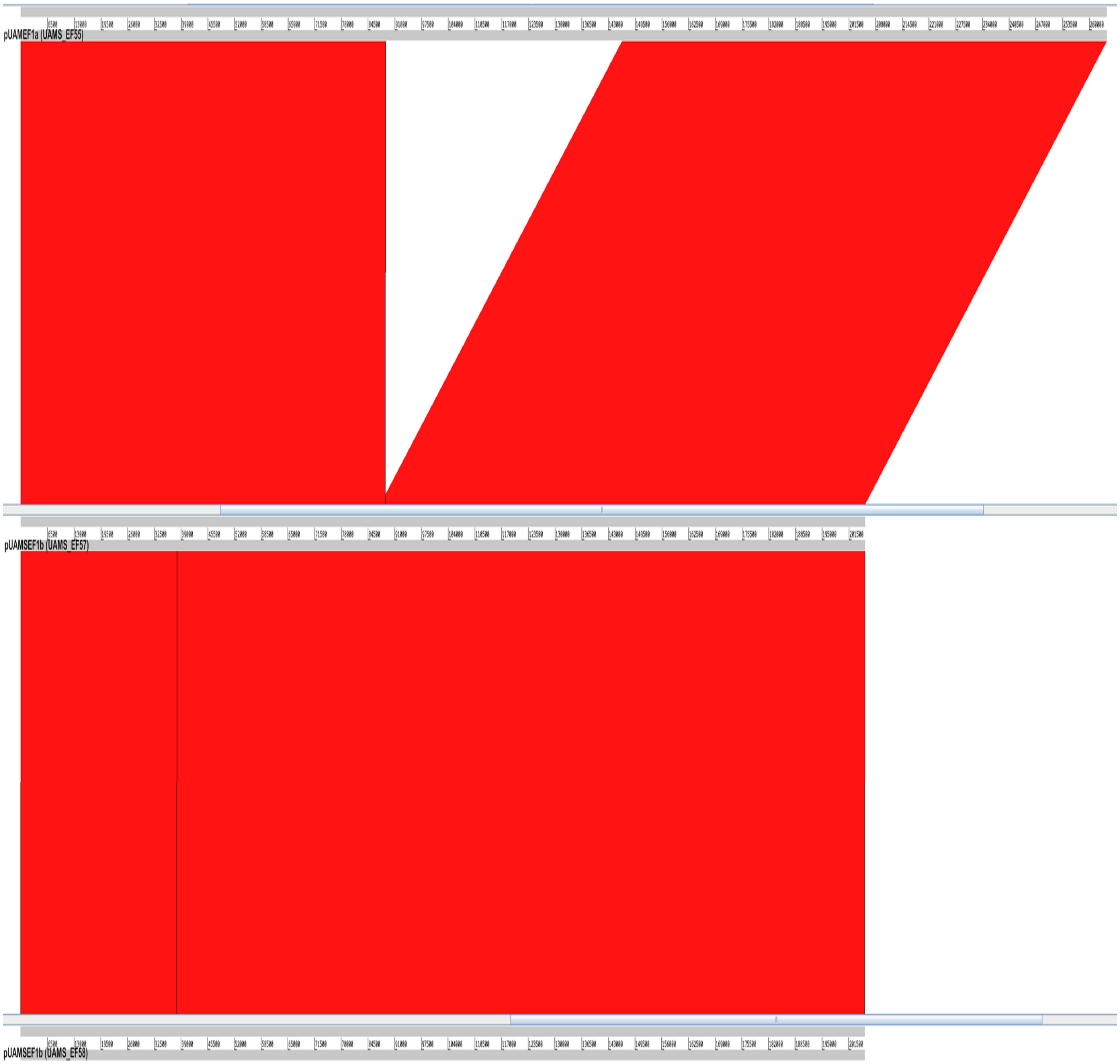

Supplementary Figure 1.H. Representation of the comparison of the plasmid sequence from plasmid pUAMSEF2 of *E. faecium* isolates UAMS_EL55, UAMS_EF57 and UAMS_EF58 using Artemis Comparison Tool (ACT) (1). Comparison files for ACT input were created using blastn pairwise alignments of the three plasmids on the NCBI Blast website. Red and blue bars indicate regions of similarity with red bars indicating corresponding regions that are oriented similarly and blue bars indicating regions oriented in opposite directions.

H.

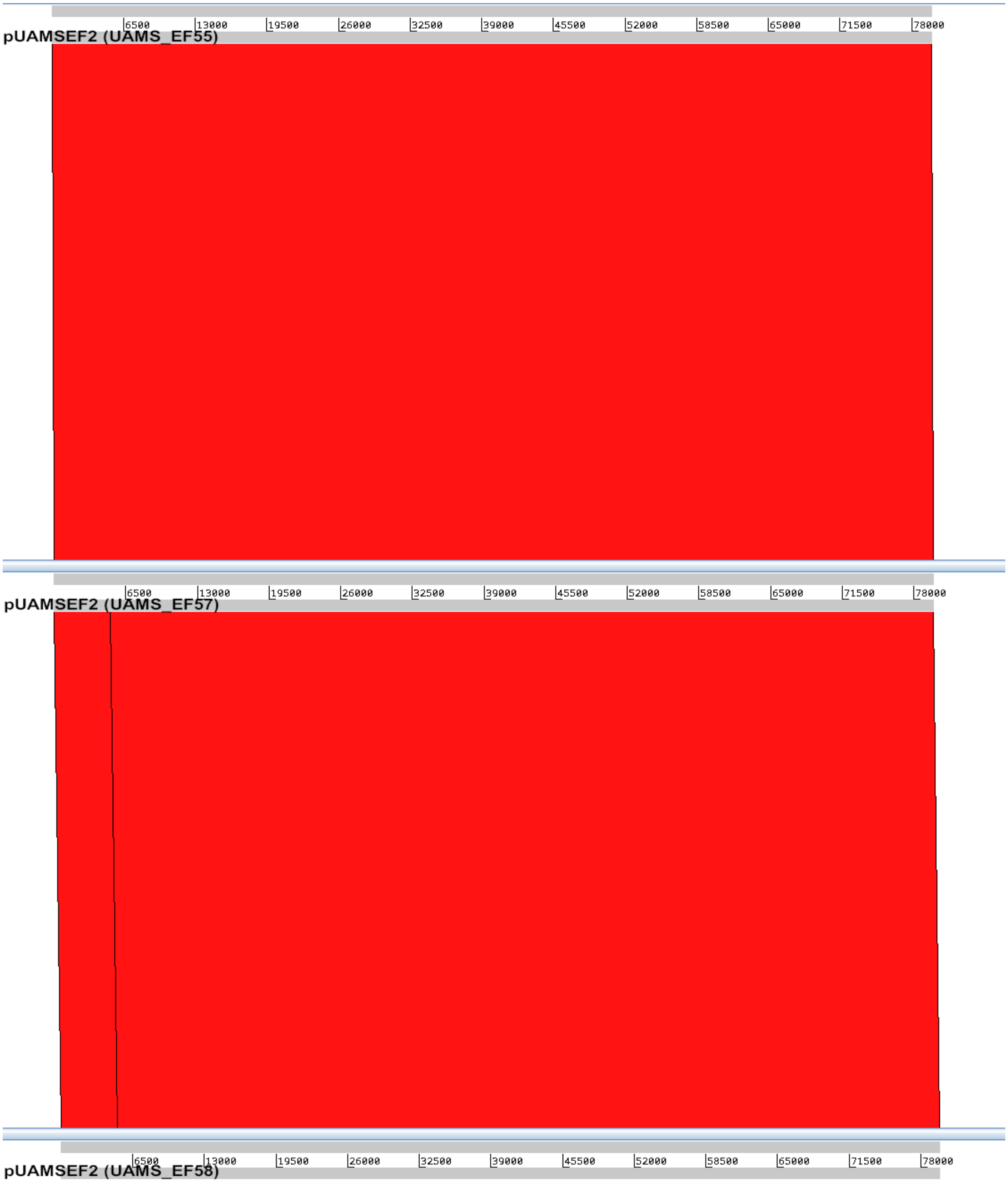

Supplementary Figure 1.I. Representation of the comparison of the plasmid sequence from plasmid pUAMSEF3 of *E. faecium* isolates UAMS_EL55, UAMS_EF57 and UAMS_EF58 using Artemis Comparison Tool (ACT) release 18.1.0 (1). Comparison files for ACT input were created using blastn pairwise alignments of the three plasmids on the NCBI Blast website. Red and blue bars indicate regions of similarity with red bars indicating corresponding regions that are oriented similarly and blue bars indicating regions oriented in opposite directions.

I.

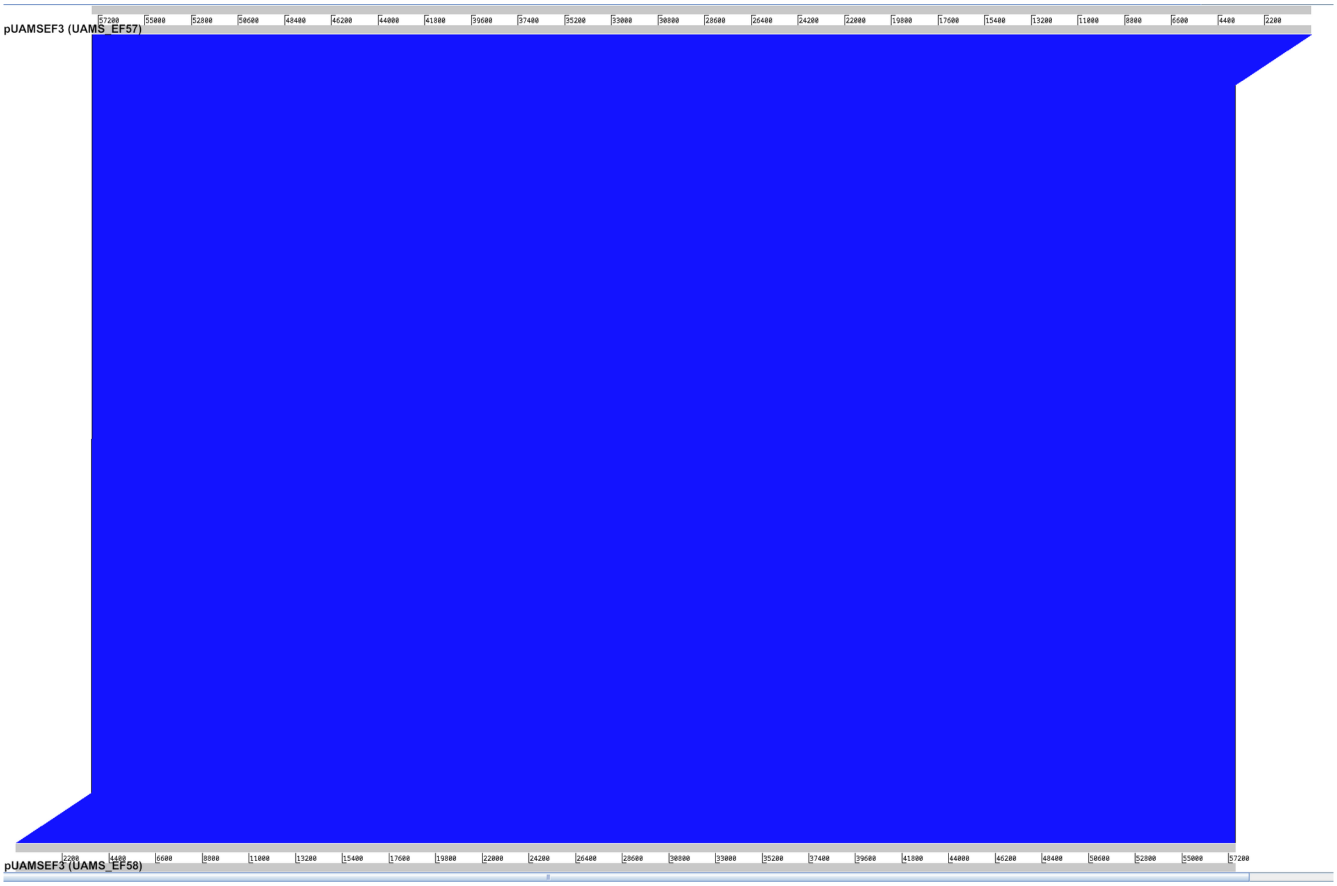

Supplementary Figure 1.J. Representation of the comparison of the plasmid sequence from plasmid pUAMSEF4 of *E. faecium* isolates UAMS_EL55, UAMS_EF57 and UAMS_EF58 using Artemis Comparison Tool (ACT) release 18.1.0 (1). Comparison files for ACT input were created using blastn pairwise alignments of the three plasmids on the NCBI Blast website. Red and blue bars indicate regions of similarity with red bars indicating corresponding regions that are oriented similarly and blue bars indicating regions oriented in opposite directions.

J.

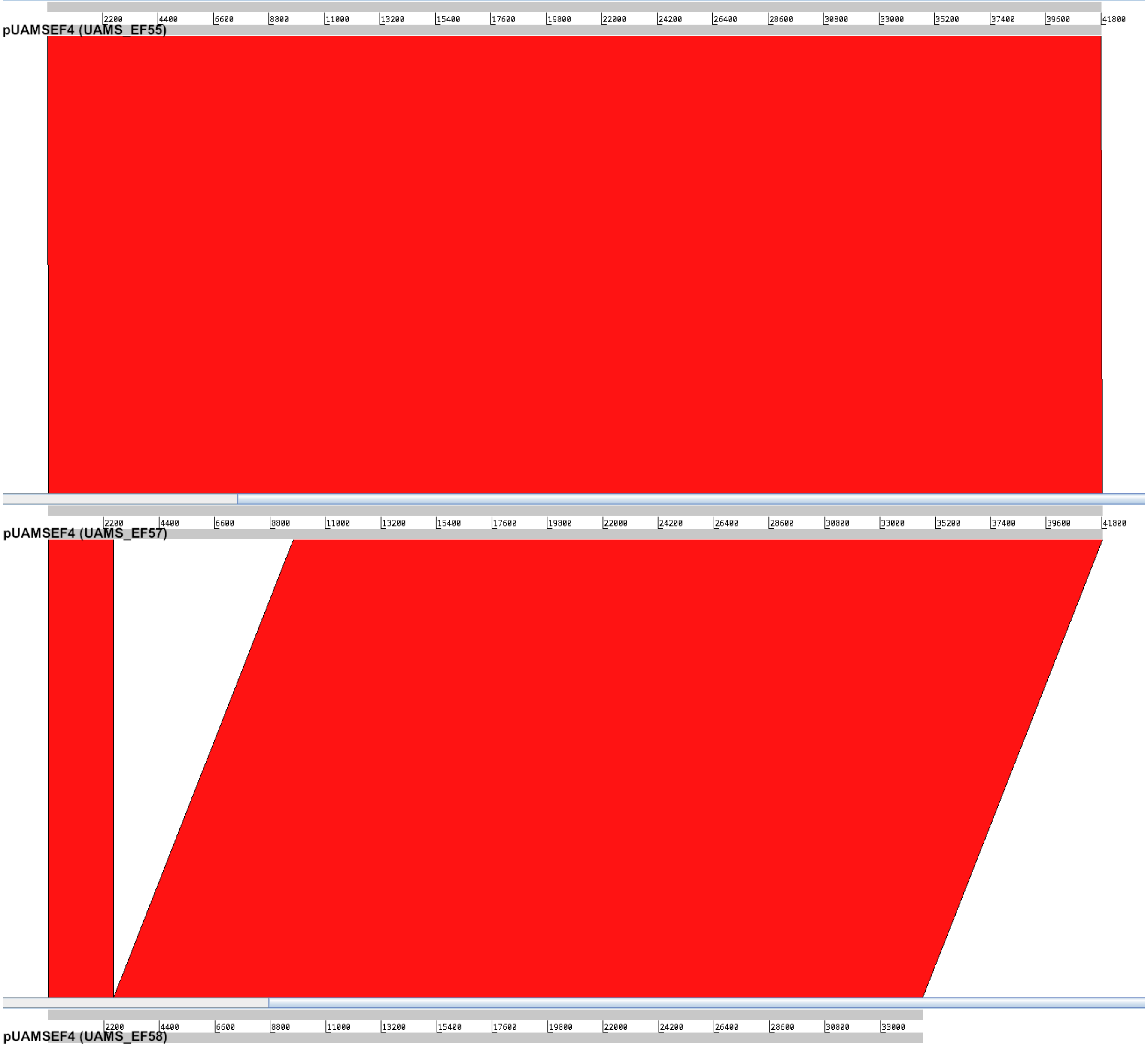

Supplementary Figure 1.K. Representation of the comparison of the plasmid sequence from plasmid pUAMSEF5 of *E. faecium* isolates UAMS_EL55, UAMS_EF57 and UAMS_EF58 using Artemis Comparison Tool (ACT) release 18.1.0 (1). Comparison files for ACT input were created using blastn pairwise alignments of the three plasmids on the NCBI Blast website. Red and blue bars indicate regions of similarity with red bars indicating corresponding regions that are oriented similarly and blue bars indicating regions oriented in opposite directions.

K.

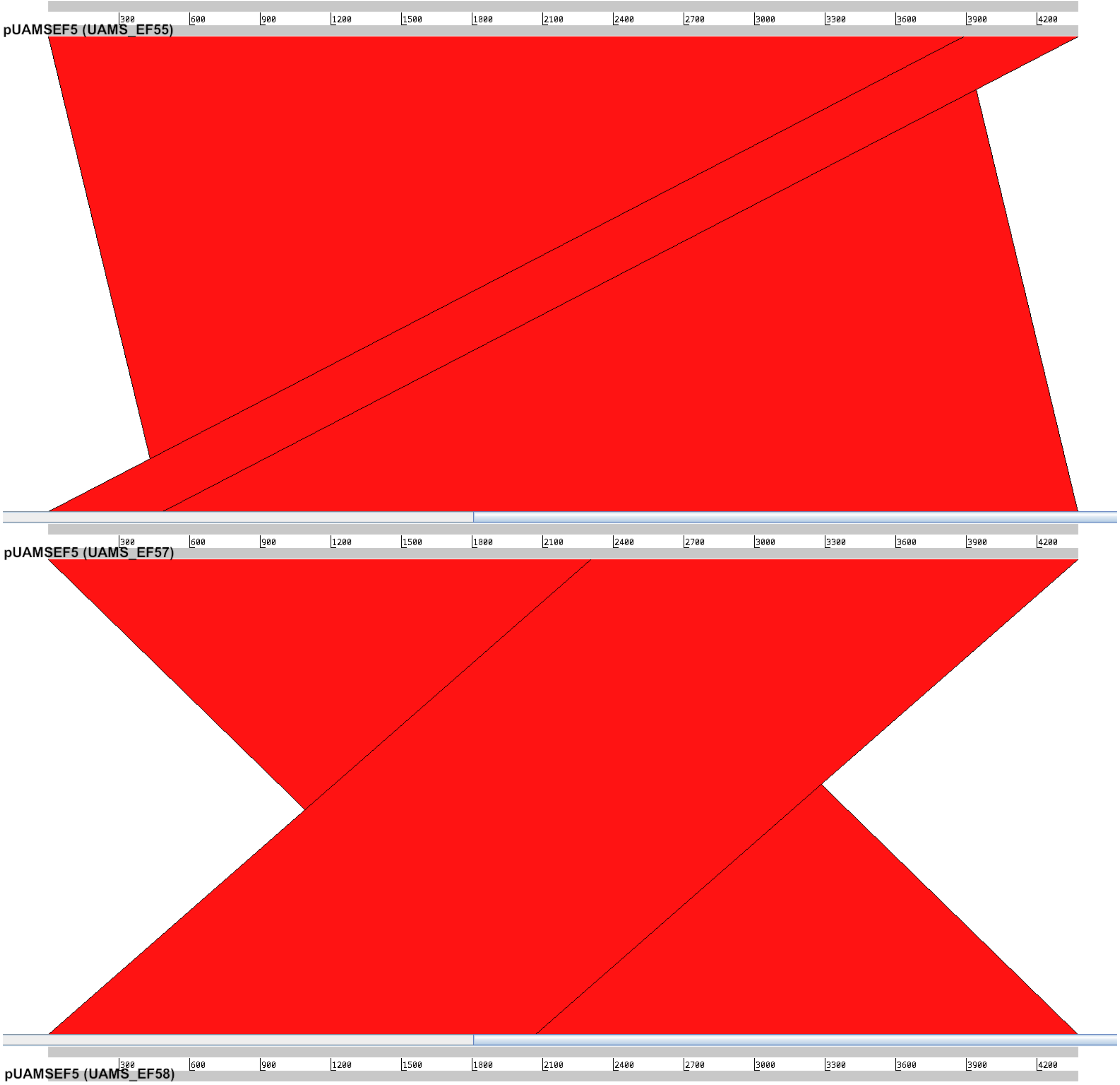

Supplementary Figure 1.L. Dot-plot representation of the comparative analysis of the genome sequences from isolates UAMS_EL53 and UAMS_EL54 using minimap2.2 (2). Dot-plots were created using dotPlotly library using R (https://github.com/tpoorten/dotPlotly). Dots were colored according to query sequence similarity.

L.

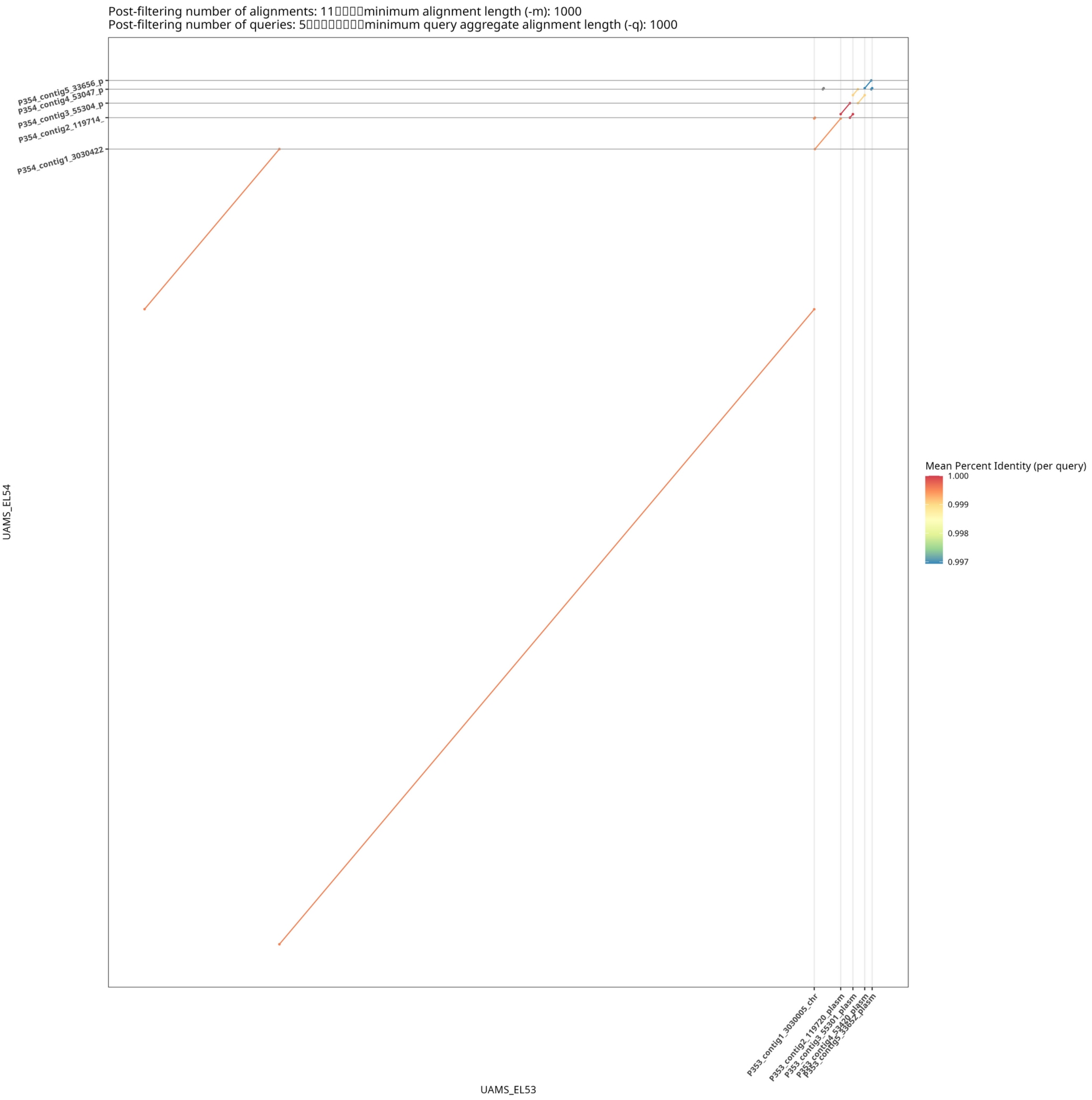

Supplementary Figure 1.M. Dot-plot representation of the comparative analysis of the genome sequences from isolates UAMS_EL53 and UAMS_EF55 using minimap2.2 (2). Dot-plots were created using dotPlotly library using R (https://github.com/tpoorten/dotPlotly). Dots were colored according to query sequence similarity.

M.

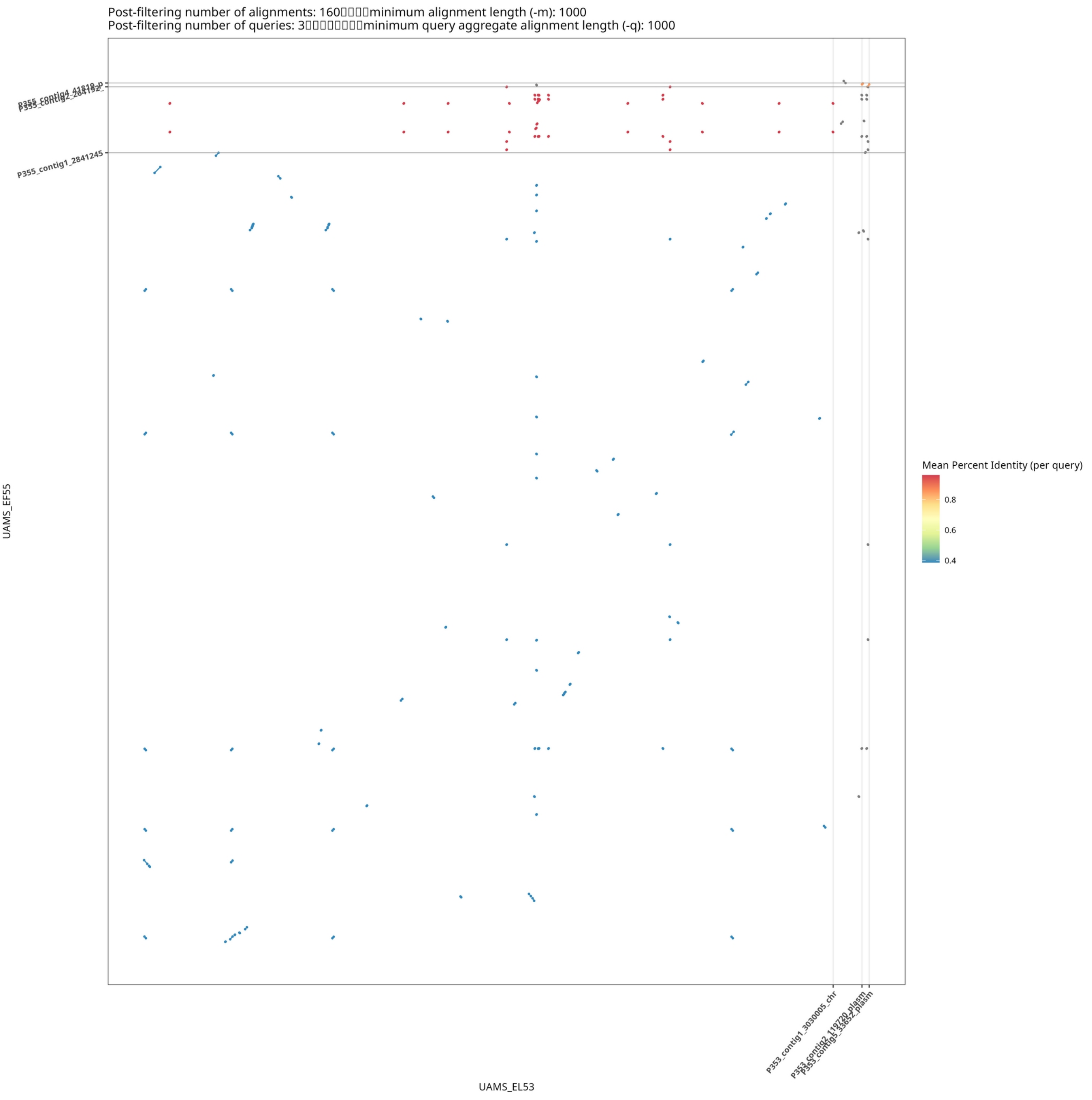

Supplementary Figure 1.N. Dot-plot representation of the comparative analysis of the genome sequences from isolates UAMS_EL53 and UAMS_EL56 using minimap2.2 (2). Dot-plots were created using dotPlotly library using R (https://github.com/tpoorten/dotPlotly). Dots were colored according to query sequence similarity.

N.

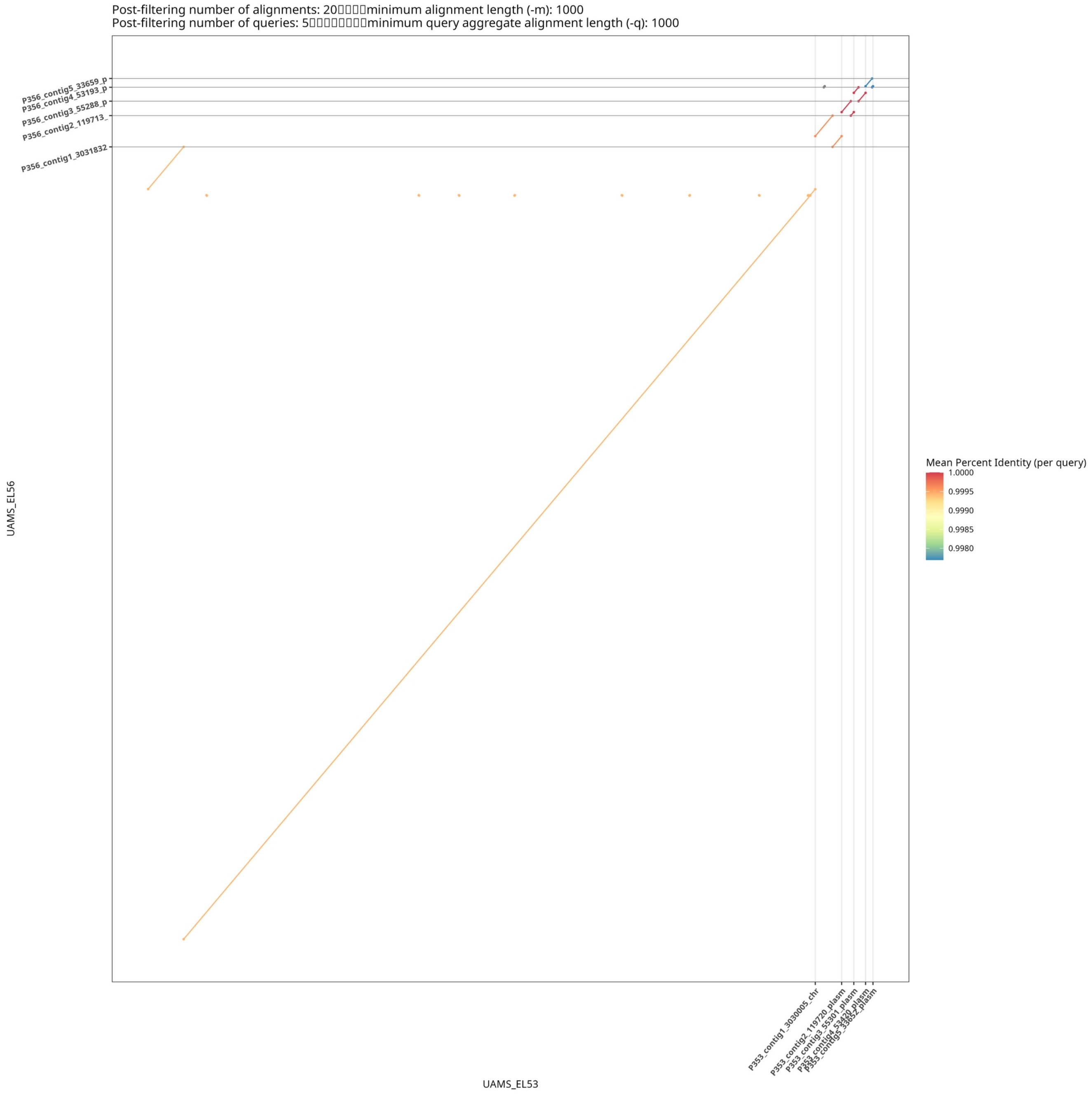

Supplementary Figure 1.O. Dot-plot representation of the comparative analysis of the genome sequences from isolates UAMS_EL53 and UAMS_EF57 using minimap2.2 (2). Dot-plots were created using dotPlotly library using R (https://github.com/tpoorten/dotPlotly). Dots were colored according to query sequence similarity.

O.

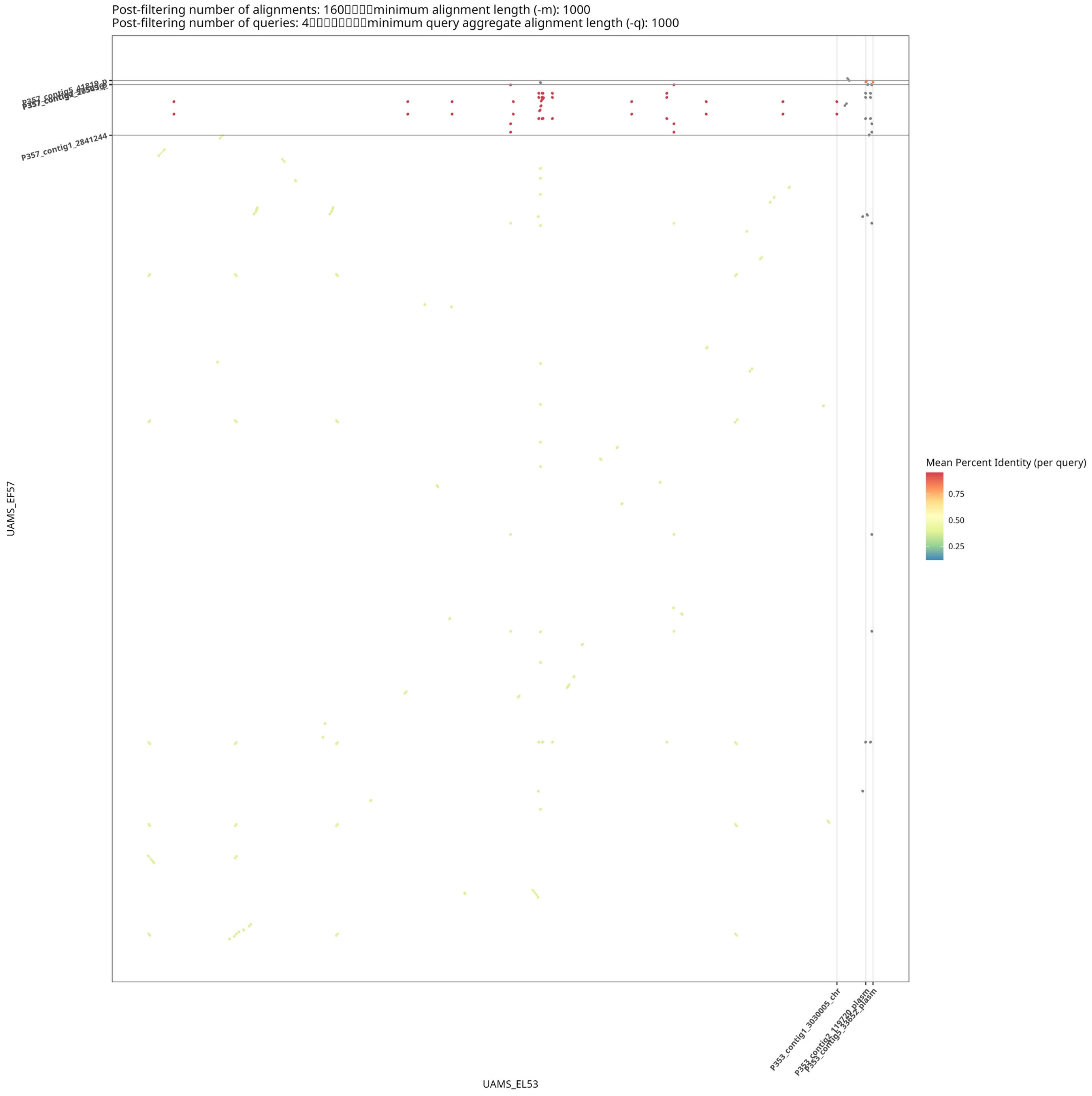

Supplementary Figure 1.P. Dot-plot representation of the comparative analysis of the genome sequences from isolates UAMS_EL53 and UAMS_EF58 using minimap2.2 (2). Dot-plots were created using dotPlotly library using R (https://github.com/tpoorten/dotPlotly). Dots were colored according to query sequence similarity.

P.

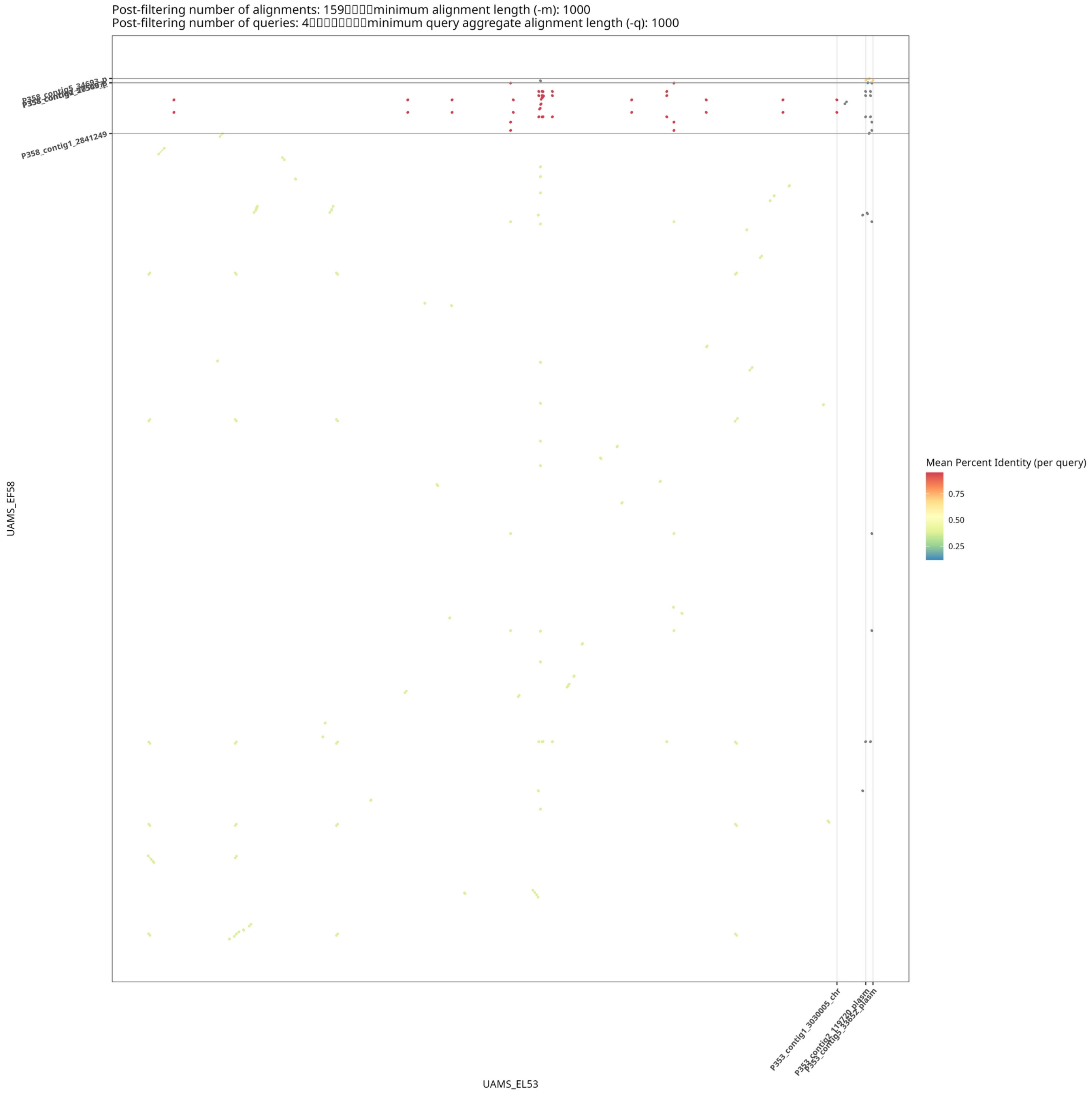

Supplementary Figure 1.Q. Dot-plot representation of the comparative analysis of the genome sequences from isolates UAMS_EL54 and UAMS_EF55 using minimap2.2 (2). Dot-plots were created using dotPlotly library using R (https://github.com/tpoorten/dotPlotly). Dots were colored according to query sequence similarity.

Q.

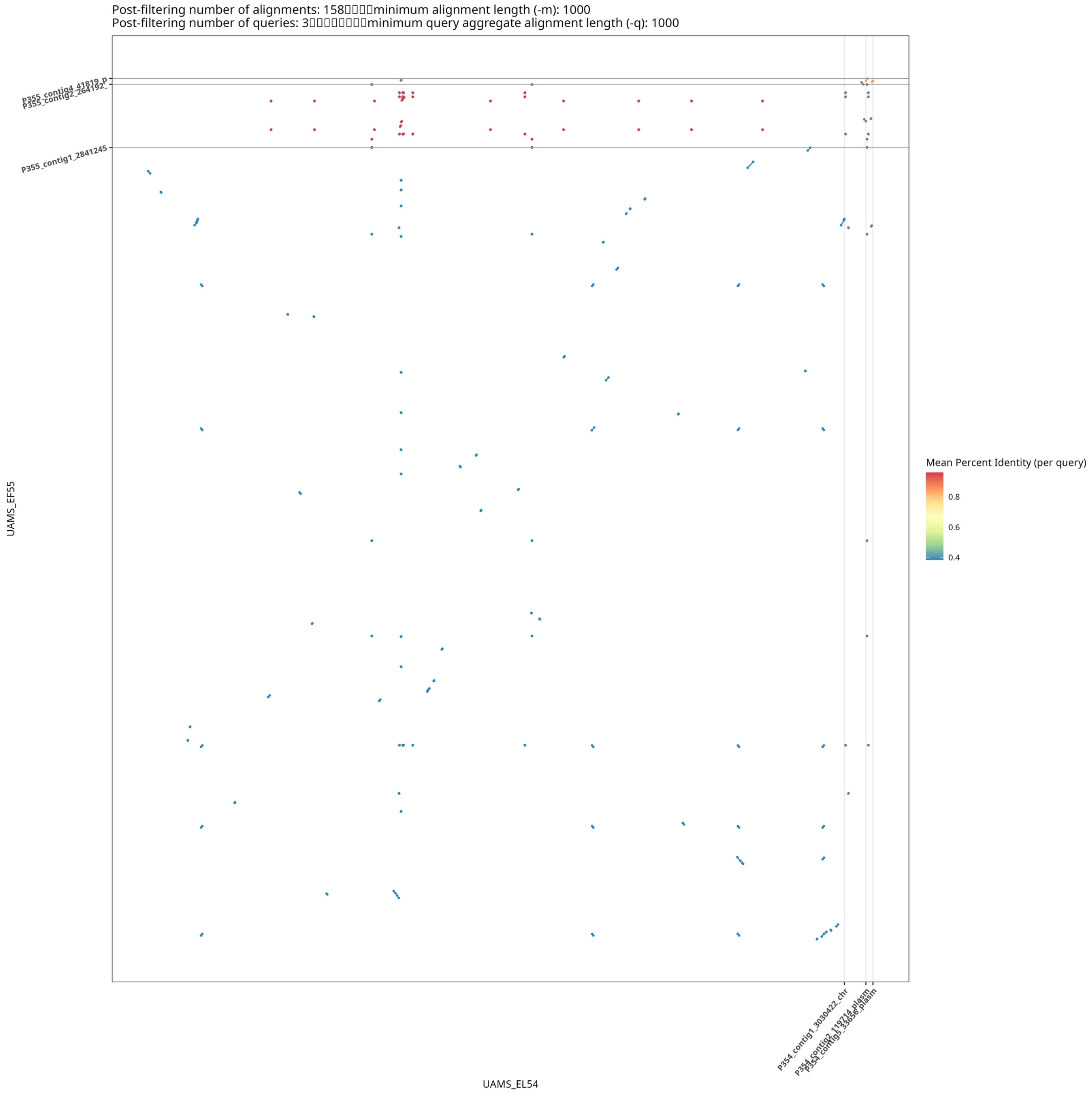

Supplementary Figure 1.R. Dot-plot representation of the comparative analysis of the genome sequences from isolates UAMS_EL54 and UAMS_EL56 using minimap2.2 (2). Dot-plots were created using dotPlotly library using R (https://github.com/tpoorten/dotPlotly). Dots were colored according to query sequence similarity.

R.

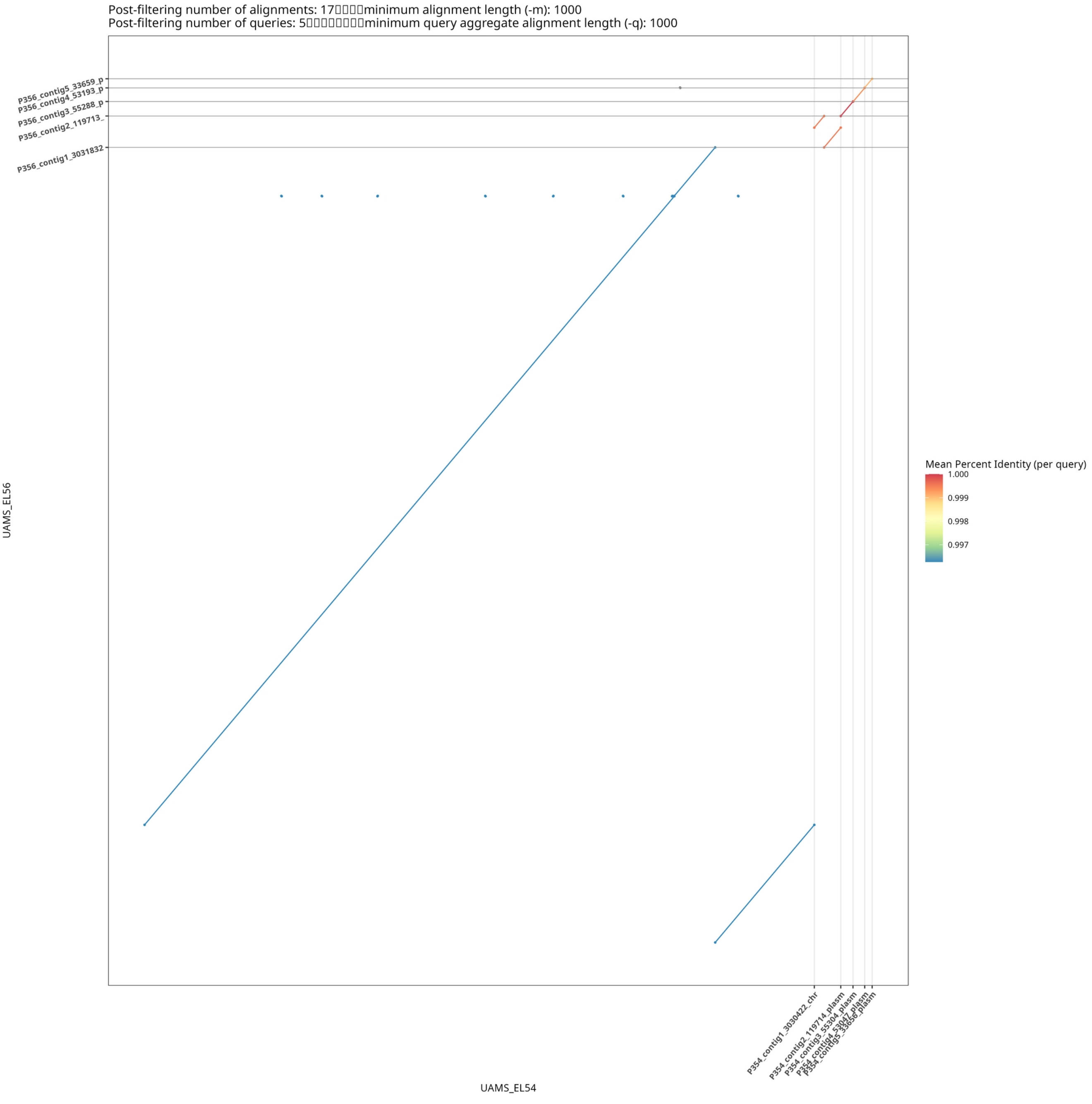

Supplementary Figure 1.S. Dot-plot representation of the comparative analysis of the genome sequences from isolates UAMS_EL54 and UAMS_EF57 using minimap2.2 (2). Dot-plots were created using dotPlotly library using R (https://github.com/tpoorten/dotPlotly). Dots were colored according to query sequence similarity.

S.

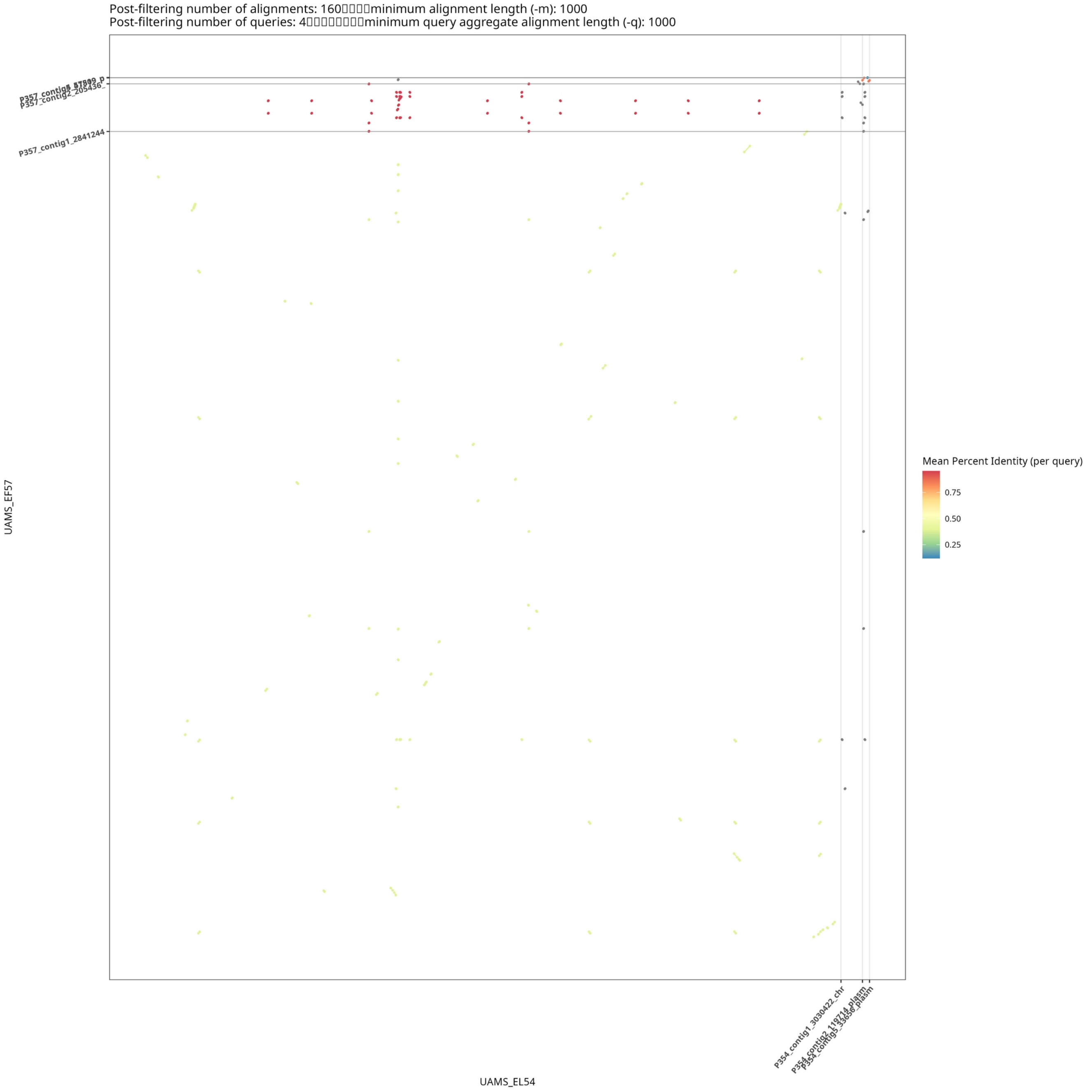

Supplementary Figure 1.T. Supplementary Figure 1.S. Dot-plot representation of the comparative analysis of the genome sequences from isolates UAMS_EL54 and UAMS_EF58 using minimap2.2 (2). Dot-plots were created using dotPlotly library using R (https://github.com/tpoorten/dotPlotly). Dots were colored according to query sequence similarity.

T.

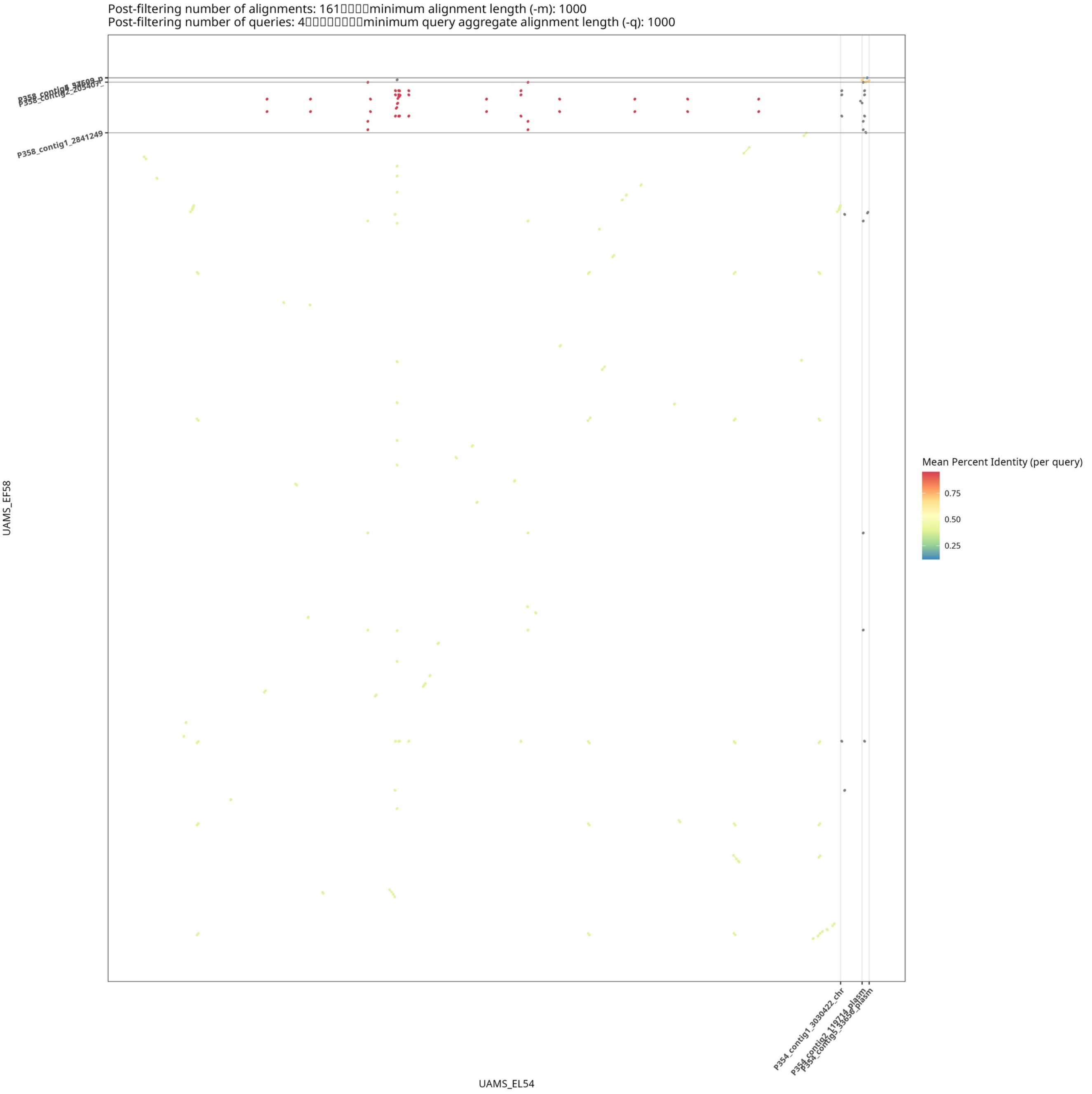

Supplementary Figure 1.U. Supplementary Figure 1.S. Dot-plot representation of the comparative analysis of the genome sequences from isolates UAMS_EF55 and UAMS_EL56 using minimap2.2 (2). Dot-plots were created using dotPlotly library using R (https://github.com/tpoorten/dotPlotly). Dots were colored according to query sequence similarity.

U.

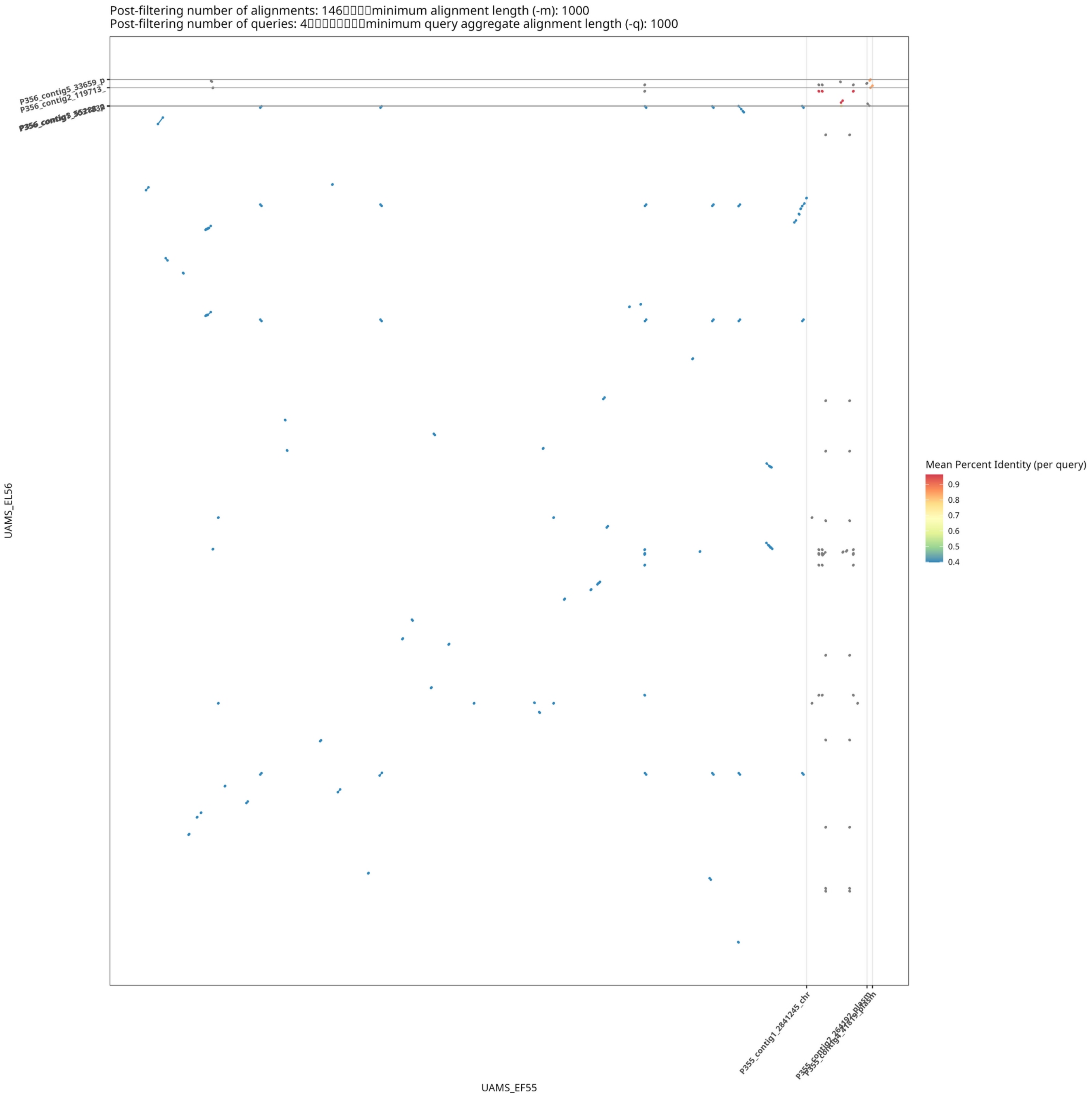

Supplementary Figure 1.V. Supplementary Figure 1.S. Dot-plot representation of the comparative analysis of the genome sequences from isolates UAMS_EF55 and UAMS_EF57 using minimap2.2 (2). Dot-plots were created using dotPlotly library using R (https://github.com/tpoorten/dotPlotly). Dots were colored according to query sequence similarity.

V.

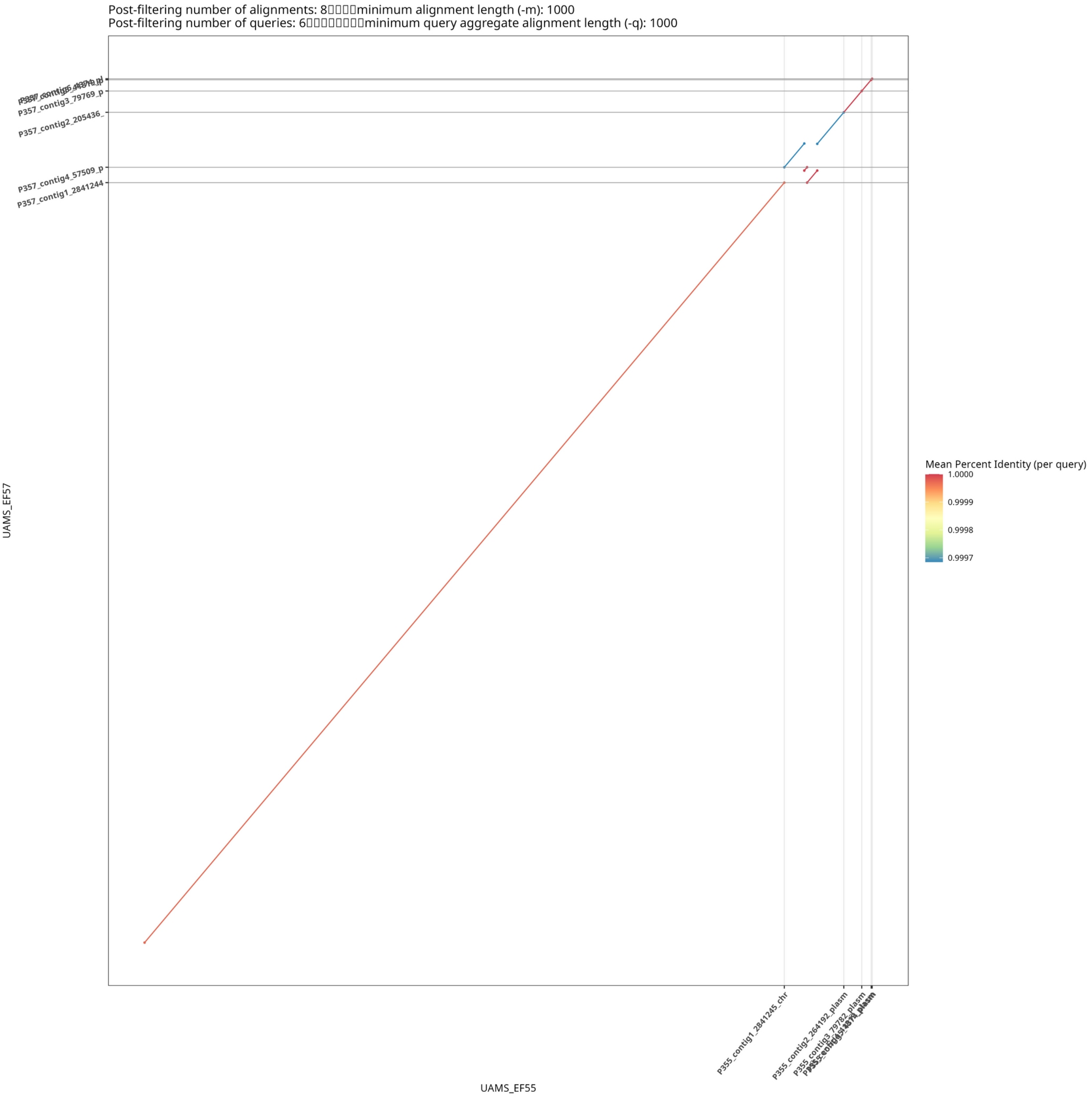

Supplementary Figure 1.W. Supplementary Figure 1.V. Supplementary Figure 1.S. Dot-plot representation of the comparative analysis of the genome sequences from isolates UAMS_EF55 and UAMS_EF58 using minimap2.2 (2). Dot-plots were created using dotPlotly library using R (https://github.com/tpoorten/dotPlotly). Dots were colored according to query sequence similarity.

W.

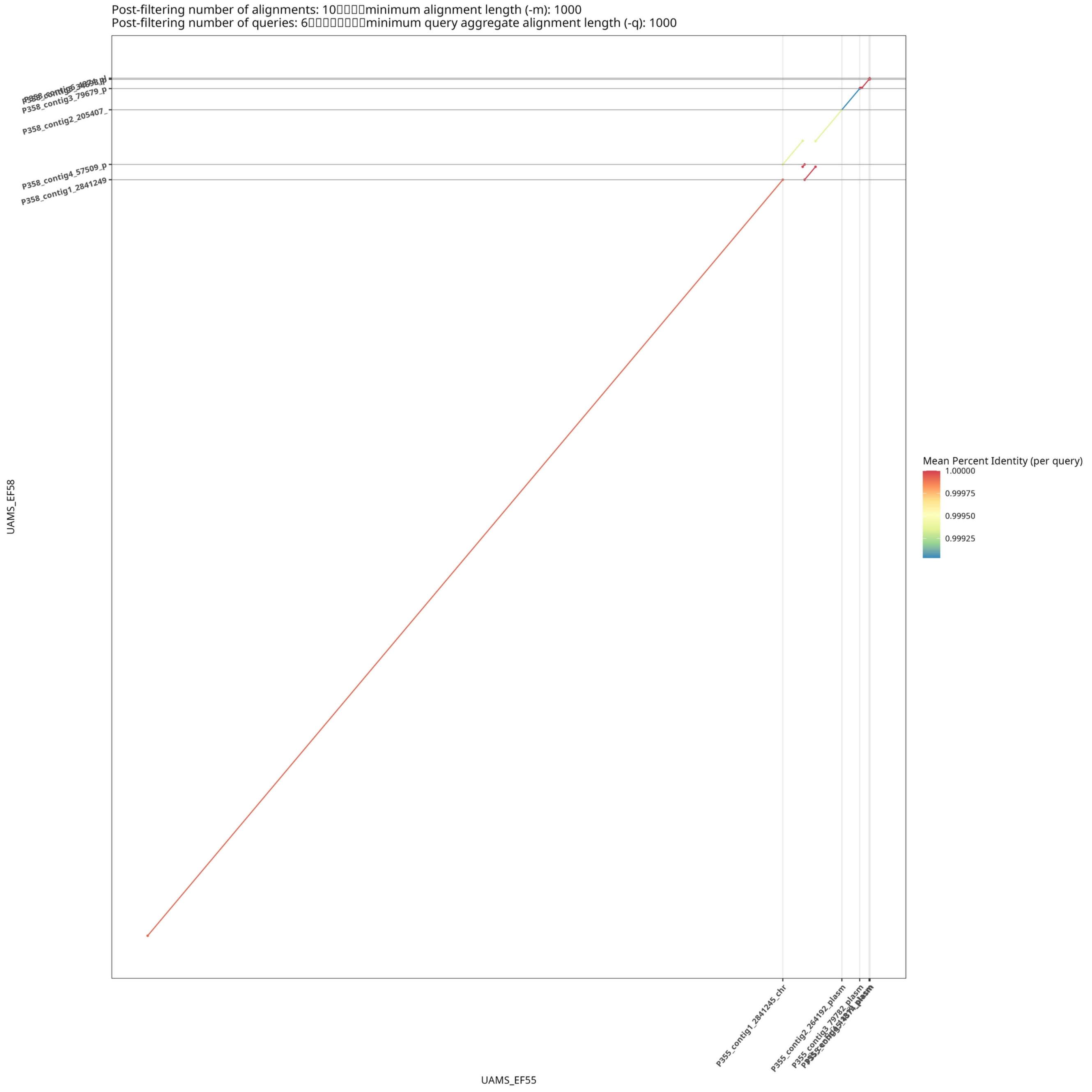

Supplementary Figure 1.X. Supplementary Figure 1.V. Supplementary Figure 1.S. Dot-plot representation of the comparative analysis of the genome sequences from isolates UAMS_EL56 and UAMS_EF57 using minimap2.2 (2). Dot-plots were created using dotPlotly library using R (https://github.com/tpoorten/dotPlotly). Dots were colored according to query sequence similarity.

X.

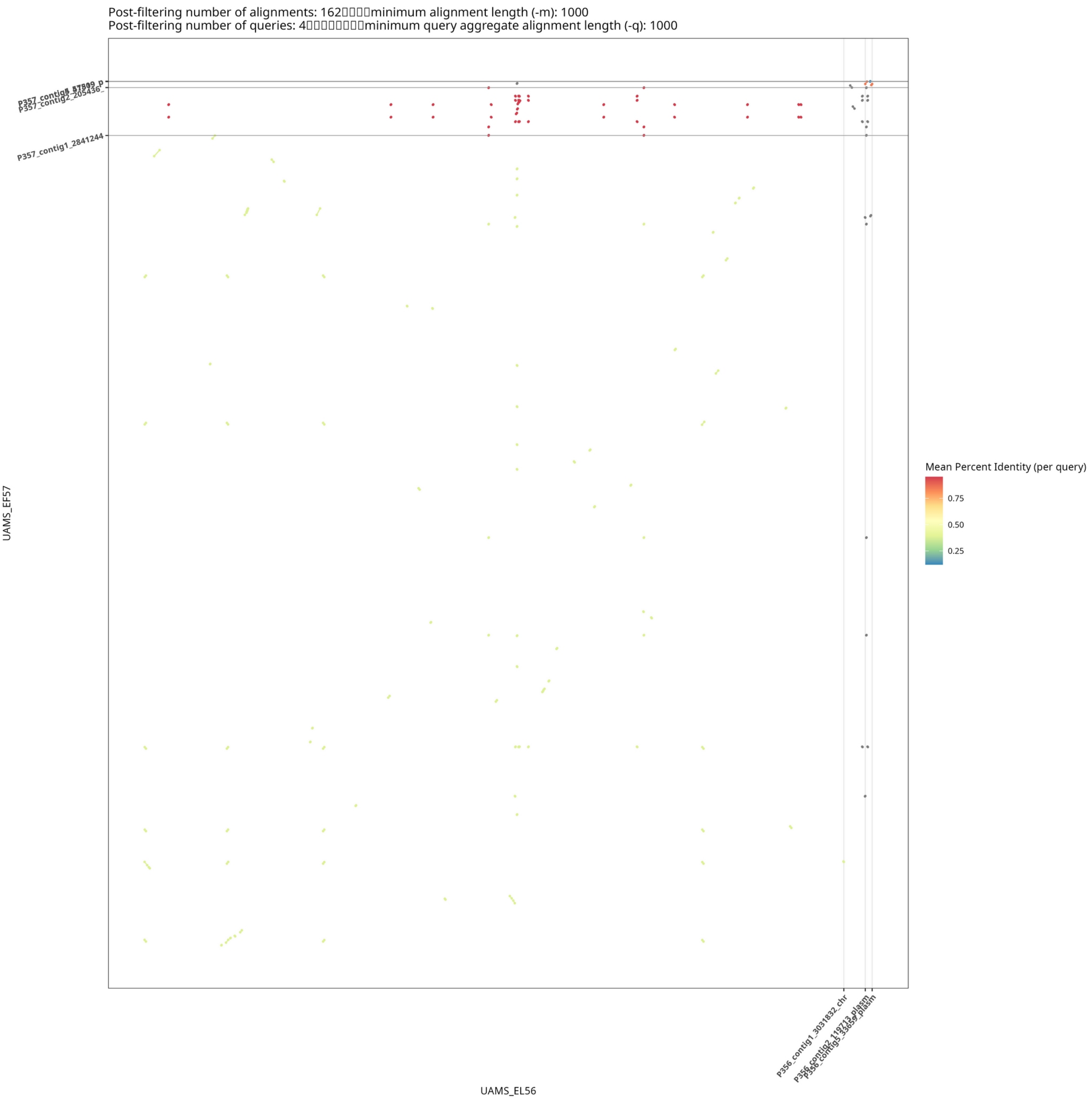

Supplementary Figure 1.Y. Supplementary Figure 1.V. Supplementary Figure 1.S. Dot-plot representation of the comparative analysis of the genome sequences from isolates UAMS_EL56 and UAMS_EF58 using minimap2.2 (2). Dot-plots were created using dotPlotly library using R (https://github.com/tpoorten/dotPlotly). Dots were colored according to query sequence similarity.

Y.

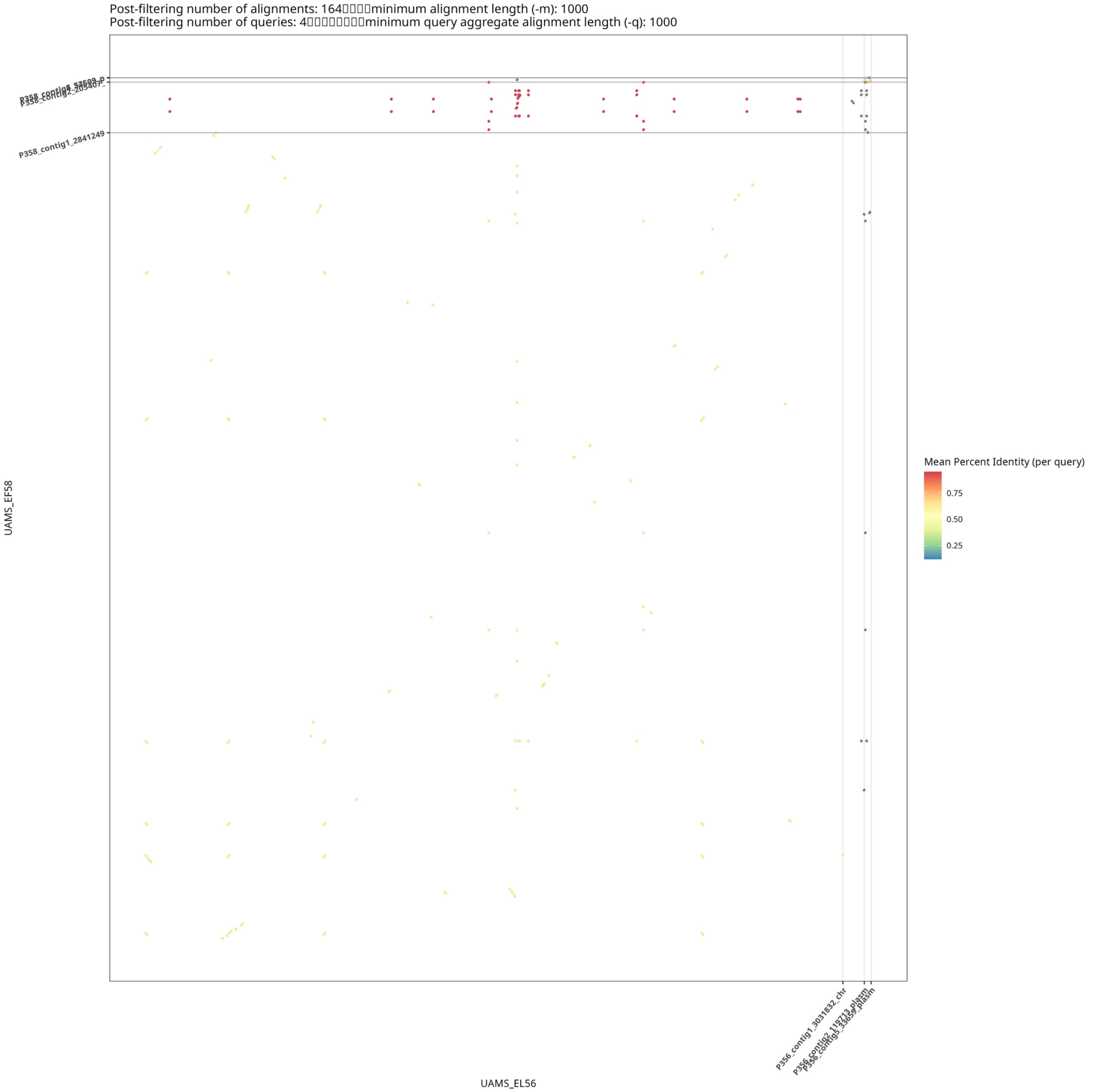

Supplementary Figure 1.Z. Supplementary Figure 1.V. Supplementary Figure 1.S. Dot-plot representation of the comparative analysis of the genome sequences from isolates UAMS_EF57 and UAMS_EF58 using minimap2.2 (2). Dot-plots were created using dotPlotly library using R (https://github.com/tpoorten/dotPlotly). Dots were colored according to query sequence similarity.

Z.

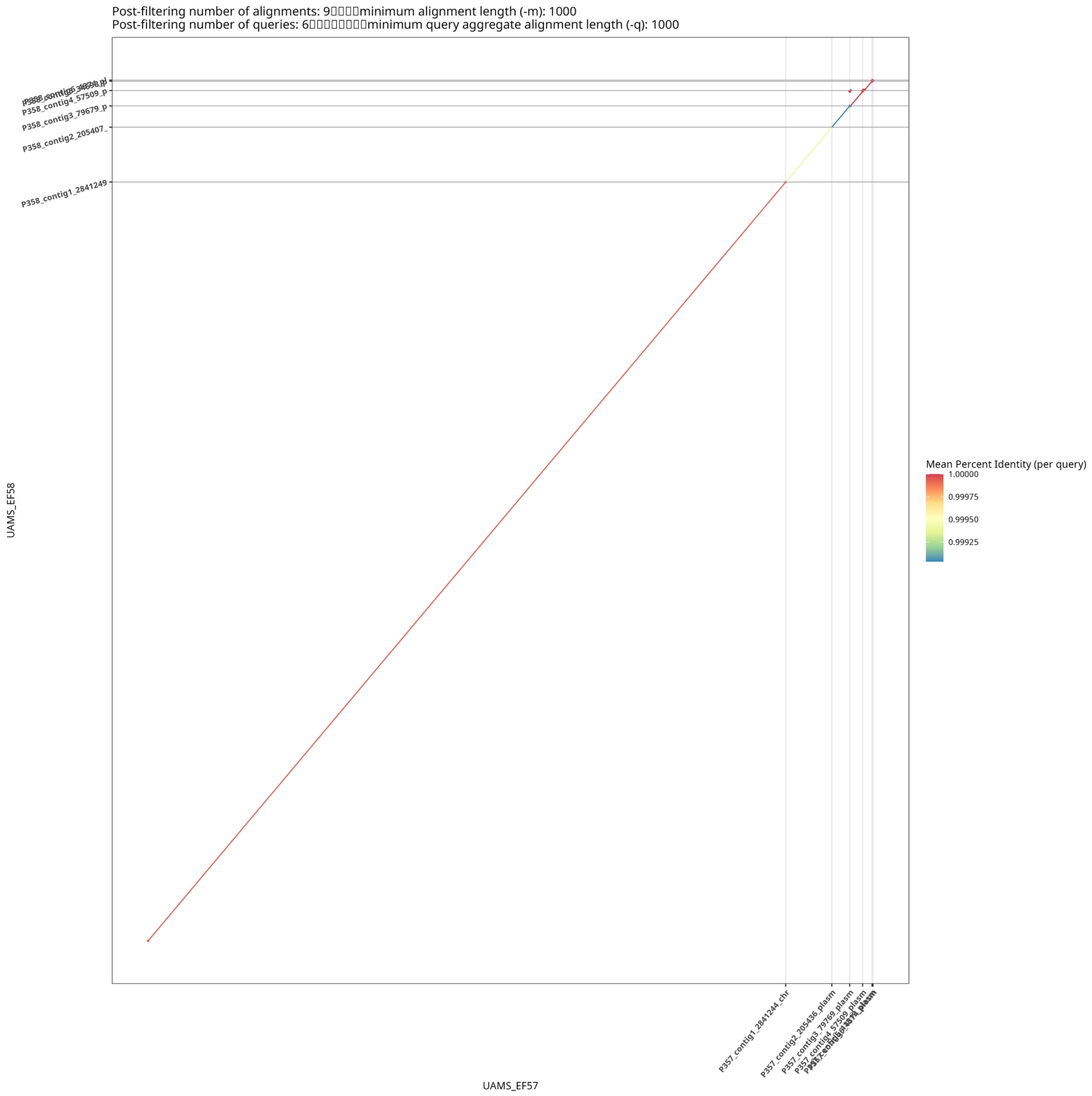

Supplementary Figure 2. (A) A tree of Mash distances based on the plasmid sequences. (B) A table which shows mash distances between the complete genomes utilized in this study.

A

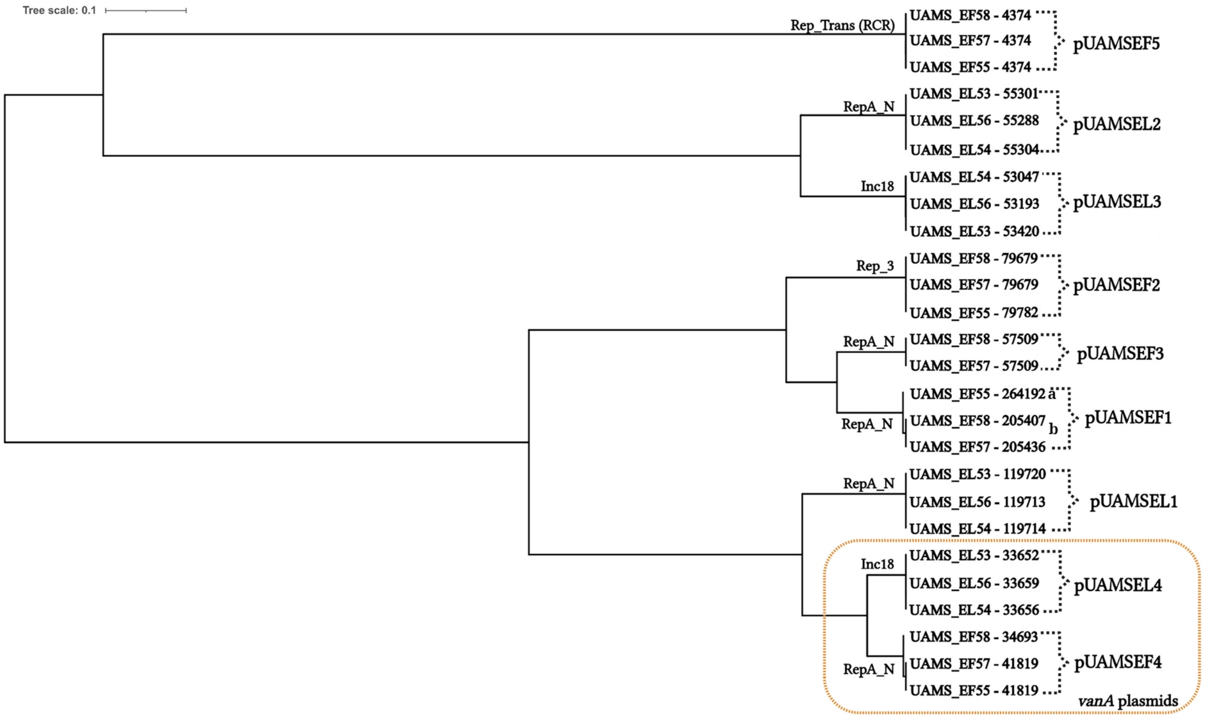

B

Query/Ref	UAMS_EL53	UAMS_EL54	UAMS_EL56	UAMS_EF55	UAMS_EF57	UAMS_EL58
UAMS_EL53	0	7.14446E-06	7.14446E-06	0.178173	0.178173	0.181843
UAMS_EL54	7.14446E-06	0	9.52667E-06	0.178173	0.178173	0.181843
UAMS_EL56	7.14446E-06	9.52667E-06	0	0.178173	0.178173	0.181843
UAMS_EF55	0.178173	0.178173	0.178173	0	2.38113E-06	6.44162E-05
UAMS_EF57	0.178173	0.178173	0.178173	2.38113E-06	0	6.6807E-05
UAMS_EL58	0.181843	0.181843	0.181843	6.44162E-05	6.6807E-05	0

Supplementary Figure 3. Overview of the alignment of the Oxford Nanopore reads from UAMS_EF58 against pUAMSEF1a plasmid from UAMS_EF55. The area of the IS-mediated excision events that generated pUAMSEF3 in UAMSEF_57 and UAMS_EF58 is pointed out. In the central region of the figure, the discontinuity produced by the reads from pUAMSEF3 inserted in pUAMSEF1a can be observed (black arrows). Nanopore read depth in this area was >400x.

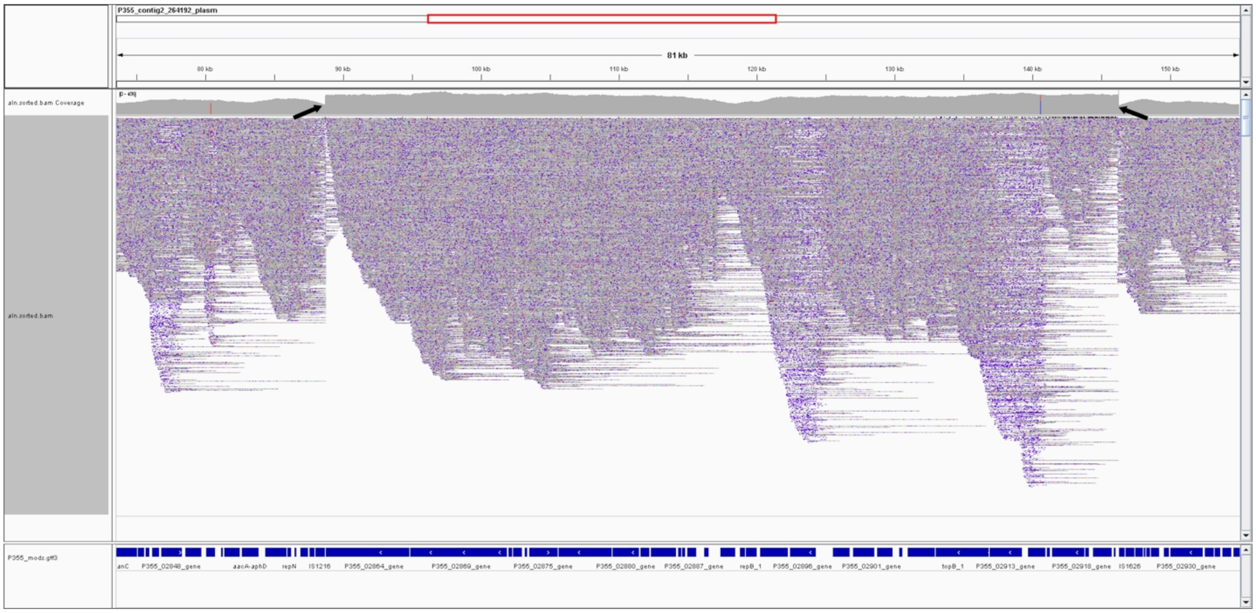

Supplementary Figure 4. Circular representation of pUAMSEF1b plasmid from UAMSEF_57 and UAMSEF_58 isolates.

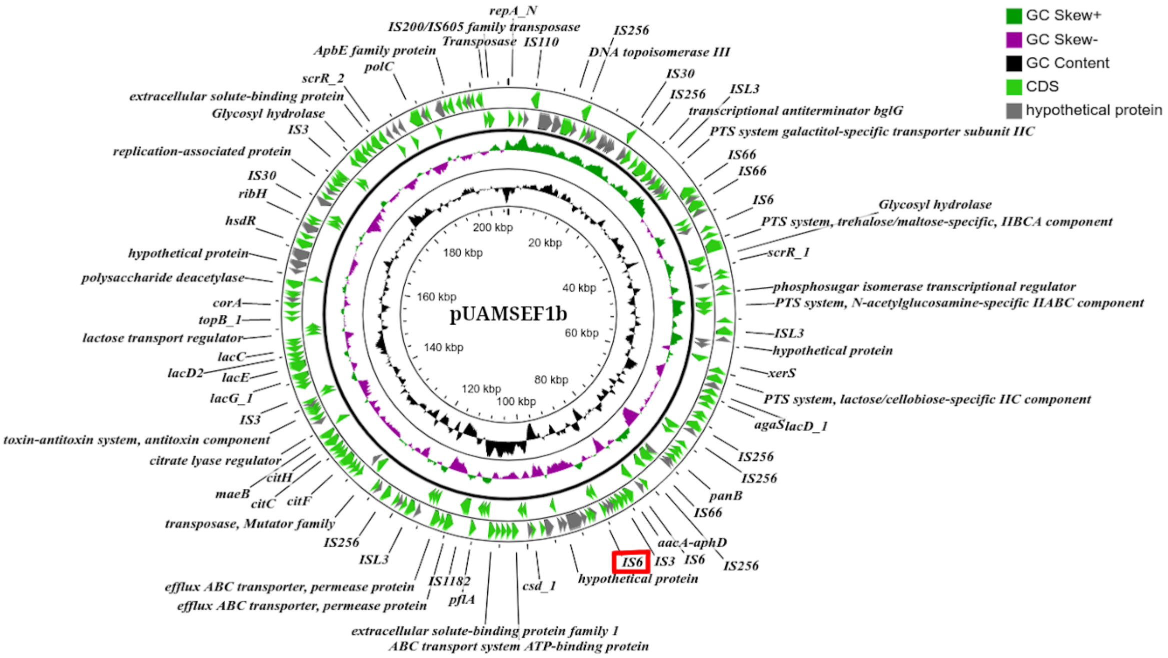

Supplementary Figure 5. Representation of clusters of genes from 33 complete *E. faecium* genomes that harbor mutations in *liaSR* genes (LiaS^{T120A}, LiaR^{W73C}) that are surrounded by IS*L3* elements. Numbers below coding genes represent sequence similarity in percentage between the orthologs in the cluster. The similarity is not shown if two orthologs have sequence similarity < 0.5 (50%). Genes sharing the same color have the same functional annotation and have a high level of sequence similarity. Genes without orthologs in this figure, were represented in grey color.

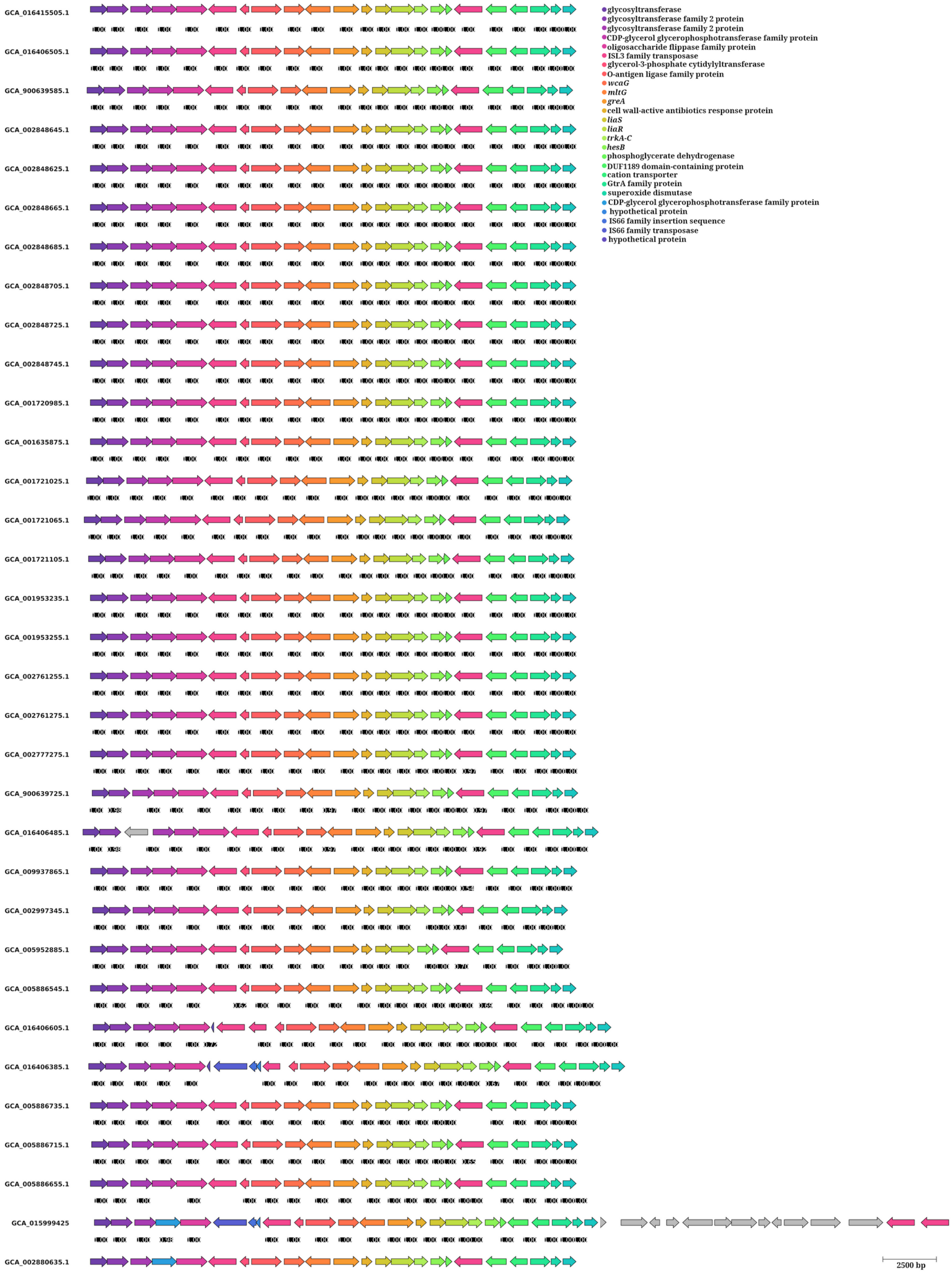

Supplementary Figure 6. Multiple genome alignment of *E. feacium* and *E. faecalis* replicons in which region represented in Figure 3B was preserved. Alignments were visualized using Gmaj software (https://globin.bx.psu.edu/dist/gmaj/). Plasmid pUAMSEF4 from UAMS_EF55 was used as reference for Gmaj software. Regions that align with coordinates 2412..9713 from pUAMSEF4 are market in red color. A zoomable version of this figure is also available at the following DOI on Zenodo: https://doi.org/10.5281/zenodo.6513079

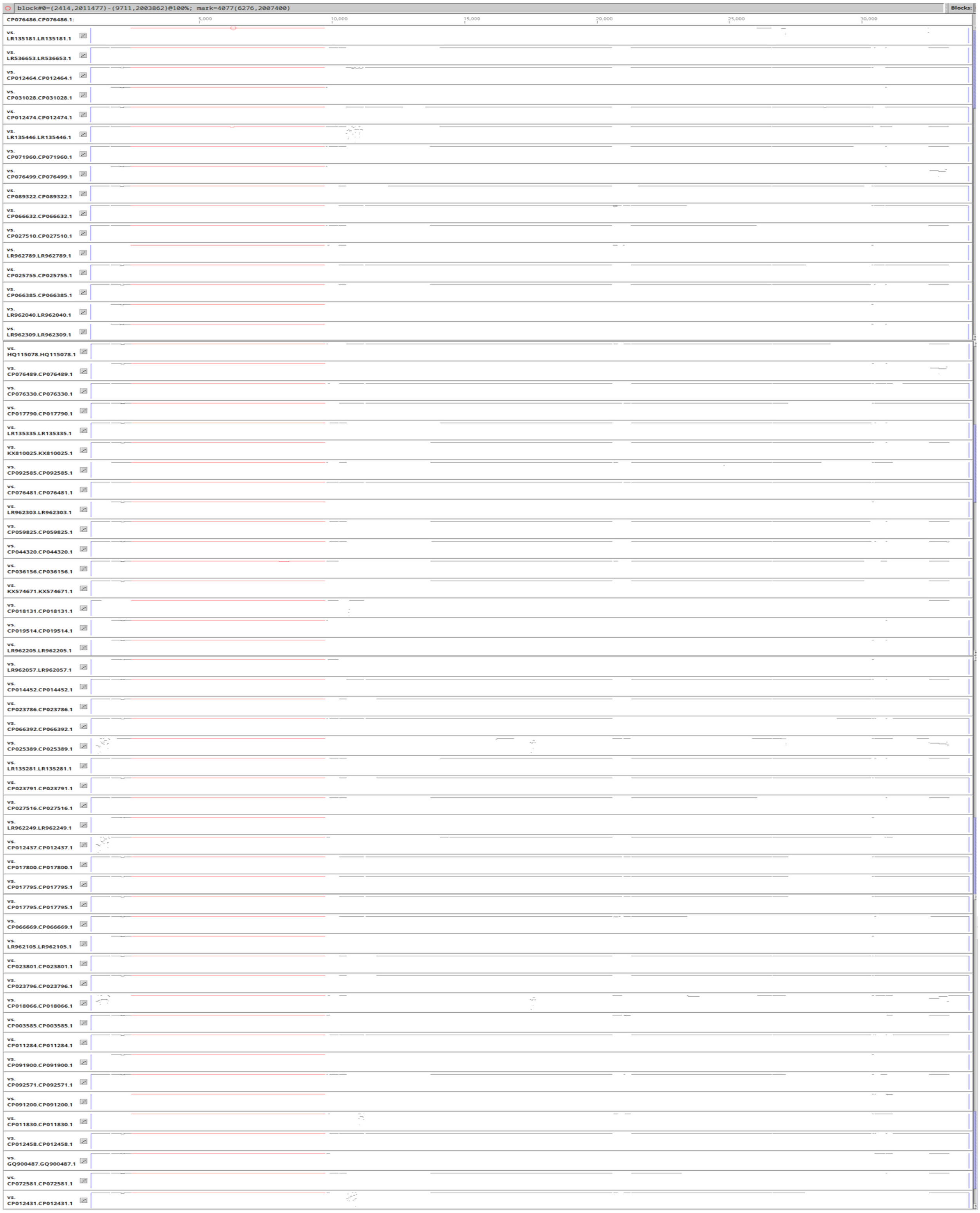

Supplementary Figure 7. Multiple genome alignment of *E. feacium* and *E. faecalis* replicons in which region represented in Figure 3C was preserved. Alignments were visualized using Gmaj software (https://globin.bx.psu.edu/dist/gmaj/). Plasmid pUAMSEF1a from UAMS_EF55 was used as reference for Gmaj software. Regions that align with coordinates 147493..154528 from pUAMSEF4 are market in red color. A zoomable version of this figure is also available at the following DOI on Zenodo: https://doi.org/10.5281/zenodo.6513079

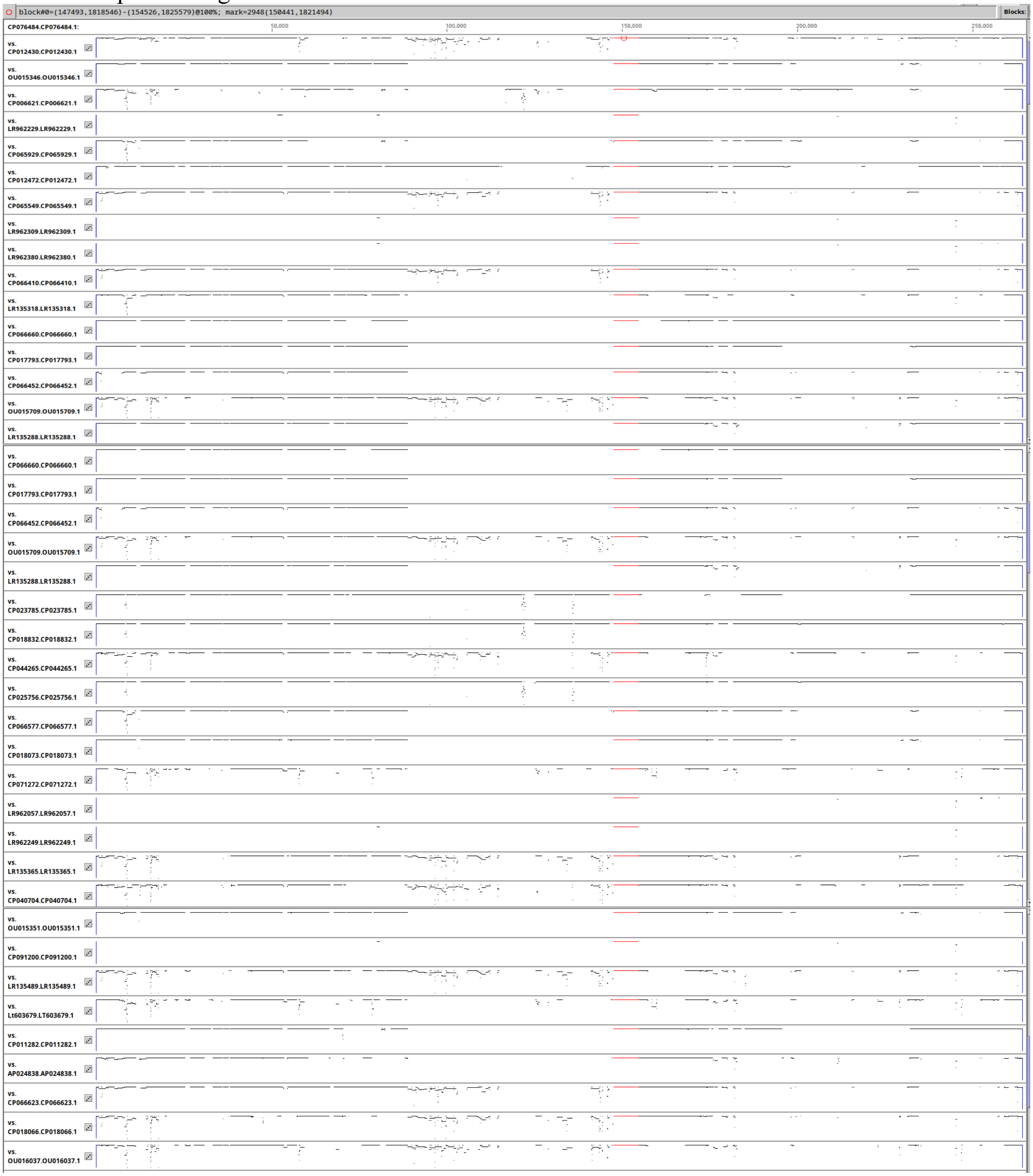

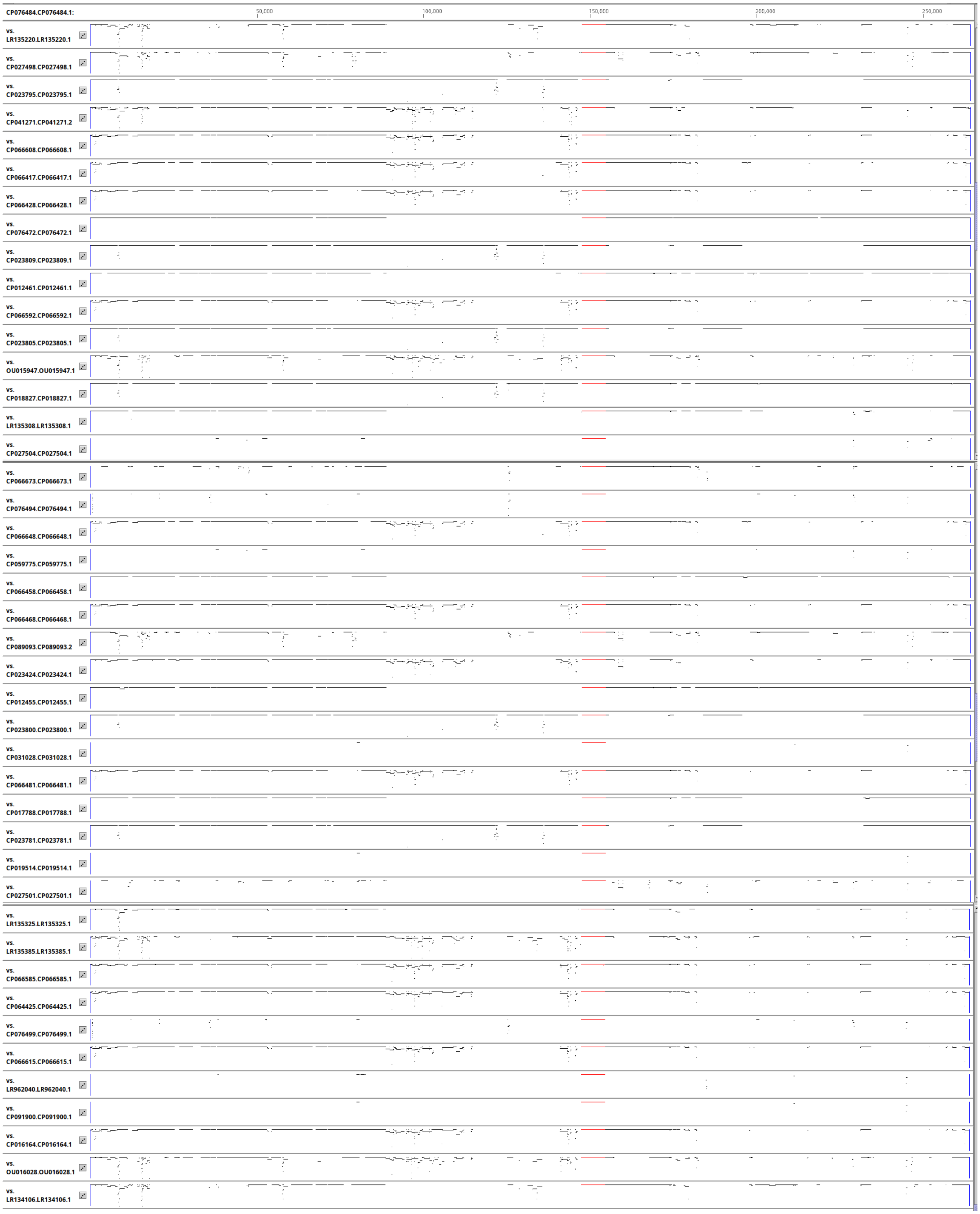

CP076484.CP076484.1:
50,000
100,000
150,000
200,000
250,000
vs. LR135220.LR135220.1
vs. CP027498.CP027498.1
vs. CP023795.CP023795.1
vs. CP041271.CP041271.2
vs. CP066608.CP066608.1
vs. CP066417.CP066417.1
vs. CP066428.CP066428.1
vs. CP076472.CP076472.1
vs. CP023809.CP023809.1
vs. CP012461.CP012461.1
vs. CP066592.CP066592.1
vs. CP023805.CP023805.1
vs. OU015947.OU015947.1
vs. CP018827.CP018827.1
vs. LR135308.LR135308.1
vs. CP027504.CP027504.1
vs. CP066673.CP066673.1
vs. CP076494.CP076494.1
vs. CP066648.CP066648.1
vs. CP059775.CP059775.1
vs. CP066458.CP066458.1
vs. CP066468.CP066468.1
vs. CP089093.CP089093.2
vs. CP023424.CP023424.1
vs. CP012455.CP012455.1
vs. CP023800.CP023800.1
vs. CP031028.CP031028.1
vs. CP066481.CP066481.1
vs. CP017788.CP017788.1
vs. CP023781.CP023781.1
vs. CP019514.CP019514.1
vs. CP027501.CP027501.1
vs. LR135325.LR135325.1
vs. LR135385.LR135385.1
vs. CP066585.CP066585.1
vs. CP064425.CP064425.1
vs. CP076499.CP076499.1
vs. CP066615.CP066615.1
vs. LR962040.LR962040.1
vs. CP091900.CP091900.1
vs. CP016164.CP016164.1
vs. OU016028.OU016028.1
vs. LR134106.LR134106.1

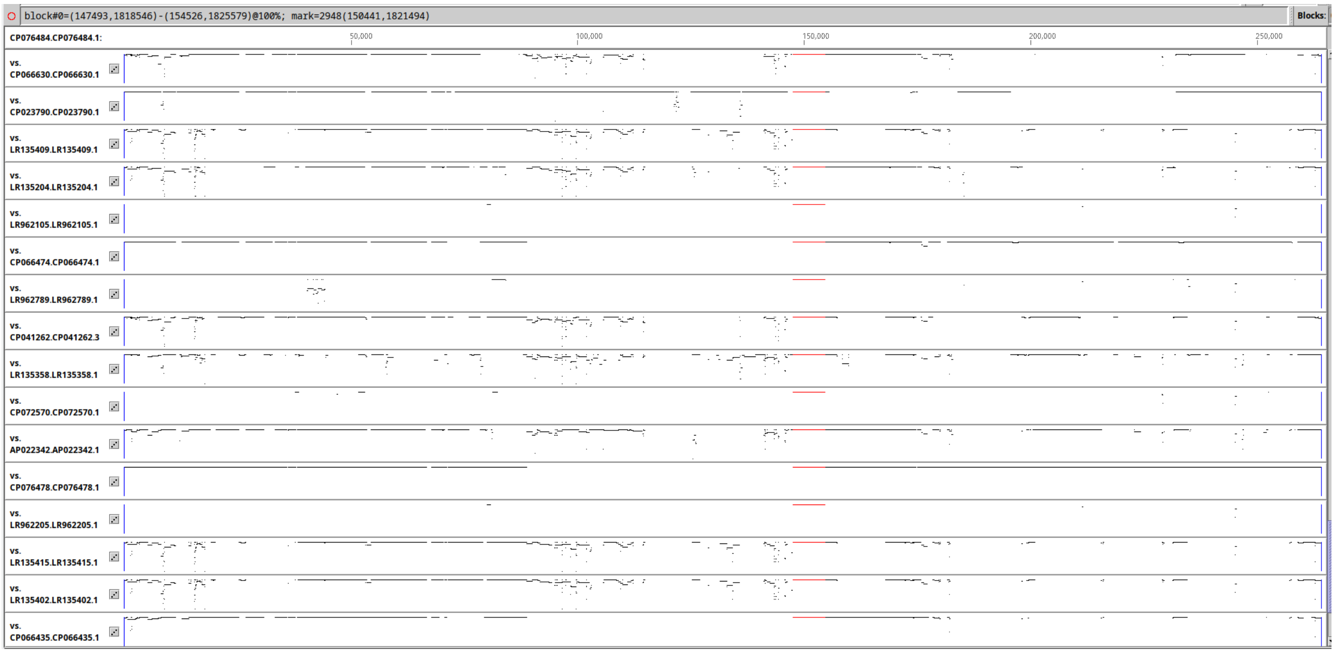

block#0=(147493,1818546)-(154526,1825579)@100%; mark=2948(150441,1821494)
Blocks:
CP076484.CP076484.1:
50,000
100,000
150,000
200,000
250,000
vs. CP066630.CP066630.1
vs. CP023790.CP023790.1
vs. LR135409.LR135409.1
vs. LR135204.LR135204.1
vs. LR962105.LR962105.1
vs. CP066474.CP066474.1
vs. LR962789.LR962789.1
vs. CP041262.CP041262.3
vs. LR135358.LR135358.1
vs. CP072570.CP072570.1
vs. AP022342.AP022342.1
vs. CP076478.CP076478.1
vs. LR962205.LR962205.1
vs. LR135415.LR135415.1
vs. LR135402.LR135402.1
vs. CP066435.CP066435.1

Supplementary Figure 8. Overview of the alignment of the Oxford Nanopore reads from UAMS_EF58 against pUAMSEF4 plasmid from UAMS_EF55. The area of the IS-mediated excision event that was deleted in pUAMSEF4 plasmid from UAMS_EF58 isolate is pointed out. Note that the lowest nanopore read depth in that area was >600x.

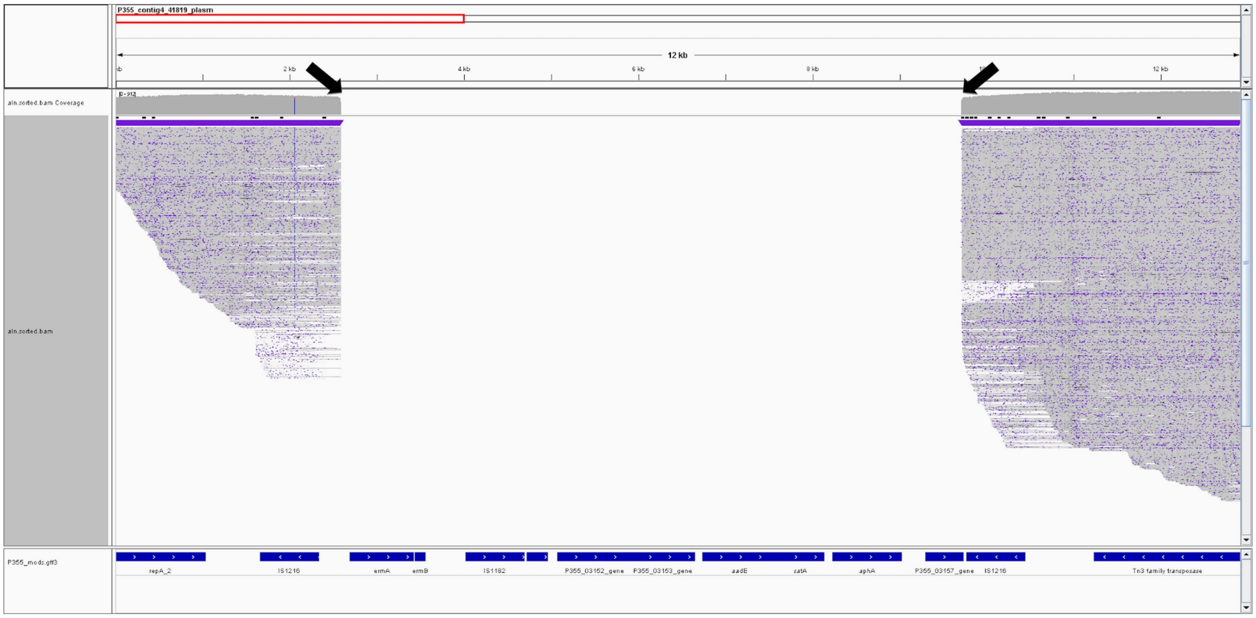

Supplementary Figure 9. Overview of the alignment of the *vanA* cluster from *E. faecalis* isolates against the Blastn database. The *vanA* cluster from our isolates, did not completely align with any of the genomes from the nr/nt nucleotide collection from NCBI. Higher similarities were found against the *Enterococcus saigonensis vanA* cluster. A zoomable version of this figure is also available at the following DOI on Zenodo: https://doi.org/10.5281/zenodo.6513079

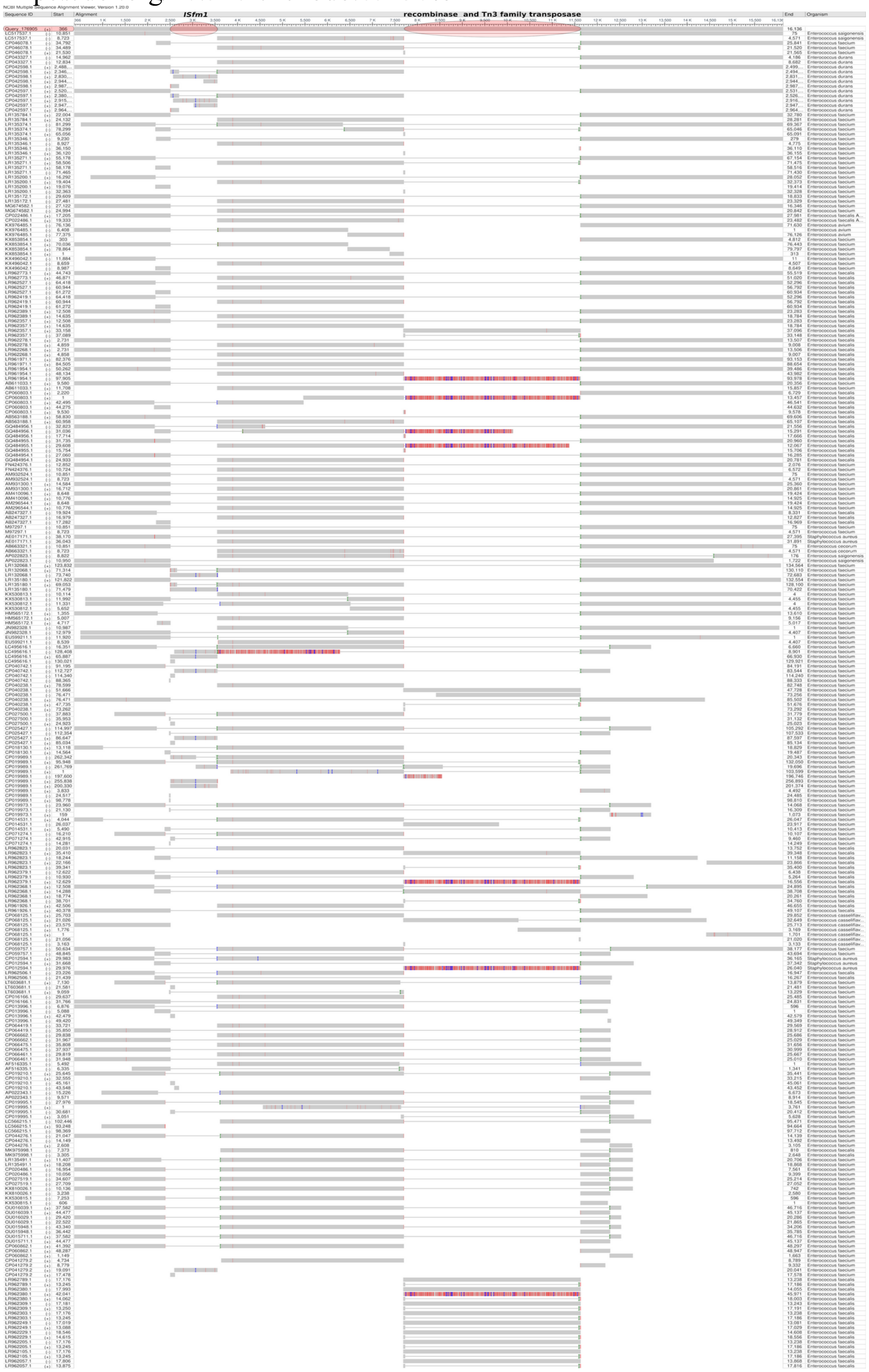

Supplementary Tables are available on Zenodo under DOI:10.5281/zenodo.6392017
Link: https://zenodo.org/record/6392017#.YkIyNiSZNhE
Supplementary Figure 6,7 and 9 are available on Zenodo under DOI: 10.5281/zenodo.6513079
Link:https://doi.org/10.5281/zenodo.6513079

Supplementary Tables Legends:

Supplementary Table 1. Clinical and epidemiological metadata from a patient with bacteremia and the six isolates used in this study.
Supplementary Table 2. Annotated insertion sequences in each of the *E. faecalis* and *E. faecium* isolates using ISEScan.
Supplementary table 3. Annotation tables of the six isolates. The functional annotation was performed using Prokka (4), Antimicrobial resistance (AMR) determinants by RGI against the CARD database (5), and IS families annotated by ISEScan (6) were included and sorted by replicon coordinates. ISs are highlighted in green and ARDs in orange color. This table also contain detailed information about the excision events related to pUAMSEF1a, pUAMSEF3 and pUAMSEF4 plasmids.
Supplementary Table 4. Relation of genes and ISs in LiaFSR gene cluster from 33 complete *E. faecium* genomes that harbor mutations in *liaSR* genes (LiaS^{T120A}, LiaR^{W73C}) surrounded by IS*L3* elements and their genetic neighborhood (±10 genes).
Supplementary Table 5. Results from the BlastN alignments performed using the nucleotide sequences extracted from the two regions identified in the *E. faecalis* and *E. faecium* isolates of this study as putative IS-mediated recombination events between *E. faecalis* and *E. faecium* species.
